# Preparation of Pincer Hafnium Complexes for Olefin Polymerization

**DOI:** 10.3390/molecules24091676

**Published:** 2019-04-29

**Authors:** Su Jin Kwon, Jun Won Baek, Hyun Ju Lee, Tae Jin Kim, Ji Yeon Ryu, Junseong Lee, Eun Ji Shin, Ki Soo Lee, Bun Yeoul Lee

**Affiliations:** 1 Department of Molecular Science and Technology, Ajou University, Suwon 16499, Korea; ksj9355@ajou.ac.kr (S.J.K.); btw91@ajou.ac.kr (J.W.B.); hjulee4639@ajou.ac.kr (H.J.L.); playing3457@ajou.ac.kr (T.J.K.); 2 Department of Chemistry, Chonnam National University, 77 Yongbong-ro, Buk-gu, Gwangju 500-757, Korea; jy5330@naver.com (J.Y.R.); leespy@chonnam.ac.kr (J.L.); 3 LG Chem, Ltd., 188, Munji-ro, Yuseong-gu Daejeon 305-738, Korea; eunjis@lgchem.com (E.J.S.); leekisoo@lgchem.com (K.S.L.)

**Keywords:** pincer complex, hafnium complex, post-metallocene, olefin polymerization

## Abstract

Pincer-type [C^naphthyl^, N^pyridine^, N^amido^]HfMe_2_ complex is a flagship among the post-metallocene catalysts. In this work, various pincer-type Hf-complexes were prepared for olefin polymerization. Pincer-type [N^amido^, N^pyridine^, N^amido^]HfMe_2_ complexes were prepared by reacting in situ generated HfMe_4_ with the corresponding ligand precursors, and the structure of a complex bearing 2,6-Et_2_C_6_H_3_N^amido^ moieties was confirmed by X-ray crystallography. When the ligand precursors of [(CH_3_)R_2_Si-C_5_H_3_N-C(H)PhN(H)Ar (R = Me or Ph, Ar = 2,6-diisopropylphenyl) were treated with in situ generated HfMe_4_, pincer-type [C^silylmethyl^, N^pyridine^, N^amido^]HfMe_2_ complexes were afforded by formation of Hf-CH_2_Si bond. Pincer-type [C^naphthyl^, S^thiophene^, N^amido^]HfMe_2_ complex, where the pyridine moiety in the flagship catalyst was replaced with a thiophene unit, was not generated when the corresponding ligand precursor was treated with HfMe_4_. Instead, the [S^thiophene^, N^amido^]HfMe_3_-type complex was obtained with no formation of the Hf-C^naphthyl^ bond. A series of pincer-type [C^naphthyl^, N^pyridine^, N^alkylamido^]HfMe_2_ complexes was prepared where the arylamido moiety in the flagship catalyst was replaced with alkylamido moieties (alkyl = iPr, cyclohexyl, *t*Bu, adamantyl). Structures of the complexes bearing isopropylamido and adamantylamido moieties were confirmed by X-ray crystallography. Most of the complexes cleanly generated the desired ion-pair complexes when treated with an equivalent amount of [(C_18_H_37_)_2_N(H)Me]^+^[B(C_6_F_5_)_4_]^−^, which showed negligible activity in olefin polymerization. Some complexes bearing bulky substituents showed moderate activities, even though the desired ion-pair complexes were not cleanly afforded.

## 1. Introduction

**Figure 1:**
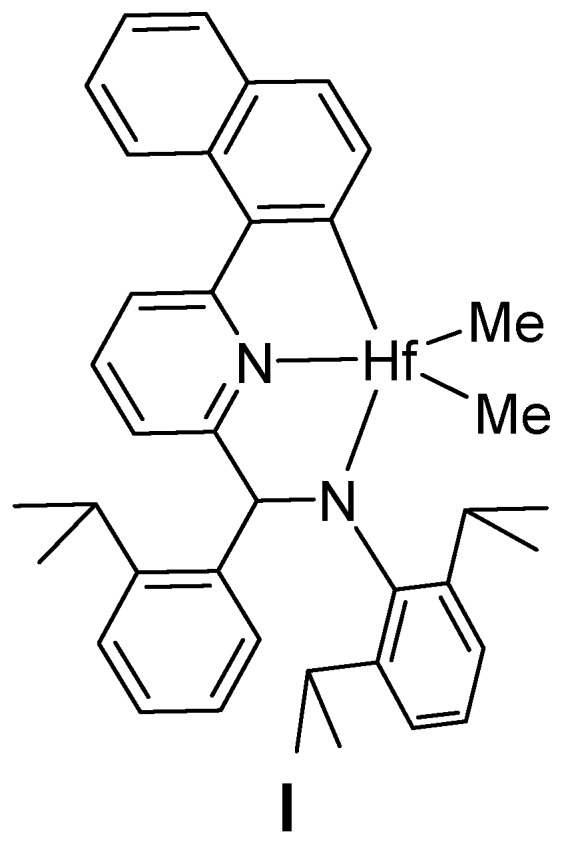
A flagship catalyst in the post-metallocenes.

Transition metal pincer complexes have been prepared to discover applications in various areas, especially in organometallic catalysis [1,2]. The tridentate chelating pincer ligand binds a metal in a meridional fashion to form a coplanar structure with the metal at the center. The ligand-metal interaction is tight and inflexible, which confers high stability. For homogeneous olefin polymerization, the initial zirconium (Zr)-based metallocene catalysts were followed by titanium (Ti)-based half-metallocenes and subsequently post-metallocenes with non-cyclopentadienyl ligands [3,4,5,6,7,8,9]. Among the post-metallocenes that have been developed, a pincer-type [C^naphthyl^, N^pyridine^, N^amido^]HfMe_2_ complex is a flagship catalyst. The complex was discovered in the early 2000s through high-throughput screening and has since been extensively explored and applied in a commercial process (**I** in Figure 1) [10,11]. The pincer-type Hf complex **I** is able to incorporate a large amount of α-olefin in ethylene/α-olefin copolymerizations [12], and is capable of controlling the tacticity of propylene polymerization to produce isotactic polypropylene [13,14,15]. An unique characteristic of **I** is that the β-elimination process, an intrinsic chain transfer reaction that inevitably occurs in the olefin polymerizations performed with the conventional Zr-based metallocene and Ti-based half metallocene catalysts, is completely prevented [14,15,16]. With these merits, it is possible not only to grow a polyolefin (PO) chain from a Hf-site in a living fashion but also to grow PO chains uniformly from diethylzinc (Et_2_Zn) deliberately added in excess as a chain transfer agent. The latter is termed coordinative chain transfer polymerization (CCTP) [17,18,19]. The CCTP technique is judiciously utilized in the commercial production of olefin block copolymers at the Dow Chemical Company [10,20,21,22]. By performing anionic polymerization of styrene in a one-pot reaction after CCTP with **I**, it is also possible to synthesize polyolefin-polystyrene block copolymers [23,24,25]. In this context, many studies have been performed to detail **I** and to improve the catalytic performance by modifying the skeleton of **I** [26,27,28,29,30,31,32,33,34,35]. With an aim to develop upgraded catalyst for **I**, we prepared various pincer-type Hf-complexes. The results are presented herein.

## 2. Results and Discussion

**Scheme 1:**
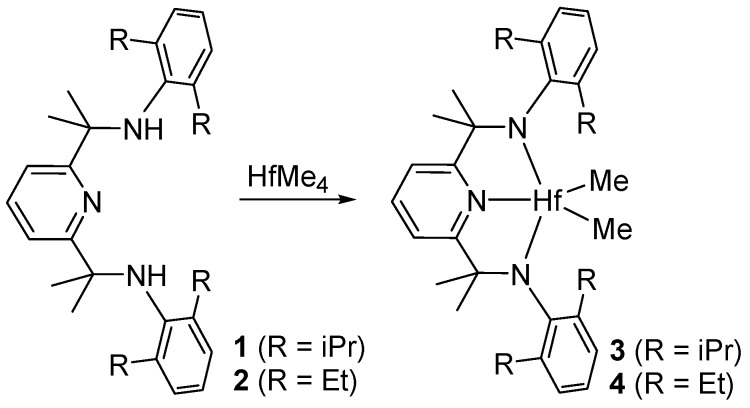
Synthesis of pincer-type [N^amido^, N^pyridine^, N^amido^]HfMe_2_ complexes.

Pincer-type [N^amido^, N^pyridine^, N^amido^]HfMe_2_ complexes were prepared (**3** and **4** in Scheme 1). Bis(amino)pyridine compounds **1** and **2** were prepared according to the reported method involving the stepwise methylation of the corresponding bis(imino)pyridine compounds with AlMe_3_ [36]. Zr and Y complexes have been successfully prepared using **1** [36], but the synthesis of its Hf analogue has not been reported. When **1** and **2** were treated with HfMe_4_ generated by the treatment of HfCl_4_ with 4 equiv methylmagnesium bromide (MeMgBr) at –30 °C, the desired pincer-type Hf-complexes **3** and **4** were cleanly generated [37]. In ^1^H NMR spectra of **3** and **4**, Hf(C*H*_3_)_2_ signals were observed as a singlet at 0.36 and 0.10 ppm, respectively. The structure of **4** was unambiguously confirmed by X-ray crystallography. Metallation of the bis(imino)pyridine compound containing 2,6-Me_2_C_6_H_3_N(H)-moieties was unsuccessful under the same reaction conditions.

**Scheme 2:**
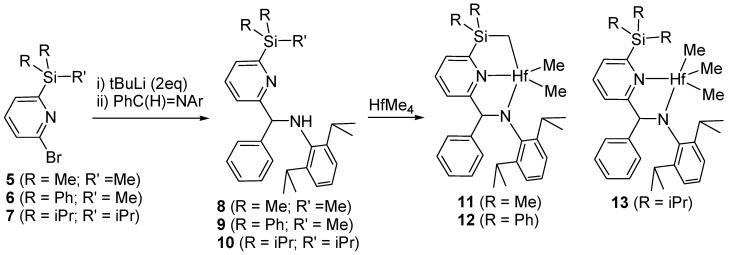
Synthesis of pincer-type [C^silylmethyl^, N^pyridine^, N^amido^]HfMe_2_ complexes.

The prototype complex **I** characteristically contains a Hf-C(aryl) bond. We prepared the related pincer-type [C^silylmethyl^, N^pyridine^, N^amido^]HfMe_2_ complexes containing the Hf-CH_2_Si bond instead of the Hf-C(aryl) bond (Scheme 2). 2-Br-6-(R_2_R’Si)-pyridines (R = Me, Ph, iPr; R’ = Me, iPr; **5**–**7**) were prepared from 2,6-dibromopyridine by the treatment of *n*BuLi and subsequently Me_3_SiCl, Ph_2_(Me)SiCl, or iPr_3_Si(OSO_2_CF_3_) [38,39]. Compounds **5**–**7** were treated with 2 equiv *t*BuLi to generate 2-Li-6-(R_2_R’Si)-pyridines, which were reacted with imine compound PhC(H)=N(2,6-iPr_2_C_6_H_3_) to generate pyridines substituted with the R_2_R’Si-group and (2,6-iPr_2_C_6_H_3_)N(H)C(Ph)(H)-group at the 2- and 6-positions (**8**–**10**). Treatment of **8** and **9** with in situ generated HfMe_4_ afforded the targeted pincer-type [C^silylmethyl^, N^pyridine^, N^amido^]HfMe_2_ complexes containing the Hf-CH_2_Si bond (**11** and **12**). The ^1^H NMR spectrum of **11** distinguished the two diastereotopic protons on HfC*H*_2_Si moiety at 1.31 and 0.24 ppm as doublets with a large geminal coupling constant (*J* = 12.6 Hz) (Figure S7 in Supporting Information). The two methyl groups attached on Si and the two methyl groups attached on Hf were also diastereotopic, respectively, and four singlet methyl signals were observed at 0.84, 0.42, 0.37, and 0.27 ppm. The same signal pattern was observed in the ^1^H NMR spectrum of **12**. In contrast, a totally different pattern was observed in the ^1^H NMR spectrum of the product derived from **10**, which had an iPr_3_Si-substituent. Analysis of the spectrum indicated that the Si(Me)_2_C-Hf bond was not formed, while [N^pyridine^, N^amido^]HfMe_3_ complex **13** was generated (Figure S9). The σ-bond metathesis via agostic interaction of SiC-H bond might be a process for the formation of SiC-Hf bond. In the case of iPr_3_Si-substituent, steric hindrance might hamper the agostic interaction not to afford the desired pincer-type complex.

**Scheme 3:**
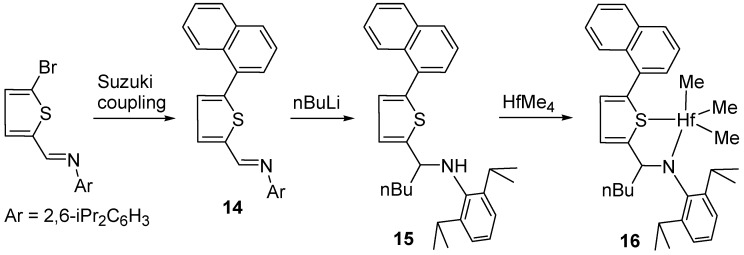
Attempt to synthesize pincer-type [C^naphthyl^, S^thiophene^, N^amido^]HfMe_2_ complex.

Synthesis of pincer-type [C^naphthyl^, S^thiophene^, N^amido^]HfMe_2_ complex was attempted by replacing the pyridine moiety in **I** with thiophene (Scheme 3). The Suzuki-coupling reaction of 1-naphthylboronic acid with the imine compound constructed with 5-bromo-2-thiophencarboxaldehyde and 2,6-iPr_2_C_6_H_3_NH_2_ generated the thiophene compound bearing imine (2,6-iPr_2_C_6_H_3_N=C(H)-) and naphthyl moieties (**14**). In the synthesis of **I**, 2-iPrC_6_H_4_Li facilely attacked the imine group to afford the desired ligand precursor. In the case of the reaction between 2-iPrC_6_H_4_Li and thiophene analogue **14**, the desired ligand precursor was not obtained. However, *n*BuLi readily reacted with **14** to produce the desired thiophene compound **15** substituted with naphthyl and (2,6-iPr_2_C_6_H_3_)N(H)C(nBu)(H)-group at the 2- and 5-positions. When **15** was reacted with HfMe_4_, the Hf-C^aryl^ bond was not formed, which failing to generate the desired [C^naphthyl^, S^thiophene^, N^amido^]HfMe_2_ complex. Instead, the [N^amido^, S^thiophene^]HfMe_3_ complex **16** was cleanly obtained. S-C-C^ipso^(naphthyl) angle might be too wide to generate the desired pincer-type complex via formation of Hf-C(naphthyl) bond. A single signal assigned to Hf(CH_3_)_3_ was observed as a singlet at 0.35 ppm in the ^1^H NMR spectrum (Figure S12).

**Scheme 4:**
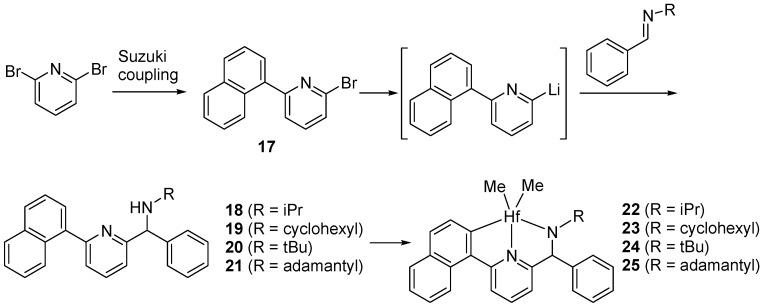
Synthesis of the derivatives of **I** bearing alkylamino moieties.

The prototype complex **I** was discovered through the high-throughput screening and a variety of derivatives were prepared for screening [11,40,41]. The starting material for **I** (6-bromo-2-pyridinecarboxaldehyde) is expensive. The naphthyl group is introduced by the Suzuki-coupling reaction with naphthylboronic acid, and the aldehyde group is converted by condensation with aniline derivatives to imine. This group is reactive with 2-isopropylphenyllithium. The imine bond that forms with alkylamine is easily hydrolyzed in the presence of moisture. Therefore, compounds prepared using alkylamine were not included in the screening. In this work, we prepared the derivatives of **I** containing various alkylamido moieties instead of the arylamido moiety in **I**. The synthetic scheme was different from that developed for **I** and the starting material, 2,6-dibromopyridine, was relatively inexpensive (Scheme 4). 2-Bromo-6-naphthylpyridine **17**, which was prepared through the Suzuki-coupling reaction of 2,6-dibromopyridine and 1-naphthylboronic acid [42], was treated with 2 equiv *t*BuLi to generate 2-lithio-6-naphthylpyridine, which was subsequently reacted with the imines generated through the condensation of benzaldehyde and various alkylamines. The resulting alkylamine compounds **18**–**21** were purified by the conventional column chromatography using silica gel. When **18**–**21** were treated with HfMe_4_, the desired pincer-type [C^naphthyl^, N^pyridine^, N^alkylamido^]HfMe_2_ complexes **22**–**25** were cleanly generated. ^1^H and ^13^C NMR spectra agreed with the structures (Figures S17–S20) and the structure of **22** and **25** were unambiguously confirmed by X-ray crystallography.

### 2.1. X-ray Crystallographic Studies

**Figure 2:**
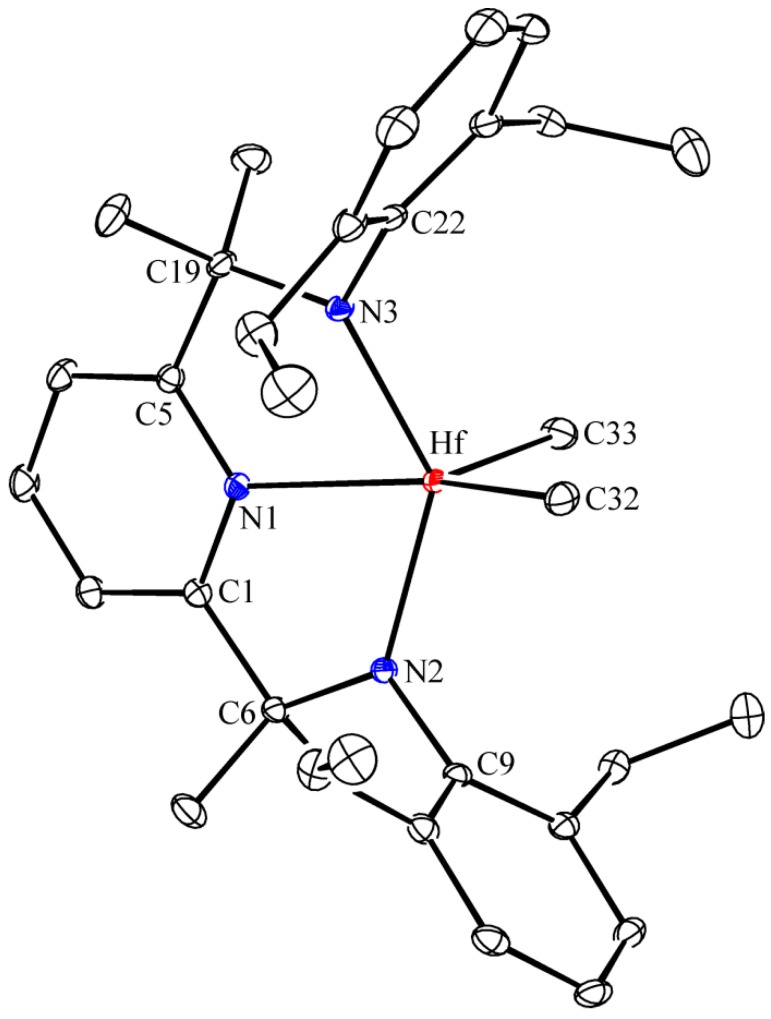
Thermal ellipsoid plot (30% probability level) of **4**. Hydrogen atoms are omitted for clarity. Selected bond distances (Å) and angles (°): Hf-N(1), 2.267(2); Hf-N(2), 2.084(2); Hf-N(3), 2.088(2); Hf-C(33), 2.233(2); Hf-C(32), 2.248(2); C(32)-Hf-N(1), 147.82(7); C(33)-Hf-N(1), 104.18(7); C(33)-Hf-C(32), 107.98(8); N(2)-Hf-N(3), 137.10(6); C(9)-N(2)-C(6), 114.86(14); C(6)-N(2)-Hf, 125.51(11); C(9)-N(2)-Hf, 119.62(11); C(22)-N(3)-Hf, 119.95(11); C(22)-N(3)-C(19), 115.10(14); C(19)-N(3)-Hf, 124.94(11).

The molecular structure of pincer-type [N^amido^, N^pyridine^, N^amido^]HfMe_2_ complexes **4** was confirmed by X-ray crystallography (Figure 2). The geometry around the Hf-center can be defined as a distorted trigonal bipyramid with a basal plane formed by pyridine-N(1), methyl-C(32), and methyl-C(33), with the axial sites occupied with amido N(2) and N(3) atoms. The sum of the bond angles of C(32)-Hf-N(1), C(33)-Hf-N(1), and C(33)-Hf-C(32) is 360°, indicating that the Hf atom is perfectly situated in the basal plane. The N(2)-Hf-N(3) angle is 137.10(6)°, which deviated from the angle of 180° expected for the ideal trigonal bipyramidal structure. The Hf atom is not situated in a plane formed by the chelating ligand framework (i.e., a plane formed by N(2), C(6), C(1), N(1), C(5), C(19), and N(3) atoms), but rather is situated slightly above the plane (0.46 Å). The sum of the bond angles around the amido N(2) and N(3) atoms is 360°, respectively, indicating that both N atoms adopt an sp^2^ hybridization for π-donation from N to Hf-center. Hf-N^amido^ (i.e., Hf-N(2) and Hf-N(3)) distances are significantly shorter than that of Hf-N^pyridine^ (i.e., Hf-N(1)) (2.08 vs. 2.27 Å).

**Figure 3:**
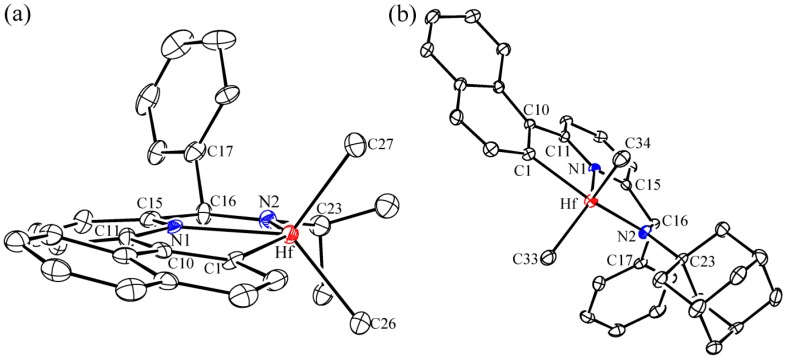
Thermal ellipsoid plot (30% probability level) of **22** (a) and **25** (b). Hydrogen atoms are omitted for clarity. Selected bond distances (Å) and angles (°) in **22** (a): Hf-N(1), 2.296(10); Hf-N(2), 2.065(11); Hf-C(1), 2.285(12); Hf-C(26), 2.203(14); Hf-C(27), 2.223(14); C(27)-Hf-N(1), 122.7(5); C(26)-Hf-N(1), 132.3(5); C(26)-Hf-C(27), 103.9(6); N(2)-Hf-C(1), 139.2(4); C(16)-N(2)-Hf, 127.1(8); C(23)-N(2)-Hf, 121.6(9); C(16)-N(2)-C(23), 110.8(11). In **25** (b): Hf-N(1), 2.295(4); Hf-N(2), 2.089(4); Hf-C(1), 2.302(5); Hf-C(33), 2.231(5); Hf-C(34), 2.212(5); C(33)-Hf-N(1), 133.18(18); C(34)-Hf-N(1), 116.31(18); C(34)-Hf-C(33), 108.4(2); N(2)-Hf-C(1), 140.67(16); C(16)-N(2)-Hf, 123.4(3); C(23)-N(2)-Hf, 122.0(3); C(16)-N(2)-C(23), 114.2(4).

The molecular structure of [C^naphthyl^, N^pyridine^, N^amido^]HfMe_2_ complex bearing isopropylamido moiety (**22**) was confirmed by X-ray crystallography (Figure 3a). Geometry around the Hf-center was defined as a distorted trigonal bipyramid with a basal plane formed by pyridine-N(1), methyl-C(26), and methyl-C(27) atoms. The Hf atom is situated in a plane formed by the chelating ligand framework (i.e., a plane formed by N(2), C(16), C(15), N(1), C(11), C(10), and C(1) atoms). Either the naphthalene or the pyridine ring is slightly tilted from the plane formed by the chelating ligand framework (9.5(4)° and 9.6(4)°, respectively). Amido-N(2) atom adopts sp^2^ hybridization for π-donation and, accordingly, the *C*H(iPr) atom is almost coplanar with the plane formed by the chelating ligand framework (C(15)-C(16)-N(2)-C(23) torsional angle, 171°). The Hf-C^aryl^ distance is slightly longer than Hf-C^methyl^ distances (2.29 vs. 2.20 or 2.22 Å). Molecular structure of **25** bearing the adamantylamido moiety was also confirmed by X-ray crystallography (Figure 3b). In this structure, N-C-C-N chelating ligand framework and pyridine ring form a plane with the Hf-center, while the naphthalene ring is rather severely tilted from the plane (20.0°). While the Hf-N^pyridine^ distance is almost the same with that in **22**, the Hf-N^amido^ and Hf-C^naphthyl^ distances are longer by 0.01 to 0.03 Å than the corresponding distances in **22**.

### 2.2. Activation Reactions

Activation reaction of the prototype complex **I** is complex [43,44]. Reaction with B(C_6_F_5_)_3_ results in decomposition through a process involving C_6_F_5_-transfers. Reaction with [Ph_3_C]^+^[B(C_6_F_5_)_4_]^−^ immediately affords the targeted ion-pair complex {[N,N,C^naphthyl^]HfMe}^+^[B(C_6_F_5_)_4_]^−^. However, the complex is unstable. Reaction with [PhN(H)Me_2_]^+^[B(C_6_F_5_)_4_]^−^ results in the formation of an undesirable complex. The best activator proved to be the aliphatic amine based Bronsted acid [(C_18_H_37_)_2_N(H)Me]^+^[B(C_6_F_5_)_4_]^−^, which generated the desired ion-pair complex, which is stable in benzene [21].

**Scheme 5:**
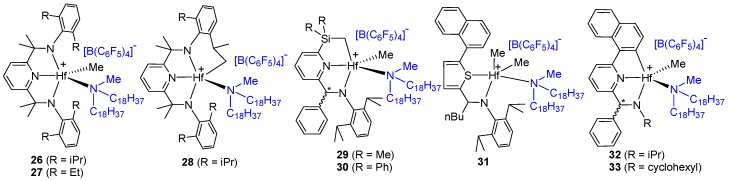
Complexes generated by the action of [(C_18_H_37_)_2_N(H)Me]^+^[B(C_6_F_5_)_4_]^−^.

**Figure 4:**
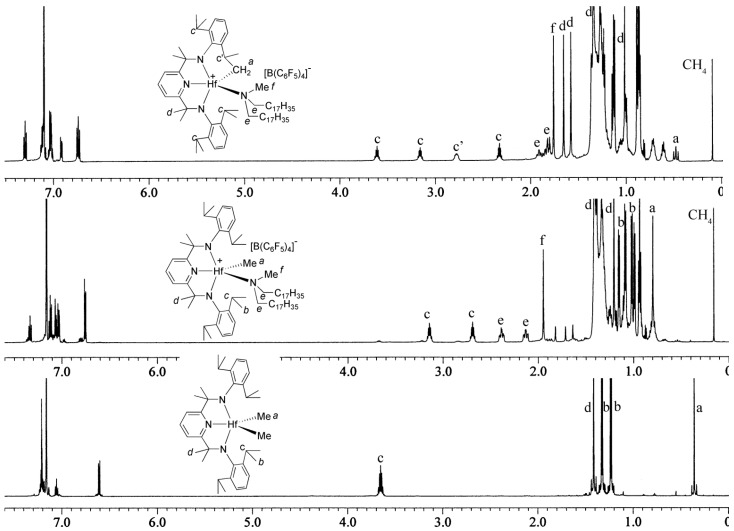
^1^H NMR spectra of **3** and its reaction complexes with [(C_18_H_37_)_2_N(H)Me]^+^[B(C_6_F_5_)_4_]^−^ (**26** and **28**).

When **3** was treated with [(C_18_H_37_)_2_N(H)Me]^+^[B(C_6_F_5_)_4_]^−^ in C_6_D_6_, methide abstraction occurred as was evident from the observation of the methane signal at 0.16 ppm in the ^1^H NMR spectrum. In the ^1^H NMR spectrum collected at the early stage of the reaction, two sets of signals corresponding to the ligand framework were observed in a ratio of approximately 10:1 (Figure 4). The major set of signals was unambiguously assigned to the desired ion-pair complex **26** (Scheme 5). In the ^1^H NMR spectrum of **3**, all four isopropyl groups were equivalent and a single Me_2_C*H*-resornace was observed at 3.65 ppm as a septet. However, in the ^1^H NMR spectrum of the desired ion-pair complex **26**, Me_2_C*H*-resonances were split as a pair of signals at 3.14 and 2.69 ppm, which was attributed to the persistent coordination of (C_18_H_37_)_2_NMe to the Hf-center. With the persistent coordination of amine, the two protons on an α-methylene of (C_18_H_37_)_2_NMe are diastereotopic to each other, even though the two α-methylene carbons are equivalent. This was reflected as separately observed NC*H*_2_ resonances at 2.39 and 2.13 ppm with a triplet-doublet coupling pattern (*J* = 13 and 4.2 Hz). Due to the persistent coordination of amine, the NC*H*_2_ signals as well as the NC*H*_3_ signal were sharp. With time, the minor set of signals became more prominent as the set of signals mainly observed at the initial stage became depressed and disappeared by 6 h. The spectrum was assigned to complex **28** generated from the ion-pair complex **26** through C-H bond activation of a *H*-CH_2_(Me)CH-group (i.e., isopropyl group). This unwanted reaction was also observed in the activation reaction of the prototype complex **I** with [Ph(Me)_2_N-H]^+^[B(C_6_F_5_)_4_]^−^. In the ^1^H NMR spectrum of **28**, three Me_2_C*H*-resonances were observed at 3.67, 3.22, and 2.39 ppm as a septet, while the resonance of the isopropyl group that was engaged in the C-H bond activation process (i.e., Hf-CH_2_(Me)C*H*-signal) was observed at 2.84 ppm as a broad multiplet (Figure 3). In the case of **4**, which contained ethyl substituents instead of isopropyl, the targeted ion-pair complex **27** was also cleanly generated by the action of [(C_18_H_37_)_2_N(H)Me]^+^[B(C_6_F_5_)_4_]^−^ through methide abstraction. In contrast with **3**, the activated complex was stable in C_6_D_6_ with no further reaction.

The reaction of [C^silylmethyl^, N^pyridine^, N^amido^]HfMe_2_ complexes containing the Hf-CH_2_Si bond (**11** and **12**) with [(C_18_H_37_)_2_N(H)Me]^+^[B(C_6_F_5_)_4_]^−^ generated the desired ion-pair complexes **29** and **30** with concomitant generation of methane. Due to presence of chiral centers at the ligand framework as well as the Hf-center, two sets of signals were observed. The reaction rate of **11** was slow, requiring approximately 3 h for complete methide abstraction. At this stage of the complete reaction, the ratio of the two diastereomers was approximately 1:1, which slowly changed and ultimately became 1.0:0.60 after an overnight reaction. In the case of **12**, the reaction rate was rapid and efficiently generated two diastereomers at a ratio of 1.0:0.50 within 30 min. Upon an overnight reaction, the ratio also slowly changed to become 1.0:0.85. The change of the ratio indicated that the site epimerization occurred, even though the rate was slow.

[N^pyridine^, N^amido^]HfMe_3_-type complex **13** facilely reacted with [(C_18_H_37_)_2_N(H)Me]^+^[B(C_6_F_5_)_4_]^−^ to generate methane. However, the ^1^H NMR signals were too complicated to be unambiguously assigned (Figure S24). In contrast, [N^amido^, S^thiophene^]HfMe_3_-type complex **16** reacted slowly with [(C_18_H_37_)_2_N(H)Me]^+^[B(C_6_F_5_)_4_]^−^ and a set of signals eventually appeared after 3 days. These were assigned to **31** (Figure S25). Two Hf-C*H*_3_ signals were observed at 0.60 and 0.41 ppm. Reaction of **22** and **23** bearing secondary amido moieties cleanly afforded the targeted ion-pair complexes **32** and **33**, for which two sets of signals were observed, respectively, in ^1^H NMR spectra, due to the presence of chiral centers on both the Hf-center and at the ligand framework (Figures S26 and S27). Reaction of **24** and **25** bearing tertiary amido moieties did not cleanly afford the targeted ion-pair complexes (Figures S28 and S29).

### 2.3. Polymerization Studies

The targeted ion-pair complex **27** derived from [N^amido^, N^pyridine^, N^amido^]HfMe_2_ bearing ethyl substituents (**4**) exhibited moderate activity in ethylene/propylene copolymerization. The activity was approximately 1/7th that of the prototype Dow catalyst **I** (entry 1 vs. 4 in Table 1). All other complexes (i.e., **3**, **11**, **12**, **16**, and **22**–**23**) that showed clean ^1^H NMR signals in the activation reaction with [(C_18_H_37_)_2_N(H)Me]^+^[B(C_6_F_5_)_4_]^−^ exhibited negligible activities. Complex **13** and **25** bearing bulky iPr_3_Si- and adamantyl group, which did not show clean ^1^H NMR signals in the activation reaction, exhibited moderate activities (approximately 1/10th that of **I**; entries 2 and 3). Comonomer incorporation ability was also inferior to that of **I**; 6.7, 0, and 5.1 mol% propylene was incorporated with **4**, **13**, and **25**, respectively, while the propylene content was very high (56 mol%) with **I** under the identical polymerization conditions. [N^pyridine^, N^amido^]HfMe_3_ complex **13** bearing bulky iPr_3_Si-group generated the relatively high-molecular-weight polymer (*M*_n_, 430 kDa). The prototype Hf catalyst **I** was exceptional in terms of the activity and α-olefin incorporation capability. We also reported various types of Hf-complexes ([N,P]Hf(CH_2_Ph)_3_, [N,P,N]HfMe_2_, and [N,N]Hf(CH_2_Ph)_3_-type) with tetrahydroquinoline and tetrahydrophenanthroline framework, which were also inferior to **I** in terms of both activity and α-olefin incorporation capability [45,46,47]. The Hf-C bonding character was significantly ionic when compared to those of Zr-C and Ti-C bonding, and steric congestion around Hf-center might be crucial for the high activity [48]. When steric congestion is not significant, the ionic Hf-C bond becomes strong and the insertion of olefin through the Hf-C bond may be less favorable, leading to lowered activity.

## 3. Materials and Methods

### 3.1. General Remarks

All manipulations were performed under an inert atmosphere using standard glove box and the Schlenk technique. Toluene, hexane, diethyl ether, THF and C_6_D_6_ were distilled from benzophenone ketyl. Methylcyclohexane used for the polymerization reactions was purchased from TCI and was purified over a Na/K alloy. Ethylene was purified by contact with molecular sieves and copper at a pressure of 50 bar. ^1^H NMR (600 MHz) and ^13^C NMR (150 MHz) spectra were recorded using an ECZ 600 apparatus (JEOL, Tokyo, Japan). Compounds **5** [39], **7** [38], **17** [42], 2-iPrC_6_H_4_Li [41], and PhC(H)=NCH(CH_3_)_2_ were prepared according to previously reported procedures and conditions [49].

### 3.2. Complex ***3***

MeMgBr (0.400 mL, 1.20 mmol, 3.0 M solution in diethyl ether) was added dropwise at −78 °C to a solution of HfCl_4_ (93.5 mg, 0.292 mmol) in toluene (2 mL). The resulting solution was stirred at −40 to −35 °C for 1 h to precipitate a white solid. After cooling to −78 °C, a solution of **1** (0.100 g, 0.195 mmol) in toluene was added dropwise. The resulting mixture was stirred at −40 to −35 °C for 2 h and then warmed slowly to room temperature. After stirring overnight, all volatiles were removed using a vacuum line. Toluene (10 mL) was added to extract the product. The extract was collected through filtration over Celite. After the solvent was removed, the residue was triturated with hexane to obtain a pink solid (0.099 g, 70%). The isolated product was contaminated with some amount of chloromethyl-Hf analog, which was converted to the desired dimethyl-Hf complex by treatment with MeLi in toluene. ^1^H NMR (600 MHz, C_6_D_6_): δ 7.26–7.19 (m, 6H), 7.06 (t, *J* = 7.8 Hz, 1H), 6.61 (d, *J* = 8.4 Hz, 2H), 3.65 (sept, *J* = 7.2 Hz, 4H, C*H*(CH_3_)_2_), 1.41 (s, 12H, NC(CH_3_)_2_), 1.32 (d, *J* = 7.2 Hz, 12H, CH(C*H*_3_)_2_), 1.22 (d, *J* = 7.2 Hz, 12H, CH(C*H*_3_)_2_), 0.36 (s, 6H, Hf(CH_3_)_2_) ppm. ^13^C{^1^H} NMR (150 MHz, C_6_D_6_): δ 24.38, 25.21, 26.63, 28.44, 32.98, 57.22, 70.38, 116.34, 124.36, 125.71, 127.98, 140.15, 145.67, 148.54, 173.89 ppm. Anal. Calcd. (C_37_H_55_N_3_Hf): C, 61.69; H, 7.70; N, 5.83. Found: C, 61.54; H, 7.62; N, 5.71%.

### 3.3. Complex ***4***

The complex was prepared by the same procedure and conditions as those employed for **3** using HfCl_4_ (0.158 g, 0.492 mmol), MeMgBr (0.700 mL, 2.08 mmol, 3.0 M solution in diethyl ether), **2** (0.150 g, 0.328 mmol), and toluene (6 mL). A red solid was obtained (0.132 g, 61%). Single crystals suitable for X-ray crystallography were obtained by recrystallization in toluene/hexane cosolvent at −30 °C. ^1^H NMR (600 MHz, C_6_D_6_): δ 7.40 (d, *J* = 7.2 Hz, 4H), 7.19 (t, *J* = 7.2 Hz, 2H), 7.11 (t, *J* = 7.8 Hz, 1H), 7.68 (d, *J* = 8.4 Hz, 2H), 3.13 (quartet, *J* = 7.2 Hz, 4H, C*H*_2_CH_3_), 2.85 (quartet, *J* = 7.2 Hz, 4H, C*H*_2_CH_3_), 1.38–1.22 (m, 24H, CH_2_C*H*_3_, NC(CH_3_)_2_), 0.10 (s, 6H, Hf(CH_3_)_2_) ppm. ^13^C{^1^H} NMR (150 MHz, C_6_D_6_): δ 15.50, 25.82, 31.65, 54.60, 73.10, 117.75, 125.41, 126.36, 139.67, 144.52, 144.97, 173.14 ppm. Anal. Calcd. (C_33_H_47_N_3_Hf): C, 59.67; H, 7.13; N, 6.33. Found: C, 59.86; H, 7.25; N, 6.46%.

### 3.4. 2-Bromo-6-(dimethylphenylsilanyl)pyridine (***6***)

*n*BuLi (1.70 mL, 4.22 mmol, 2.5 M solution in hexane) was added dropwise at −78 °C to a stirred solution of 2,6-dibromopyridine (1.00 g, 4.22 mmol) in diethyl ether (20 mL). The resulting solution was warmed to −40 °C and stirred for 20 min. After cooling to −78 °C, Ph_2_MeSiCl (1.08 g, 4.64 mmol) in diethyl ether (5 mL) was added. After stirring for 3 h, the solution was warmed to room temperature and water (30 mL) was added. The product was extracted with diethyl ether (3 × 10 mL). The collected organic phases were dried over anhydrous MgSO_4_ and the solvent was removed with a rotary evaporator. The product was purified by column chromatography on silica gel using hexane and ethyl acetate (50:1 *v*/*v*). White solid was obtained (1.05 g, 70%). ^1^H NMR (600 MHz, C_6_D_6_): δ 7.58 (d, *J* = 7.2 Hz, 4H), 7.20–7.12 (m, 6H), 7.04 (d, *J* = 7.2 Hz, 1H), 6.91 (d, *J* = 7.8 Hz, 1H), 6.53 (t, *J* = 7.2 Hz, 1H), 0.82 (s, 3H, CH_3_) ppm. ^13^C{^1^H} NMR (150 MHz, C_6_D_6_): δ −3.92, 127.74, 128.34, 129.98, 135.17, 135.71, 136.54, 144.07, 167.94 ppm. IR (neat): ν 478 (C-Br) cm^−1^. HRMS(FAB): *m*/*z* calcd ([M+H]^+^ C_18_H_16_BrNSi) 354.0314. Found: 354.0310.

### 3.5. (2,6-Diisopropylphenyl)-{[6-(trimethylsilanyl)pyridin-2-yl](phenyl)methyl}amine (***8***)

A solution of *t*BuLi (3.76 mL, 6.39 mmol, 1.7 M in pentane) in hexane (4 mL) was added to a solution of **5** (0.735 g, 3.19 mmol) in THF (15 mL) at −78 °C and the resulting solution was stirred for 2 h. A solution of PhC(H)=N(2,6-iPr_2_C_6_H_3_) (0.763 g, 2.87 mmol) in THF (15 mL) was added. After stirring for 3 h, the resulting solution was warmed slowly to room temperature and stirred overnight. Water (30 mL) was added and the product was extracted with ethyl acetate (3 × 20 mL). The organic phases were collected and dried over anhydrous MgSO_4_. The solvent was removed using a rotary evaporator. Purification by column chromatography on silica gel using hexane and toluene (2:1 *v*/*v*) gave a light yellow oil (0.934 mg, 78%). ^1^H NMR (600 MHz, C_6_D_6_): δ 7.50 (d, *J* = 7.8 Hz, 2H), 7.15–7.02 (m, 6H), 7.00 (t, *J* = 7.8 Hz, 1H), 6.93 (t, *J* = 7.8 Hz, 1H), 6.66 (d, *J* = 6.6 Hz, 1H), 5.69 (br s, 1H, NH), 5.24 (s, 1H, NCH), 3.44 (sept, *J* = 6.6 Hz, 2H, C*H*(CH_3_)_2_)), 1.19 (d, *J* = 7.2 Hz, 6H, CH(C*H*_3_)_2_), 1.09 (d, *J* = 7.2 Hz, 6H, CH(C*H*_3_)_2_), 0.30 (s, 9H, CH_3_) ppm. ^13^C{^1^H} NMR (150 MHz, C_6_D_6_): δ −1.76, 24.28, 28.13, 70.17, 121.96, 123.97, 127.07, 127.13, 128.40, 134.42, 142.80, 143.71, 144.39, 162.14, 167.69 ppm. IR (neat): *v* 3361 (N-H) cm^−1^. HRMS(EI): *m*/*z* calcd ([M^+^] C_27_H_36_N_2_Si) 416.2648. Found: 416.2650.

### 3.6. (2,6-Diisopropylphenyl)-{[6-(dimethylphenylsilanyl)pyridin-2-yl](phenyl)methyl}amine (***9***)

The compound was prepared by the same procedure and conditions as those employed for **8** using *t*BuLi (1.66 mL, 2.82 mmol, 1.7 M in pentane), **6** (0.500 g, 1.41 mmol), and PhC(H)=N(2,6-iPr_2_C_6_H_3_) (0.337 g, 1.27 mmol). Purification by column chromatography on silica gel using hexane and toluene (2:1 *v*/*v*) produced a white glassy solid (0.186 g, 27%). ^1^H NMR (600 MHz, C_6_D_6_): δ 7.63 (t, *J* = 7.8 Hz, 4H), 7.46 (d, *J* = 7.2 Hz, 2H), 7.22–7.00 (m, 13H), 6.86 (t, *J* = 7.2 Hz, 1H), 6.66 (d, *J* = 7.2 Hz, 1H), 5.52–5.40 (br, 1H, NH), 5.32–5.20 (br, 1H, NCH), 3.31 (sept, *J* = 6.0 Hz, 2H, C*H*(CH_3_)_2_), 1.35 (d, *J* = 6.6 Hz, 6H, CH(C*H*_3_)_2_), 1.09 (d, *J* = 6.6 Hz, 6H, CH(C*H*_3_)_2_), 0.87 (s, 3H, CH_3_) ppm. ^13^C{^1^H} NMR (150 MHz, C_6_D_6_): δ −3.83, 24.20, 24.45, 28.13, 122.14, 123.94, 127.11, 128.26, 128.42, 129.31, 129.76, 129.81, 134.66, 135.79, 142.72, 143.43, 144.14, 162.61, 164.85 ppm. IR (neat): *v* 3361 (N-H) cm^−1^. HRMS(EI): *m*/*z* calcd ([M^+^] C_37_H_40_N_2_Si) 540.2961. Found: 540.2964.

### 3.7. (2,6-Diisopropylphenyl)-{[6-(triisopropylsilanyl)pyridin-2-yl](phenyl)methyl}amine (***10***)

The compound was prepared by the same procedure and conditions as those employed for **8** using *t*BuLi (0.56 mL, 0.954 mmol, 1.7 M in pentane), **7** (0.150 g, 0.477 mmol), and PhC(H)=N(2,6-iPr_2_C_6_H_3_) (0.114 g, 0.429 mmol). Purification by column chromatography on silica gel using hexane and toluene (2:1 *v*/*v*) produced a light yellow oil (0.161 g, 75%). ^1^H NMR (600 MHz, C_6_D_6_): δ 7.40 (d, *J* = 7.2 Hz, 2H), 7.15–7.05 (m, 6H), 7.22–7.00 (m, 2H), 6.70 (d, *J* = 8.4 Hz, 1H), 5.34–5.21 (br, 2H, NH, NCH), 3.35 (sept, *J* = 7.2 Hz, 2H, C*H*(CH_3_)_2_), 1.47 (sept, *J* = 7.2 Hz, 3H, SiC*H*(CH_3_)_2_), 1.20–1.10 (m, 30H, CH(C*H*_3_)_2,_ SiCH(C*H*_3_)_2_) ppm. ^13^C{^1^H} NMR (150 MHz, C_6_D_6_): δ 11.17, 18.70, 24.09, 24.38, 28.00, 69.90, 121.38, 123.77, 123.96, 126.87, 129.14, 133.86, 142.84, 143.84, 161.94, 164.65 ppm. IR (neat): *v* 3366 (N-H) cm^−1^. HRMS(EI): *m*/*z* calcd ([M^+^] C_33_H_48_N_2_Si) 500.3587. Found: 500.3589.

### 3.8. Complex ***11***

The complex was prepared by the same procedure and conditions as those employed for **3** using HfCl_4_ (0.230 g, 0.718 mmol), MeMgBr (1.00 mL, 2.94 mmol, 3.0 M solution in diethyl ether), and **8** (0.230 g, 0.552 mmol). A yellow oil was obtained (0.220 g, 60%). ^1^H NMR (600 MHz, C_6_D_6_): δ 7.21–7.11 (m, 2H), 7.06 (d, *J* = 7.2 Hz, 1H), 7.00 (d, *J* = 7.8 Hz, 1H), 6.98–6.90 (m, 5H), 6.86 (t, *J* = 7.2 Hz, 1H), 6.55 (d, *J* = 9.0 Hz, 1H), 5.96 (s, 1H, NCH), 3.66 (sept, *J* = 6.6 Hz, 1H, C*H*(CH_3_)_2_), 3.26 (sept, *J* = 6.6 Hz, 1H, C*H*(CH_3_)_2_), 1.38 (d, *J* = 7.2 Hz, 3H, CH(C*H*_3_)_2_), 1.34 (d, *J* = 7.2 Hz, 3H, CH(C*H*_3_)_2_), 1.31 (d, *J* = 12.6 Hz, 1H, SiCH_2_Hf), 1.24 (d, *J* = 7.2 Hz, 3H, CH(C*H*_3_)_2_), 0.84 (s, 3H,SiCH_3_), 0.42 (s, 3H, HfCH_3_), 0.37 (s, 3H,SiCH_3_), 0.35 (d, *J* = 7.2 Hz, 3H, CH(C*H*_3_)_2_), 0.27 (s, 3H, HfCH_3_), 0.24 (d, *J* = 12.6 Hz, 1H, SiCH_2_Hf) ppm. ^13^C{^1^H} NMR (150 MHz, C_6_D_6_): δ 11.17, 18.70, 24.09, 24.38, 28.00, 69.90, 121.38, 123.77, 123.96, 126.87, 129.14, 133.86, 142.84, 143.84, 161.94, 164.65 ppm.

### 3.9. Complex ***12***

The complex was prepared by the same procedure and conditions as those employed for **3** using HfCl_4_ (0.155 g, 0.483 mmol), MeMgBr (0.70 mL, 2.0 mmol, 3.0 M solution in diethyl ether), and **9** (0.174 g, 0.322 mmol). An orange glassy solid was obtained (0.169 g, 84%). ^1^H NMR (600 MHz, C_6_D_6_): δ 7.66 (d, *J* = 5.4 Hz, 2H), 7.61–7.52 (m, 2H), 7.27–7.11 (m, 9H), 7.04 (d, *J* = 7.8 Hz, 1H), 6.95–6.86 (m, 5H), 6.79 (t, *J* = 7.2 Hz, 1H), 6.53 (d, *J* = 8.4 Hz, 1H), 5.95 (s, 1H, NCH), 3.64 (sept, *J* = 6.6 Hz, 1H, C*H*(CH_3_)_2_), 3.29 (sept, *J* = 6.6 Hz, 1H, C*H*(CH_3_)_2_), 1.69 (d, *J* = 13.2 Hz, 1H, SiCH_2_Hf), 1.36 (d, *J* = 7.2 Hz, 3H, CH(C*H*_3_)_2_), 1.32 (d, *J* = 7.2 Hz, 3H, CH(C*H*_3_)_2_), 1.28 (d, *J* = 7.2 Hz, 3H, CH(C*H*_3_)_2_), 0.79 (d, *J* = 13.2 Hz, 3H, SiCH_2_Hf), 0.53 (s, 3H, HfCH_3_), 0.40 (s, 3H, HfCH_3_), 0.31 (d, *J* = 7.2 Hz, 3H, CH(C*H*_3_)_2_) ppm. ^13^C{^1^H} NMR (150 MHz, C_6_D_6_): δ 24.22, 25.19, 26.45, 26.59, 28.04, 28.64, 54.40, 62.43, 64.65, 83.14, 127.82, 128.28, 128.40, 128.83, 129.43, 129.62, 129.77, 131.11, 135.49, 135.52, 136.58, 137.46, 139.82, 144.12, 145.66, 146.34, 146.82, 170.02, 172.44 ppm. Anal. Calcd. (C_39_H_44_N_2_SiHf): C, 62.68; H, 5.93; N, 3.75%. Found: C, 62.73; H, 5.97; N, 3.80%.

### 3.10. Complex ***13***

The complex was prepared by the same procedure and conditions as those employed for **3** using HfCl_4_ (0.071 g, 0.22 mmol), MeMgBr (0.30 mL, 0.91 mmol, 3.0 M solution in diethyl ether), and **10** (0.074 g, 0.15 mmol). Yellow oil was obtained (0.097 g, 91%). ^1^H NMR (600 MHz, C_6_D_6_): δ 7.32 (d, *J* = 7.8 Hz, 2H), 7.19 (d, *J* = 7.8 Hz, 1H), 7.14 (t, *J* = 7.8 Hz, 1H), 7.10 (d, *J* = 7.8 Hz, 1H), 7.05 (d, *J* = 7.2 Hz, 1H), 7.00 (t, *J* = 7.2 Hz, 2H), 6.92 (t, *J* = 7.2 Hz, 1H), 6.79 (t, *J* = 7.2 Hz, 1H), 6.10 (d, *J* = 7.8 Hz, 1H), 5.56 (s, 1H, NCH), 3.82 (sept, *J* = 6.6 Hz, 1H, C*H*(CH_3_)_2_), 3.63 (sept, *J* = 6.6 Hz, 1H, C*H*(CH_3_)_2_), 1.76 (sept, *J* = 7.2 Hz, 3H, SiC*H*(CH_3_)_2_), 1.43 (d, *J* = 7.2 Hz, 3H, CH(C*H*_3_)_2_), 1.42 (d, *J* = 6.6 Hz, 3H, CH(C*H*_3_)_2_), 1.36 (d, *J* = 6.6 Hz, 3H, CH(C*H*_3_)_2_), 1.15 (d, *J* = 7.8 Hz, 9H, SiCH(C*H*_3_)_2_), 1.12 (d, *J* = 7.2 Hz, 9H, SiCH(C*H*_3_)_2_), 0.52 (s, 9H, Hf(CH_3_)_3_), 0.43 (d, *J* = 7.8 Hz, 3H, CH(C*H*_3_)_2_) ppm. ^13^C{^1^H} NMR (150 MHz, C_6_D_6_): δ 12.90, 19.25, 19.56, 24.86, 25.45, 25.73, 26.24, 28.38, 28.41, 61.07, 81.25, 123.02, 124.64, 125.02, 126.54, 127.98, 128.86, 129.18, 131.71, 135.31, 143.56, 144.00, 146.32, 147.25 ppm.

### 3.11. (2,6-Diisopropylphenyl)-(5-naphthalen-1-ylthiophen-2-ylmethylene)amine (***14***)

A Schlenk flask was charged with (5-bromothiophen-2-yl)-N-(2,6-diisopropylphenyl)methanimine (1.00 g, 2.86 mmol), 1-naphthylboronic acid (0.491 g, 2.86 mmol), Na_2_CO_3_ (0.759 g, 7.17 mmol), and toluene (3 mL) under a N_2_ atmosphere. Degassed H_2_O/EtOH (2 mL, 1:1 *v*/*v*) and a solution of (Ph_3_P)_4_Pd (9.0 mg, 0.0080 mmol) in toluene (1 mL) was added. The biphasic solution was heated to 70 °C and vigorously stirred overnight. After cooling to room temperature, the organic phase was collected and washed with H_2_O (5 mL). The collected organic phase was dried over anhydrous MgSO_4_ and the solvent was removed with a rotary evaporator. Purification by recrystallization in methanol at −30 °C gave a yellow solid (0.738 g, 65%). ^1^H NMR (600 MHz, C_6_D_6_): δ 8.22 (d, *J* = 8.4 Hz, 1H), 8.03 (s, 1H, NCH), 7.58 (d, *J* = 8.4 Hz, 1H), 7.52 (d, *J* = 8.4 Hz, 1H), 7.42 (d, *J* = 8.4 Hz, 1H), 7.23–7.16 (m, 3H), 7.15–7.07 (m, 3H), 6.98 (d, *J* = 3.6 Hz, 1H), 6.92 (d, *J* = 3.6 Hz, 1H), 3.24 (sept, *J* = 7.2 Hz, 2H, C*H*(CH_3_)_2_), 1.20 (d, *J* = 6.6 Hz, 12H, CH(C*H*_3_)_2_) ppm. ^13^C{^1^H} NMR (150 MHz, C_6_D_6_): δ 23.63, 28.63, 123.47, 124.83, 125.51, 125.91, 126.40, 127.02, 127.97, 128.53, 128.75, 129.33, 132.03, 132.07, 132.42, 134.44, 138.09, 143.31, 147.29, 149.67, 155.19 ppm. IR (neat): *v* 1619 (C=N) cm^−1^. HRMS(EI): *m*/*z* calcd ([M^+^] C_27_H_27_NS) 397.1864. Found: 397.1865.

### 3.12. (2,6-Diisopropylphenyl)-[1-(5-naphthalen-1-yl-thiophen-2-yl)pentyl]amine (***15***)

*n*BuLi (0.90 mL, 1.4 mmol, 1.61 M solution in hexane) was added dropwise to a solution of **14** (0.500 g, 1.26 mmol) in toluene (10 mL) at room temperature. After stirring for 1 h, water (10 mL) was added and the product was extracted with toluene (3 × 5 mL). The combined organic phases were dried over anhydrous MgSO_4_ and the solvent was removed with a rotary evaporator. The analysis with ^1^H and ^13^C NMR spectra indicated that the obtained crude oil was pure and it was used for the next step without further purification (0.573 g, 100%). ^1^H NMR (600 MHz, C_6_D_6_): δ 8.40 (d, *J* = 8.4 Hz, 1H), 7.64 (d, *J* = 7.2 Hz, 1H), 7.56 (d, *J* = 7.8 Hz, 1H), 7.52 (d, *J* = 6.6 Hz, 1H), 7.32–7.22 (m, 2H), 7.22–7.08 (m, 4H), 6.85 (d, *J* = 3.0 Hz, 1H), 6.53 (d, *J* = 3.6 Hz, 1H), 4.35 (q, *J* = 2.4 Hz, 1H, NCH), 3.35 (sept, *J* = 7.2 Hz, 2H, C*H*(CH_3_)_2_), 2.17–2.09 (m, 1H, C*H*_2_CH_2_CH_2_CH_3_), 2.03–1.94 (m, 1H, C*H*_2_CH_2_CH_2_CH_3_), 1.60–1.47 (m, 2H, CH_2_C*H*_2_CH_2_CH_3_), 1.32 (sext, *J* = 6.6 Hz, 2H, CH_2_CH_2_C*H*_2_CH_3_), 1.25 (d, *J* = 7.2 Hz, 6H, CH(C*H*_3_)_2_), 1.18 (d, *J* = 6.0 Hz, 6H, CH(C*H*_3_)_2_), 0.87 (t, *J* = 7.2 Hz, 3H, CH_2_CH_2_CH_2_C*H*_3_) ppm. ^13^C{^1^H} NMR (150 MHz, C_6_D_6_): δ 14.23, 23.02, 24.48, 28.22, 29.56, 37.35, 60.88, 123.95 124.37, 125.01, 125.56, 126.21, 126.26, 126.68, 127.28, 128.63, 128.72, 132.38, 133.20, 134.53, 140.61, 141.72, 142.93, 148.43 ppm. IR (neat): *v* 3369 (N-H), 2957 1457, 496, 774 cm^−1^. IR (neat): *v* 3369 (N-H) cm^−1^. HRMS(EI): *m*/*z* calcd ([M^+^] C_31_H_35_NS) 455.2647. Found: 455.2645.

### 3.13. Complex ***16***

The complex was prepared by the same procedure and conditions as those employed for **3** using HfCl_4_ (0.229 g, 0.716 mmol), MeMgBr (1.00 mL, 2.94 mmol, 3.0 M solution in diethyl ether), and **17** (0.251 g, 0.551 mmol). A yellow oil was obtained (0.293 g, 80%). ^1^H NMR (600 MHz, C_6_D_6_): δ 8.48 (d, *J* = 7.8 Hz, 1H), 7.63 (d, *J* = 8.4 Hz, 1H), 7.60 (d, *J* = 7.2 Hz, 1H), 7.56 (d, *J* = 8.4 Hz, 1H), 7.32 (t, *J* = 7.8 Hz, 1H), 7.29–7.08 (m, 6H), 7.01 (d, *J* = 1.8 Hz, 1H), 4.72 (t, *J* = 7.2 Hz, 1H, NCH), 4.04 (sept, *J* = 7.8 Hz, 2H, C*H*(CH_3_)_2_), 3.70 (sept, *J* = 7.2 Hz, 2H, C*H*(CH_3_)_2_), 2.00 (q, *J* = 7.2 Hz, 2H, C*H*_2_CH_2_CH_2_CH_3_), 1.34 (d, 3H, *J* = 6.0 Hz, CH(C*H*_3_)_2_), 1.32 (d, 3H, *J* = 6.6 Hz, CH(C*H*_3_)_2_), 1.31 (d, 3H, *J* = 7.2 Hz, CH(C*H*_3_)_2_), 1.26 (d, 3H, *J* = 7.2 Hz, CH(C*H*_3_)_2_), 1.29–1.20 (m, 1H_,_ CH_2_C*H*_2_CH_2_CH_3_), 1.12 (sext, *J* = 7.2 Hz, 2H, CH_2_CH_2_C*H*_2_CH_3_), 1.07–0.07 (m, 1H, CH_2_C*H*_2_CH_2_CH_3_), 0.72 (t, *J* = 7.2 Hz, 3H, CH_2_CH_2_CH_2_C*H*_3_), 0.35 (s, 9H, Hf(CH_3_)_3_) ppm. ^13^C{^1^H} NMR (150 MHz, C_6_D_6_): δ 14.15, 22.81, 24.92, 25.26, 26.09, 26.33, 27.90, 26.33, 27.90, 28.45, 28.86, 38.31, 63.56, 67.07, 124.99, 125.48, 125.61, 126.06, 126.37, 126.88, 128.37, 128.41, 128.63, 128.80, 128.97, 132.18, 132.59, 134.27, 134.59, 141.84, 146.54, 150.09, 150.70 ppm.

### 3.14. Isopropyl[(6-naphthalen-1-yl-pyridin-2-yl)phenylmethyl]amine (***18***)

The compound was prepared by the same procedure and conditions as those employed for **8** using *t*BuLi (0.79 mL, 1.34 mmol, 1.7 M in pentane), **17** (0.190 g, 0.669 mmol), and PhC(H)=NCH(CH_3_)_2_ (0.108 g, 0.736 mmol). Purification by column chromatograph on silica gel using hexane and triethylamine (100:1 *v*/*v*) gave a colorless oil (0.150 g, 64%). ^1^H NMR (600 MHz, C_6_D_6_): δ 8.32 (d, *J* = 8.4 Hz, 1H), 7.68 (d, *J* = 7.8 Hz, 1H), 7.65 (d, *J* = 7.8 Hz, 1H), 7.59 (d, *J* = 7.2 Hz, 2H), 7.52 (d, *J* = 6.0 Hz, 1H), 7.34–7.24 (m, 3H), 7.24–7.11 (m, 4H), 7.11–7.04 (m, 2H), 5.23 (s, 1H, NCH), 2.81 (sept, *J* = 6.0 Hz, 1H, C*H*(CH_3_)_2_), 0.11–0.09 (m, 6H, CH(C*H*_3_)_2_ ppm. ^13^C{^1^H} NMR (150 MHz, C_6_D_6_): δ 23.17, 23.57, 46.43, 66.16, 120.30, 123.12, 125.49, 126.10, 126.46, 126.77, 127.30, 127.85, 128.28, 128.63, 128.72, 129.09, 132.01, 134.58, 136.88, 139.35, 144.40, 159.02, 163.50 ppm. IR (neat): *v* 3311 (N-H) cm^−1^. HRMS(FAB): *m*/*z* calcd ([M+H]^+^ C_25_H_24_N_2_) 353.2018. Found: 353.2015.

### 3.15. Cyclohexyl[(6-naphthalen-1-yl-pyridin-2-yl)phenylmethyl]amine (***19***)

The compound was prepared by the same procedure and conditions as those employed for **8** using *t*BuLi (0.79 mL, 1.34 mmol, 1.7 M in pentane), **17** (0.190 g, 0.669 mmol), and PhC(H)=NC(CH_3_)_3_ (0.119 g, 0.736 mmol). Purification by column chromatograph on silica gel using hexane and triethylamine (100:1 *v*/*v*) gave a colorless oil (0.187 g, 76%). ^1^H NMR (600 MHz, C_6_D_6_): δ 8.34 (d, *J* = 8.4 Hz, 1H), 7.68 (d, *J* = 7.2 Hz, 1H), 7.64 (d, *J* = 7.8 Hz, 1H), 7.59 (d, *J* = 7.8 Hz, 2H), 7.52 (d, *J* = 7.8 Hz, 1H), 7.33–7.24 (m, 3H), 7.24–7.12 (m, 4H), 7.11–7.03 (m, 2H), 5.27 (s, 1H, NCH), 1.07 (s, 9H, C(CH_3_)_3_) ppm. ^13^C{^1^H} NMR (150 MHz, C_6_D_6_): δ 30.25, 51.64, 63.24, 120.46, 122.83, 125.49, 126.42, 126.76, 127.01, 128.69, 129.10, 132.00, 134.61, 136.79, 139.32, 146.89, 158.66, 165.02 ppm. IR (neat): *v* 3302 (N-H) cm^−1^. HRMS(EI): *m*/*z* calcd ([M^+^] C_26_H_26_N_2_) 366.2096. Found: 366.2098.

### 3.16. t-Butyl[(6-naphthalen-1-yl-pyridin-2-yl)phenylmethyl]amine (***20***)

The compound was prepared by the same procedure and conditions as those employed for **8** using *t*BuLi (1.04 mL, 1.76 mmol, 1.7 M in pentane), **17** (0.250 g, 0.880 mmol), and PhC(H)=NC_6_H_11_ (0.181 g, 0.968 mmol). Purification by column chromatograph on silica gel using hexane and triethylamine (*v*/*v*, 100:1) gave a colorless oil (0.238 g, 69%). ^1^H NMR (600 MHz, C_6_D_6_): δ 8.34 (d, *J* = 7.8 Hz, 1H), 7.68 (d, *J* = 7.2 Hz, 1H), 7.64 (d, *J* = 8.4 Hz, 1H), 7.61 (d, *J* = 7.2 Hz, 2H), 7.52 (d, *J* = 6.6 Hz, 1H), 7.36–7.23 (m, 3H), 7.23–7.15 (m, 2H), 7.10–7.04 (m, 2H), 5.32 (s, 1H, NCH), 2.56 (tt, *J* = 9.6, 3.0 Hz, 1H, C_6_H_11_)_,_ 1.94 (d, *J* = 12 Hz, 2H, C_6_H_11_), 1.90 (d, *J* = 12.6 Hz, 2H, C_6_H_11_), 1.59 (s, 2H, C_6_H_11_), 1.41 (s, 1H, C_6_H_11_), 1.19–0.98 (m, 5H, C_6_H_11_) ppm. ^13^C{^1^H} NMR (150 MHz, C_6_D_6_): δ 25.20, 26.62, 33.92, 34.29, 54.33, 65.61, 120.30, 123.12, 125.49, 126.10, 126.44, 126.80, 127.32, 128.63, 128.74, 129.10, 132.02, 134.60, 136.92, 139.36, 144.65 ppm. IR (neat): *v* 3310 (N-H) cm^−1^. HRMS(FAB): *m*/*z* calcd ([M+H]^+^ C_28_H_28_N_2_) 393.2096. Found: 393.2329.

### 3.17. Adamatyl[(6-naphthalen-1-yl-pyridin-2-yl)phenylmethyl]amine (***21***)

The compound was prepared by the same procedure and conditions as those employed for **8** using *t*BuLi (1.04 mL, 1.76 mmol, 1.7 M in pentane), **17** (0.250 g, 0.880 mmol), and PhC(H)=NC_10_H_16_ (0.232 g, 0.968 mmol). Purification by column chromatograph on silica gel using hexane and triethylamine (100:1 *v*/*v*) gave a white glassy solid (0.260 g, 74%). ^1^H NMR (600 MHz, C_6_D_6_): δ 8.38 (d, *J* = 7.8 Hz, 1H), 7.68 (d, *J* = 8.4 Hz, 1H), 7.66–7.59 (m, 4H), 7.53 (d, *J* = 7.2 Hz, 2H), 7.37–7.25 (m, 4H), 7.25–7.18 (m, 3H), 7.12–7.05 (m, 2H), 5.45 (s, 1H, NCH), 2.56 (s, 1H, C_10_H_16_)_,_ 1.90 (s, 3H, C_10_H_16_), 1.67 (t, 6H, *J* = 15 Hz, C_10_H_16_), 1.51 (dd, 6H, *J* = 19.8, 12 Hz, C_10_H_16_) ppm. ^13^C{^1^H} NMR (150 MHz, C_6_D_6_): δ 30.15, 37.03, 44.20, 51.93, 61.16, 120.49, 122.80, 125.49, 126.08, 126.40, 126.87, 126.97, 127.98, 128.39, 128.63, 128.67, 129.10, 132.02, 134.63, 136.79, 139.35 ppm. IR (neat): *v* 3293 (N-H) cm^−1^. HRMS(EI): *m*/*z* calcd ([M^+^] C_32_H_32_N_2_) 444.2565. Found: 444.2563.

### 3.18. Complex ***22***

The complex was prepared by the same procedure and conditions as those employed for **3** using HfCl_4_ (0.171 g, 0.534 mmol), MeMgBr (0.80 mL, 2.2 mmol, 3.0 M solution in diethyl ether), and **18** (0.126 g, 0.356 mmol). A yellow solid was obtained (0.164 g, 82%). Single crystals suitable for X-ray crystallography were obtained by recrystallization in toluene and hexane at −30 °C. ^1^H NMR (600 MHz, C_6_D_6_): δ 8.64 (d, *J* = 7.2 Hz, 1H), 8.27 (d, *J* = 8.4 Hz, 1H), 7.85 (d, *J* = 7.2 Hz, 1H), 7.73 (d, *J* = 8.4 Hz, 1H), 7.43 (d, *J* = 7.2 Hz, 1H), 7.33 (t, *J* = 7.8 Hz, 1H), 7.28 (t, *J* = 7.2 Hz, 1H), 7.20 (d, *J* = 7.2 Hz, 2H), 7.09 (t, *J* = 7.2 Hz, 2H), 7.05 (t, *J* = 7.8 Hz, 1H), 6.73 (t, *J* = 7.8 Hz, 1H), 6.39 (d, *J* = 7.8 Hz, 1H), 5.85 (s, 1H, NCH), 3.98 (sept, *J* = 6.6 Hz, 1H, C*H*(CH_3_)_2_), 1.53 (d, *J* = 3.3 Hz, 3H, CH(C*H*_3_)_2_), 1.02 (d, *J* = 6.0 Hz, 3H, CH(C*H*_3_)_2_), 0.81 (s, 3H, HfCH_3_), 0.77 (s, 3H, HfCH_3_) ppm. ^13^C{^1^H} NMR (150 MHz, C_6_D_6_): δ 22.13, 24.04, 46.31, 59.38, 60.35, 75.52, 119.82, 120.11, 124.11, 125.35, 126.86, 129.09, 129.77, 130.01, 130.62, 133.95, 135.70, 140.23, 144.18, 146.38, 164.45, 170.90, 204.71 ppm. Anal. Calcd. (C_27_H_28_N_2_Hf): C, 58.01; H, 5.05; N, 5.01%. Found: C, 58.10; H, 5.16; N, 5.09%.

### 3.19. Complex ***23***

The complex was prepared by the same procedure and conditions as those employed for **3** using HfCl_4_ (0.131 g, 0.409 mmol), MeMgBr (0.60 mL, 1.7 mmol, 3.0 M solution in diethyl ether), and **19** (0.100 g, 0.272 mmol). A light brown solid was obtained (0.120 g, 79%). ^1^H NMR (600 MHz, C_6_D_6_): δ 8.65 (d, *J* = 7.2 Hz, 1H), 8.27 (d, *J* = 8.4 Hz, 1H), 7.86 (d, *J* = 7.8 Hz, 1H), 7.74 (d, *J* = 8.4 Hz, 1H), 7.42 (d, *J* = 7.8 Hz, 1H), 7.33 (t, *J* = 7.2 Hz, 1H), 7.28 (t, *J* = 7.2 Hz, 1H), 7.25 (d, *J* = 7.2 Hz, 2H), 7.11 (t, *J* = 7.2 Hz, 2H), 7.03 (t, *J* = 7.2 Hz, 1H), 6.74 (t, *J* = 7.2 Hz, 1H), 6.41 (d, *J* = 8.4 Hz, 1H), 5.93 (s, 1H, NCH), 3.56 (tt, *J* = 8.4, 3.6 Hz, 1H, C_6_H_11_), 2.17 (d, *J* = 11.4 Hz, 1H, C_6_H_11_), 2.06 (qd, 1H, *J* = 12, 3.6 Hz, C_6_H_11_), 1.93 (d, 1H, *J* = 12.6 Hz, C_6_H_11_), 1.81 (d, 1H, *J* = 13.8 Hz, C_6_H_11_), 1.61 (d, 1H, *J* = 12 Hz, C_6_H_11_), 1.50 (d, 1H, *J* = 13.2 Hz, C_6_H_11_), 1.25 (qt, 1H, *J* = 12, 3.6 Hz, C_6_H_11_), 1.20–0.97 (m, 3H, C_6_H_11_), 0.84 (s, 3H, HfCH_3_), 0.80 (s, 3H, HfCH_3_) ppm. ^13^C{^1^H} NMR (150 MHz, C_6_D_6_): δ 26.45, 27.08, 27.49, 33.61, 35.43, 55.83, 59.25, 60.41, 75.78, 119.82, 120.10, 124.12, 125.35, 126.85, 129.08, 129.07, 130.00, 130.61, 134.00, 135.70, 140.22, 144.23, 146.53, 164.46, 170.99, 204.72 ppm. Anal. Calcd. (C_30_H_32_N_2_Hf): C, 60.15; H, 5.38; N, 4.68%. Found: C, 60.32; H, 5.51; N, 4.83%.

### 3.20. Complex ***24***

The complex was prepared by the same procedure and conditions as those employed for **3** using HfCl_4_ (0.216 g, 0.675 mmol), MeMgBr (1.00 mL, 2.77 mmol, 3.0 M solution in diethyl ether), and **20** (0.165 g, 0.450 mmol). A yellow solid was obtained (0.209 g, 81%). Single crystals suitable for X-ray crystallography were obtained by recrystallization in toluene and hexane at −30 °C. ^1^H NMR (600 MHz, C_6_D_6_): δ 8.65 (d, *J* = 7.8 Hz, 1H), 8.18 (d, *J* = 8.4 Hz, 1H), 7.83 (d, *J* = 7.2 Hz, 1H), 7.71 (d, *J* = 7.8 Hz, 1H), 7.33–7.24 (m, 4H), 7.05 (t, *J* = 7.2 Hz, 3H), 6.94 (t, *J* = 7.2 Hz, 1H), 6.70 (t, *J* = 7.2 Hz, 1H), 6.47 (d, *J* = 7.8 Hz, 1H), 5.88 (s, 1H, NCH), 1.43 (s, 9H, C(CH_3_)_3_), 1.03 (s, 3H, HfCH_3_), 0.80 (s, 3H, HfCH_3_) ppm. ^13^C{^1^H} NMR (150 MHz, C_6_D_6_): δ 31.10, 55.17, 60.66, 63.223, 75.73, 119.26, 119.42, 124.02, 125.22, 126.78, 127.30, 127.42, 129.15, 129.77, 129.97, 130.55, 134.49, 135.42 ppm. Anal. Calcd. (C_28_H_30_N_2_Hf): C, 58.69; H, 5.28; N, 4.89%. Found: C, 58.81; H, 5.38; N, 4.97%.

### 3.21. Complex ***25***

The complex was prepared by the same procedure and conditions as those employed for **3** using HfCl_4_ (0.256 g, 0.800 mmol), MeMgBr (1.10 mL, 3.28 mmol, 3.0 M solution in diethyl ether), and **21** (0.237 g, 0.533 mmol). A yellow solid was obtained (0.288 g, 83%). Single crystals suitable for X-ray crystallography were obtained by recrystallization in toluene/hexane cosolvent at −30 °C. ^1^H NMR (600 MHz, C_6_D_6_): δ 8.68 (d, *J* = 7.8 Hz, 1H), 8.17 (d, *J* = 8.4 Hz, 1H), 7.85 (d, *J* = 7.8 Hz, 1H), 7.72 (d, *J* = 8.4 Hz, 1H), 7.38 (d, *J* = 8.4 Hz, 2H), 7.34 (d, *J* = 8.4 Hz, 1H), 7.33–7.25 (m, 2H), 7.08 (t, *J* = 7.8 Hz, 2H), 6.94 (t, *J* = 7.8 Hz, 1H), 6.73 (t, *J* = 7.8 Hz, 1H), 6.54 (d, *J* = 7.8 Hz, 1H), 6.05 (s, 1H, NCH), 2.33 (d, 3H, *J* = 11.4 Hz, C_10_H_16_), 2.10 (d, 3H, *J* = 10.8 Hz, C_10_H_16_), 2.02 (s, 3H, C_10_H_16_), 1.62 (d, 3H, *J* = 12 Hz, C_10_H_16_), 1.56 (d, 3H, *J* = 11.4 Hz, C_10_H_16_), 1.10 (s, 3H, HfCH_3_), 0.86 (s, 3H, HfCH_3_) ppm. ^13^C{^1^H} NMR (150 MHz, C_6_D_6_): δ 30.30, 36.98, 44.25, 56.72, 61.04, 63.92, 73.91, 119.18, 119.44, 124.04, 125.20, 126.76, 127.16, 127.35, 127.98, 129.11, 129.73, 129.95, 130.55, 134.67, 135.42, 140.80, 143.10, 149.16, 164.91, 170.90, 204.57 ppm. Anal. Calcd. (C_34_H_36_N_2_Hf): C, 62.71; H, 5.57; N, 4.30%. Found: C, 62.59; H, 5.44; N, 4.18%.

### 3.22. Polymerization

A bomb reactor (125 mL) was evacuated at 60 °C for 1 h. After charging with ethylene gas at atmospheric pressure, a solution of Me_3_Al (28.8 mg, 200 µmol-Al) in methylcyclohexane (15.5 g) was added to the reactor. The mixture was stirred for 1 h at 100 °C using a mantle, and the solution was subsequently removed using a cannula. The reactor was evacuated once more to remove any residual solvent and was re-charged with ethylene gas at atmospheric pressure. This procedure was performed to clean up any catalyst poisons. The reactor was charged with methylcyclohexane (15.5 g), which contains MMAO (AkzoNobel, 6.7 wt%-Al in heptane, 20 mg, 50 µmol-Al) and the temperature was set to 80 °C. The methylcyclohexane solution (0.30 g) containing the catalyst (2.0 µmol-Hf) that was activated with [(C_18_H_37_)_2_N(H)Me]^+^[B(C_6_F_5_)_4_]^−^ (1.0 eq) in benzene, was injected. Ethylene/propylene mixed gas (10 bar/10 bar, total 20 bar) was charged from a tank into the reactor at 20 bar, and the polymerization was performed for 50 min. The temperature was controlled within the range of 80–90 °C. The remaining ethylene/propylene mixed gas was vented off and the reactor was cooled to 75 °C. The generated polymer was collected and dried in a vacuum oven at 160 °C overnight.

### 3.23. X-Ray Crystallography

Reflection data for **4**, **22**, and **25** were collected at 100 K on an APEX II CCD area diffractometer (Bruker) using graphite-monochromated Mo-Kα radiation (λ = 0.7107 Ǻ). Specimens of suitable quality and size were selected, mounted, and centered in the X-ray beam using a video camera. The hemisphere of the reflection data was collected as φ and ω scan frames at 0.5 ° per frame and an exposure time of 10 s per frame. The cell parameters were determined and refined by the SMART program. Data reduction was performed using SAINT software. The data were corrected for Lorentz and polarization effects. An empirical absorption correction was applied using the SADABS program. The structures of the compounds were solved by direct methods and refined by full matrix least-squares methods using the SHELXTL package with anisotropic thermal parameters for all non-hydrogen atoms. CCDC 1903714, 1903716, and 1903715 contain the supplementary crystallographic data for this paper. These data can be obtained free of charge via http://www.ccdc.cam.ac.uk/conts/retrieving.html (or from the CCDC, 12 Union Road, Cambridge CB2 1EZ, UK; Fax: +44 1223 336033; E-mail: deposit@ccdc.cam.ac.uk) Crystallographic data for **4** (CCDC 1903714): C_33_H_47_HfN_3_, *M* = 664.23, monoclinic, *a* = 14.1271(3), *b* = 20.7207(3), *c* = 20.3386(3) Å, β = 90.4281(8) ° *V* = 5953.42(18) Å^3^, *T* = 100(2) K, space group C2/*c*, *Z* = 8, 6117 unique (R(int) = 0.0152) which were used in all calculations. The final *wR*_2_ was 0.0385 (*I* >2σ(*I*)). Data for **22** (CCDC 1903716): C_27_H_28_HfN_2_, *M* = 559.00, monoclinic, *a* = 7.4314(3), *b* = 17.1814(7), *c* = 35.3369(13) Å, β = 90.1529(18) °, *V* = 4511.9(3) Å^3^, *T* = 100(2) K, space group *P*2_1_/*_C_*, *Z* = 8, 8305 unique (R(int) = 0.0805) which were used in all calculations. The final *wR*_2_ was 0.1700 (*I* > 2σ(*I*)). Data for **25** (CCDC 1903715): C_34_H_36_HfN_2_, *M* = 651.14, triclinic, *a* = 9.1883(6), *b* = 10.9598(7), *c* = 13.8783(9) Å, α = 75.009(4), β = 83.871(3), γ = 89.949(4) °, *V* = 1341.77(15) Å^3^, *T* = 100(2) K, space group *P*-1, *Z* = 2, 4965 unique (R(int) = 0.0514) which were used in all calculations. The final *wR*_2_ was 0.0696 (*I* >2σ(*I*)).

## 4. Conclusions

Various pincer-type Hf-complexes were prepared mimicking the prototype [C^naphthyl^, N^pyridine^, N^arylamido^]HfMe_2_ complex **I**. [N^arylamido^, N^pyridine^, N^arylamido^]HfMe_2_ and [C^silylmethyl^, N^pyridine^, N^arylamido^]HfMe_2_ pincer complexes, along with a series of [C^naphthyl^, N^pyridine^, N^alkylamido^]HfMe_2_ complexes where the arylamido moiety in the prototype complex was replaced with alkylamido moieties (alkyl = iPr, cyclohexyl, *t*Bu, and adamantyl), were successfully prepared by the treatment of the corresponding ligand precursors with in situ generated HfMe_4_. In the case of analogous ligand precursor where the pyridine moiety was replaced with thiophene, the Hf-C^naphthyl^ bond was not formed and the [S, N^amido^]HfMe_3_-type complex was generated instead. Most of the prepared complexes cleanly generated the desired ion-pair complex when treated with [(C_18_H_37_)_2_N(H)Me]^+^[B(C_6_F_5_)_4_]^−^. However, the generated ion-pair complexes were inactive in ethylene/propylene copolymerization. Several complexes bearing the bulky iPr_3_Si- or adamantyl substituent exhibited moderate activities (approximately 1/10th that of **I**), although the ^1^H NMR spectra were not clean in the activation reaction with [(C_18_H_37_)_2_N(H)Me]^+^[B(C_6_F_5_)_4_]^−^.

## Figures and Tables

**Table 1:** Polymerization results ^a^.

Entry	Complex	Yield (g)	[C_3_H_6_] ^b^ (mol%)	*T*_m_	*M*_n_ (kDa) ^c^	*M*_w_/*M*_n_
1	**4**	2.3	6.7	113–125	10	2.9
2	**13**	1.2	0	119–139	430	2.3
3	**25**	1.7	5.1	91–111	45	1.6
4	**I** (Dow)	16	56	not detected	61	2.6

^a^ Polymerization conditions: Hf complex (2.0 μmol), activator ([(C_18_H_37_)_2_N(H)Me]^+^[B(C_6_F_5_)_4_]^−^, 2.0 μmol), scavenger (MMAO, 50 μmol), methylcyclohexane (26 g), ethylene and propylene mixed gas (1:1, 20 bar), 80 °C, 50 min. ^b^ Propylene content measured by ^1^H-NMR spectra. ^c^ Measured by gel permeation chromatography (GPC) at 160 °C using trichlorobenzene and calculated relative to polystyrene (PS) standards.

## Supplementary Materials

The following are available online, Figures S1–S20: ^1^H and ^13^C NMR spectra of **3**, **4**, **6**, **8**, **9**, **10**, **11**, **12**, **13**, **14**, **15**, **16**, **18**, **19**, **20**, **21**, **22**, **23**, **24**, and **25**, Figures S21–S29: ^1^H NMR spectra of the complexes activated with [(C_18_H_37_)_2_N(H)Me]^+^[B(C_6_F_5_)_4_]^−^.

## References

[1] Wang Z., Solan G.A., Zhang W., Sun W.-H. (2018). Carbocyclic-fused N,N,N-pincer ligands as ring-strain adjustable supports for iron and cobalt catalysts in ethylene oligo-/polymerization. Coord. Chem. Rev..

[2] Valdés H., García-Eleno M.A., Canseco-Gonzalez D., Morales-Morales D. (2018). Recent Advances in Catalysis with Transition-Metal Pincer Compounds. ChemCatChem.

[3] Chen C. (2018). Redox-Controlled Polymerization and Copolymerization. ACS Catal..

[4] Tan C., Chen C. (2018). Emerging Palladium and Nickel Catalysts for Copolymerization of Olefins with Polar Monomers. Angew. Chem. Int. Ed..

[5] Ito S. (2018). Palladium-Catalyzed Homo- and Copolymerization of Polar Monomers: Synthesis of Aliphatic and Aromatic Polymers. Bull. Chem. Soc. Jpn..

[6] Kaminsky W. (2012). Discovery of Methylaluminoxane as Cocatalyst for Olefin Polymerization. Macromolecules.

[7] Lee S., Park S.S., Kim J.G., Kim C.S., Lee B.Y. (2017). Preparation of “Constrained geometry” titanium complexes of [1,2]azasilinane framework for ethylene/1-octene copolymerization. Molecules.

[8] Kim S.H., Park J.H., Song B.G., Yoon S.W., Go M.J., Lee J., Lee B.Y. (2013). Preparation of thiophene-fused and tetrahydroquinoline-linked cyclopentadienyl titanium complexes for ethylene/α-olefin copolymerization. Catalysts.

[9] Makio H., Terao H., Iwashita A., Fujita T. (2011). FI catalysts for olefin polymerization—A comprehensive treatment. Chem. Rev..

[10] Arriola D.J., Carnahan E.M., Hustad P.D., Kuhlman R.L., Wenzel T.T. (2006). Catalytic production of olefin block copolymers via chain shuttling polymerization. Science.

[11] Boussie T.R., Diamond G.M., Goh C., Hall K.A., LaPointe A.M., Leclerc M.K., Murphy V., Shoemaker J.A.W., Turner H., Rosen R.K. (2006). Nonconventional Catalysts for Isotactic Propene Polymerization in Solution Developed by Using High-Throughput-Screening Technologies. Angew. Chem. Int. Ed..

[12] Frazier K.A., Froese R.D., He Y., Klosin J., Theriault C.N., Vosejpka P.C., Zhou Z., Abboud K.A. (2011). Pyridylamido hafnium and zirconium complexes: Synthesis, dynamic behavior, and ethylene/1-octene and propylene polymerization reactions. Organometallics.

[13] Alfano F., Boone H.W., Busico V., Cipullo R., Stevens J.C. (2007). Polypropylene “Chain Shuttling” at Enantiomorphous and Enantiopure Catalytic Species:  Direct and Quantitative Evidence from Polymer Microstructure. Macromolecules.

[14] Domski G.J., Eagan J.M., De Rosa C., Di Girolamo R., LaPointe A.M., Lobkovsky E.B., Talarico G., Coates G.W. (2017). Combined Experimental and Theoretical Approach for Living and Isoselective Propylene Polymerization. ACS Catal..

[15] De Rosa C., Di Girolamo R., Talarico G. (2016). Expanding the Origin of Stereocontrol in Propene Polymerization Catalysis. ACS Catal..

[16] Park S.S., Kim C.S., Kim S.D., Kwon S.J., Lee H.M., Kim T.J., Jeon J.Y., Lee B.Y. (2017). Biaxial Chain Growth of Polyolefin and Polystyrene from 1,6-Hexanediylzinc Species for Triblock Copolymers. Macromolecules.

[17] Valente A., Mortreux A., Visseaux M., Zinck P. (2013). Coordinative chain transfer polymerization. Chem. Rev..

[18] Van Meurs M., Britovsek G.J.P., Gibson V.C., Cohen S.A. (2005). Polyethylene Chain Growth on Zinc Catalyzed by Olefin Polymerization Catalysts:  A Comparative Investigation of Highly Active Catalyst Systems across the Transition Series. J. Am. Chem. Soc..

[19] Rocchigiani L., Busico V., Pastore A., Macchioni A. (2016). Comparative NMR Study on the Reactions of Hf(IV) Organometallic Complexes with Al/Zn Alkyls. Organometallics.

[20] Hustad P.O., Kuhlman R.L., Arriola D.J., Carnahan E.M., Wenzel T.T. (2007). Continuous production of ethylene-based diblock copolymers using coordinative chain transfer polymerization. Macromolecules.

[21] Kim S.D., Kim T.J., Kwon S.J., Kim T.H., Baek J.W., Park H.S., Lee H.J., Lee B.Y. (2018). Peroxide-Mediated Alkyl–Alkyl Coupling of Dialkylzinc: A Useful Tool for Synthesis of ABA-Type Olefin Triblock Copolymers. Macromolecules.

[22] Vittoria A., Busico V., Cannavacciuolo F.D., Cipullo R. (2018). Molecular Kinetic Study of “Chain Shuttling” Olefin Copolymerization. ACS Catal..

[23] Jeon J.Y., Park S.H., Kim D.H., Park S.S., Park G.H., Lee B.Y. (2016). Synthesis of polyolefin-block-polystyrene through sequential coordination and anionic polymerizations. J. Polym. Sci. Part A Polym. Chem..

[24] Kim C.S., Park S.S., Kim S.D., Kwon S.J., Baek J.W., Lee B.Y. (2017). Polystyrene chain growth from di-end-functional polyolefins for polystyrene-polyolefin-polystyrene block copolymers. Polymers.

[25] Kim D.H., Park S.S., Park S.H., Jeon J.Y., Kim H.B., Lee B.Y. (2017). Preparation of polystyrene-polyolefin multiblock copolymers by sequential coordination and anionic polymerization. RSC Adv..

[26] Liu C.-C., Liu Q., Lo P.-K., Lau K.-C., Yiu S.-M., Chan M.C.W. (2019). Olefin Polymerization Reactivity of Group 4 Post-Metallocene Catalysts Bearing a Four-Membered C(sp3)-Donor Chelate Ring. ChemCatChem.

[27] Li G., Lamberti M., D’Amora S., Pellecchia C. (2010). C1-Symmetric Pentacoordinate Anilidopyridylpyrrolide Zirconium(IV) Complexes as Highly Isospecific Olefin Polymerization Catalysts. Macromolecules.

[28] Matsumoto K., Takayanagi M., Suzuki Y., Koga N., Nagaoka M. (2019). Atomistic chemical computation of Olefin polymerization reaction catalyzed by (pyridylamido)hafnium(IV) complex: Application of Red Moon simulation. J. Comput. Chem..

[29] Schnee G., Farenc M., Bitard L., Vantomme A., Welle A., Brusson J.M., Afonso C., Giusti P., Carpentier J.F., Kirillov E. (2018). Synthesis APPI mass-spectrometric characterization, and polymerization studies of group 4 dinuclear bis(Ansa-metallocene) complexes. Catalysts.

[30] Xu J., Eagan J.M., Kim S.-S., Pan S., Lee B., Klimovica K., Jin K., Lin T.-W., Howard M.J., Ellison C.J. (2018). Compatibilization of Isotactic Polypropylene (iPP) and High-Density Polyethylene (HDPE) with iPP–PE Multiblock Copolymers. Macromolecules.

[31] Zhang J., Motta A., Gao Y., Stalzer M.M., Delferro M., Liu B., Lohr T.L., Marks T.J. (2018). Cationic Pyridylamido Adsorbate on Brønsted Acidic Sulfated Zirconia: A Molecular Supported Organohafnium Catalyst for Olefin Homo- and Co-Polymerization. ACS Catal..

[32] Cueny E.S., Johnson H.C., Landis C.R. (2018). Selective Quench-Labeling of the Hafnium-Pyridyl Amido-Catalyzed Polymerization of 1-Octene in the Presence of Trialkyl-Aluminum Chain-Transfer Reagents. ACS Catal..

[33] Johnson H.C., Cueny E.S., Landis C.R. (2018). Chain Transfer with Dialkyl Zinc during Hafnium-Pyridyl Amido-Catalyzed Polymerization of 1-Octene: Relative Rates, Reversibility, and Kinetic Models. ACS Catal..

[34] Gao Y., Chen X., Zhang J., Chen J., Lohr T.L., Marks T.J. (2018). Catalyst Nuclearity Effects on Stereo- and Regioinduction in Pyridylamidohafnium-Catalyzed Propylene and 1-Octene Polymerizations. Macromolecules.

[35] Matsumoto K., Takayanagi M., Sankaran S.K., Koga N., Nagaoka M. (2018). Role of the Counteranion in the Reaction Mechanism of Propylene Polymerization Catalyzed by a (Pyridylamido)hafnium(IV) Complex. Organometallics.

[36] Tay B.-Y., Wang C., Chia S.-C., Stubbs L.P., Wong P.-K., van Meurs M. (2011). Synthesis of Bis(amino)pyridines by the Stepwise Alkylation of Bis(imino)pyridines: An Unexpected and Selective Alkylation of the Aminoiminopyridine by AlMe_3_. Organometallics.

[37] Zhang C., Pan H., Klosin J., Tu S., Jaganathan A., Fontaine P.P. (2015). Synthetic Optimization and Scale-Up of Imino–Amido Hafnium and Zirconium Olefin Polymerization Catalysts. Org. Proc. Res. Dev..

[38] Corey E.J., Zheng G.Z. (1998). 2,6-Bis(triisopropylsilyl)pyridine, an extreme example of the effect of strong steric screening on basicity. Tetrahedron Lett..

[39] Bull S.D., Davies S.G., Fox D.J., Gianotti M., Kelly P.M., Pierres C., Savory E.D., Smith A.D. (2002). Asymmetric synthesis of β-pyridyl-β-amino acid derivatives. J. Chem. Soc. Perkin Trans. 1.

[40] Boussie T.R., Diamond G.M., Goh C., Hall K.A., La Pointe A.M., Leclerc M., Lund C., Murphy V. (2004). Substituted Pyridyl Amine Catalysts and Processes for Polymerization and Polymers. U.S. Patent.

[41] Frazier K.A., Boone H., Vosejpka P.C., Stevens J.C. (2005). High Activity Olefin Polymerization Catalyst and Process. U.S. Patent.

[42] Prajapati D., Schulzke C., Kindermann M.K., Kapdi A.R. (2015). Selective palladium-catalysed arylation of 2,6-dibromopyridine using N-heterocyclic carbene ligands. RSC Adv..

[43] Cueny E.S., Johnson H.C., Anding B.J., Landis C.R. (2017). Mechanistic Studies of Hafnium-Pyridyl Amido-Catalyzed 1-Octene Polymerization and Chain Transfer Using Quench-Labeling Methods. J. Am. Chem. Soc..

[44] Zuccaccia C., Macchioni A., Busico V., Cipullo R., Talarico G., Alfano F., Boone H.W., Frazier K.A., Hustad P.D., Stevens J.C. (2008). Intra- and Intermolecular NMR Studies on the Activation of Arylcyclometallated Hafnium Pyridyl-Amido Olefin Polymerization Precatalysts. J. Am. Chem. Soc..

[45] Jun S.H., Park J.H., Lee C.S., Park S.Y., Go M.J., Lee J., Lee B.Y. (2013). Preparation of phosphine-amido hafnium and zirconium complexes for olefin polymerization. Organometallics.

[46] Lee C.S., Park J.H., Hwang E.Y., Park G.H., Go M.J., Lee J., Lee B.Y. (2014). Preparation of [Bis(amido)-phosphine] and [Amido-Phosphine Sulfide or Oxide] Hafnium and Zirconium Complexes for Olefin Polymerization. J. Organom. Chem..

[47] Hwang E.Y., Park G.H., Lee C.S., Kang Y.Y., Lee J., Lee B.Y. (2015). Preparation of octahydro- and tetrahydro-[1,10]phenanthroline zirconium and hafnium complexes for olefin polymerization. Dalton Trans..

[48] Machat M.R., Fischer A., Schmitz D., Vöst M., Drees M., Jandl C., Pöthig A., Casati N.P.M., Scherer W., Rieger B. (2018). Behind the Scenes of Group 4 Metallocene Catalysis: Examination of the Metal–Carbon Bond. Organometallics.

[49] Lawson J.R., Wilkins L.C., Melen R.L. (2017). Tris(2,4,6-trifluorophenyl)borane: An Efficient Hydroboration Catalyst. Chem. Eur. J..

