# Supplementary material for: Preparation of Pincer Hafnium Complexes for Olefin Polymerization
Source: Molecules. 2019 Apr 29;24(9):1676. doi: 10.3390/molecules24091676 (PMC6540127; doi:10.3390/molecules24091676)

Supporting Information

Preparation of Pincer Hafnium Complexes for Olefin Polymerization

Su Jin Kwon [1], Jun Won Baek [1], Hyun Ju Lee [1], Tae Jin Kim [1], Ji Yeon Ryu [2], Junseong Lee [2], Eun Ji Shin [3], Ki Soo Lee [3] and Bun Yeoul Lee [1,*]

[1] Department of Molecular Science and Technology, Ajou University, Suwon 16499, Korea; ksj9355@ajou.ac.kr (S.J.K.); btw91@ajou.ac.kr (J.W.B.); hjulee4639@ajou.ac.kr (H.J.L.); playing3457@ajou.ac.kr (T.J.K.)

[2] Department of Chemistry, Chonnam National University, 77 Yongbong-ro, Buk-gu, Gwangju 500-757, Korea; jy5330@naver.com (J.Y.R.); leespy@chonnam.ac.kr (J.L.)

[3] LG Chem, Ltd., 188, Munji-ro, Yuseong-gu Daejeon 305-738, South Korea; eunjis@lgchem.com (E.J.S.); leekisoo@lgchem.com (K.S.L.)

* Correspondence: bunyeoul@ajou.ac.kr; Tel.: +82-31-219-1844

Figure S1. ^{1}H and ^{13}C NMR spectra of **3**

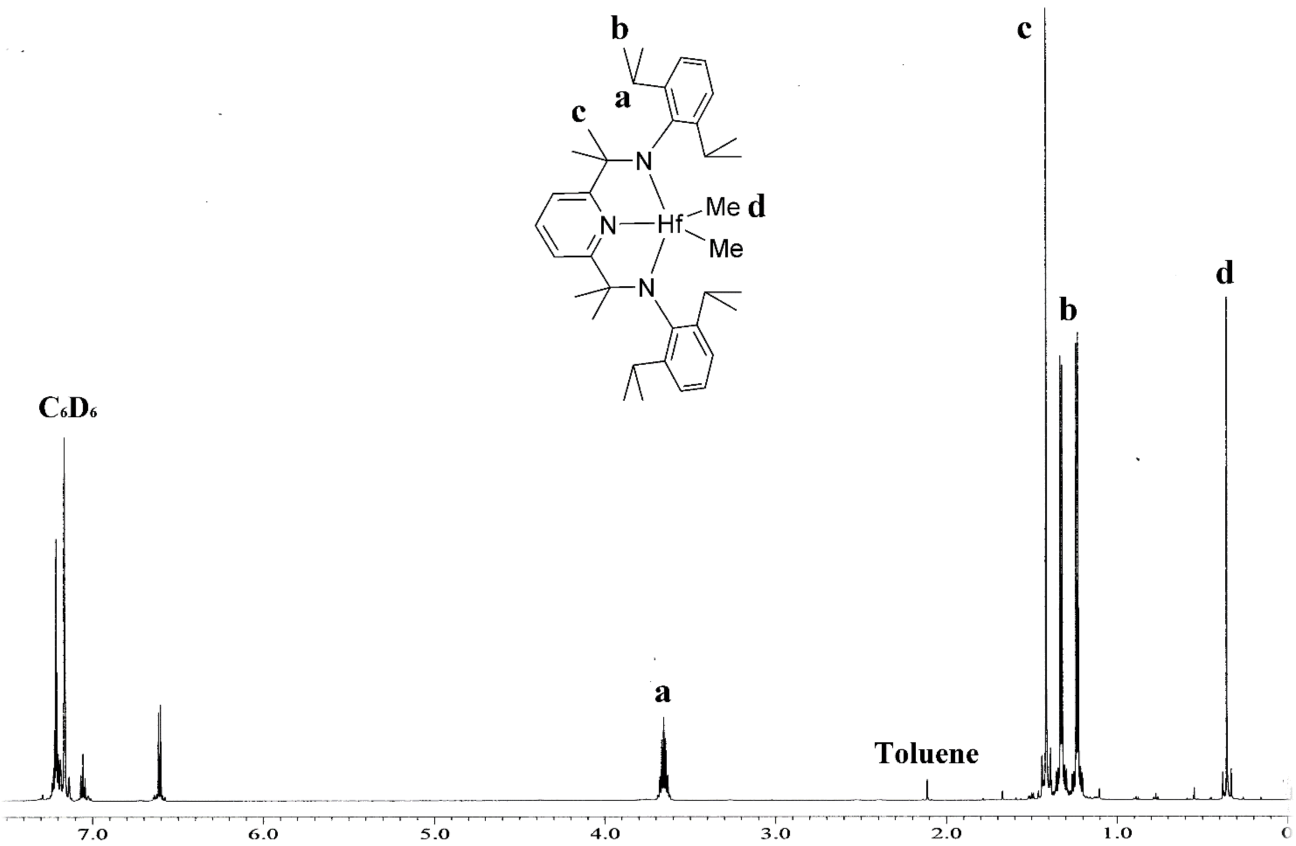

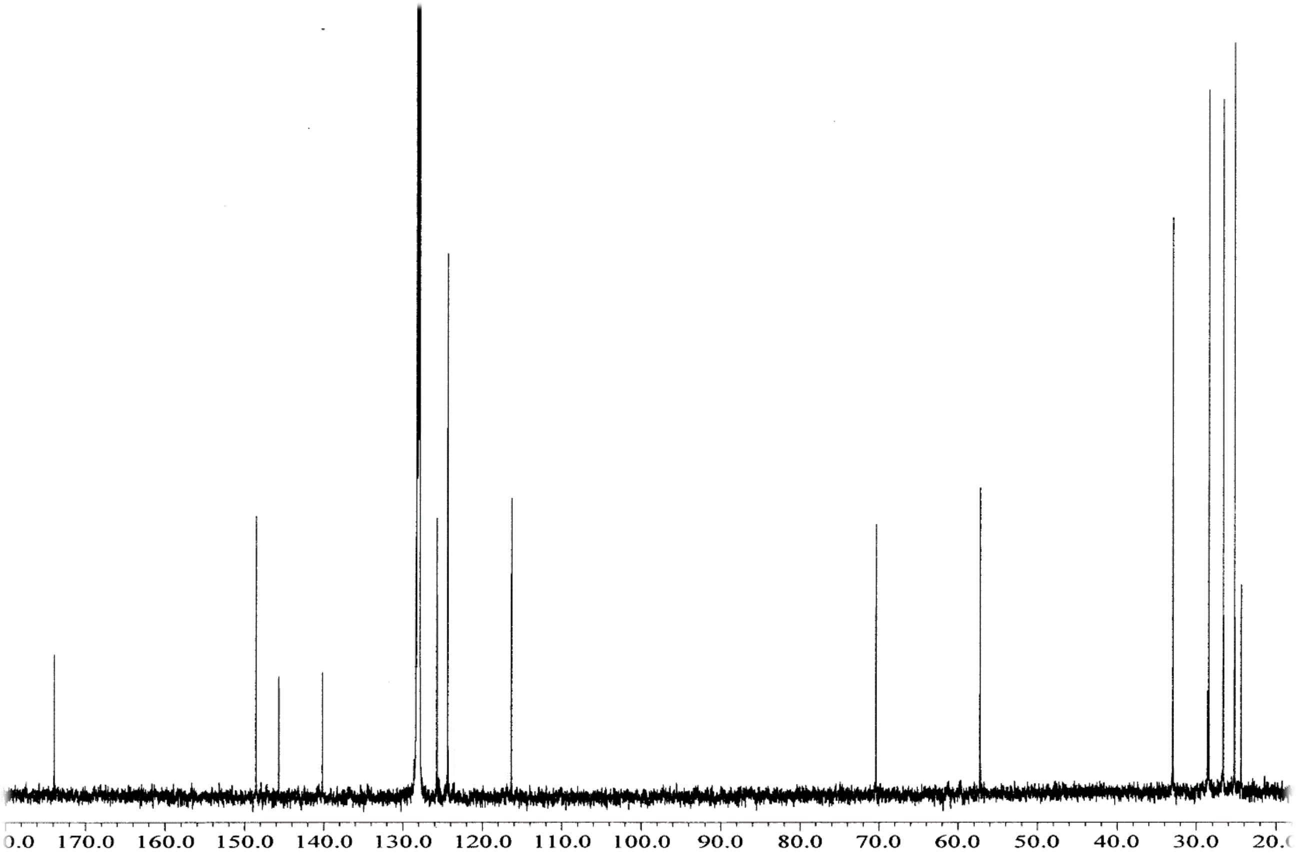

Figure S2. ^{1}H and ^{13}C NMR spectra of **4**

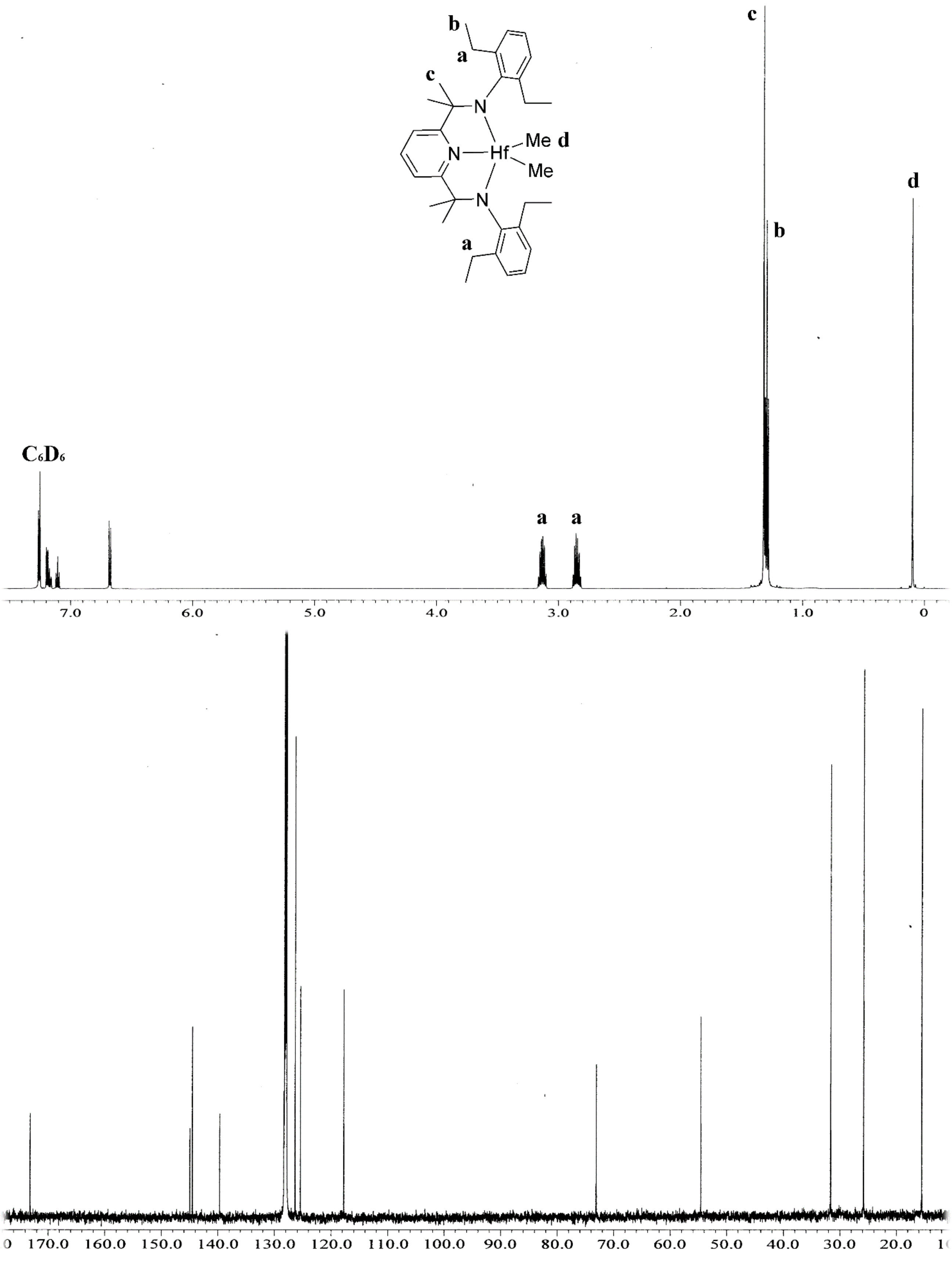

Figure S3. ^{1}H and ^{13}C NMR spectra of **6**

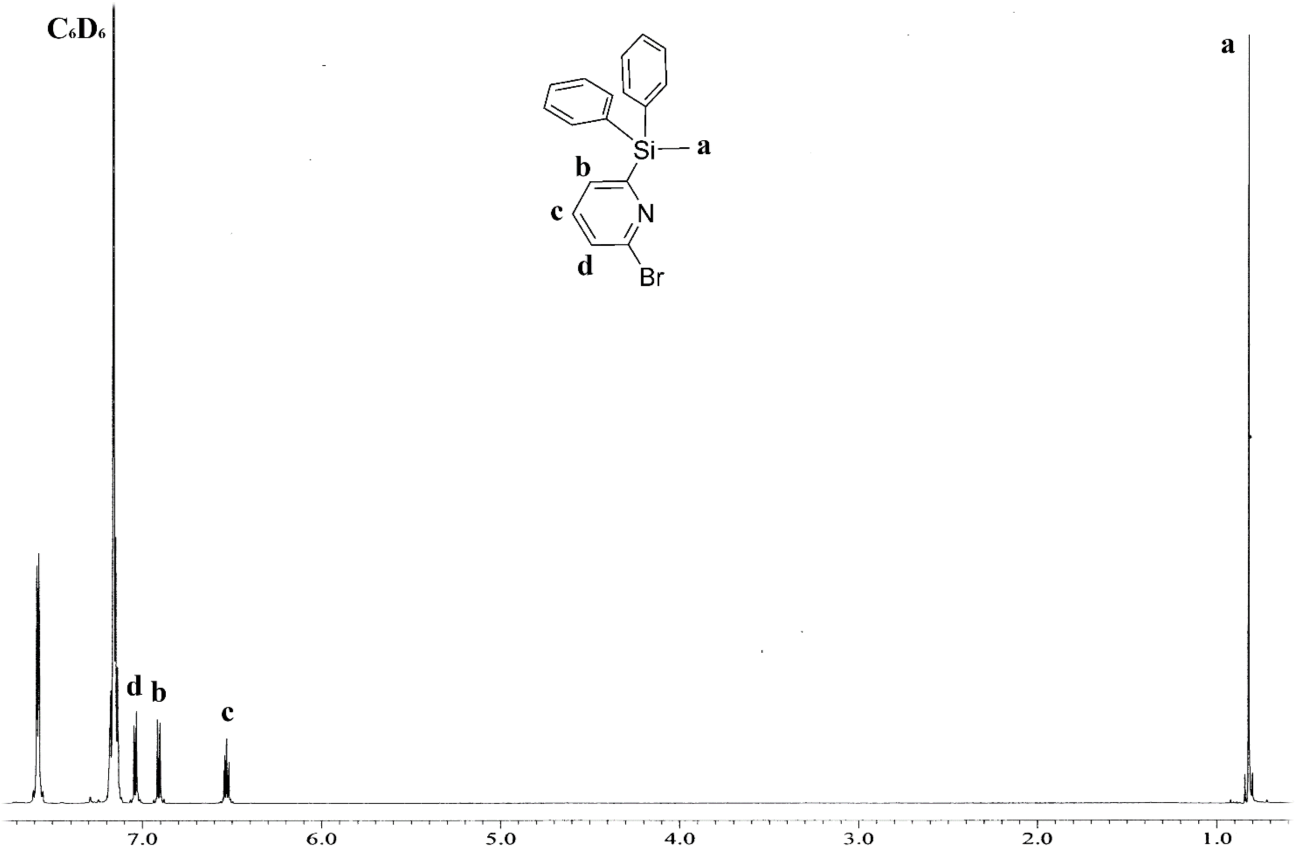

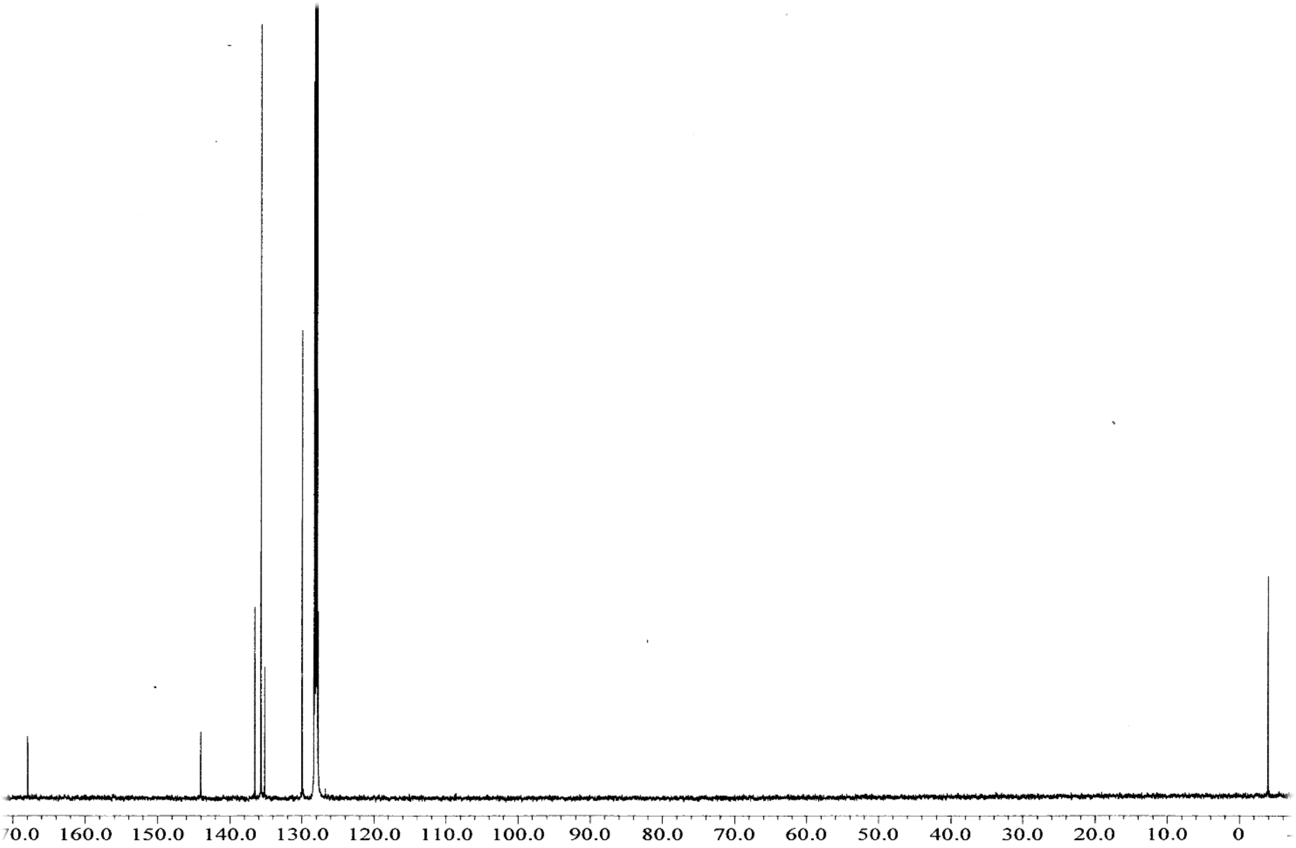

Figure S4. ^{1}H and ^{13}C NMR spectra of **8**

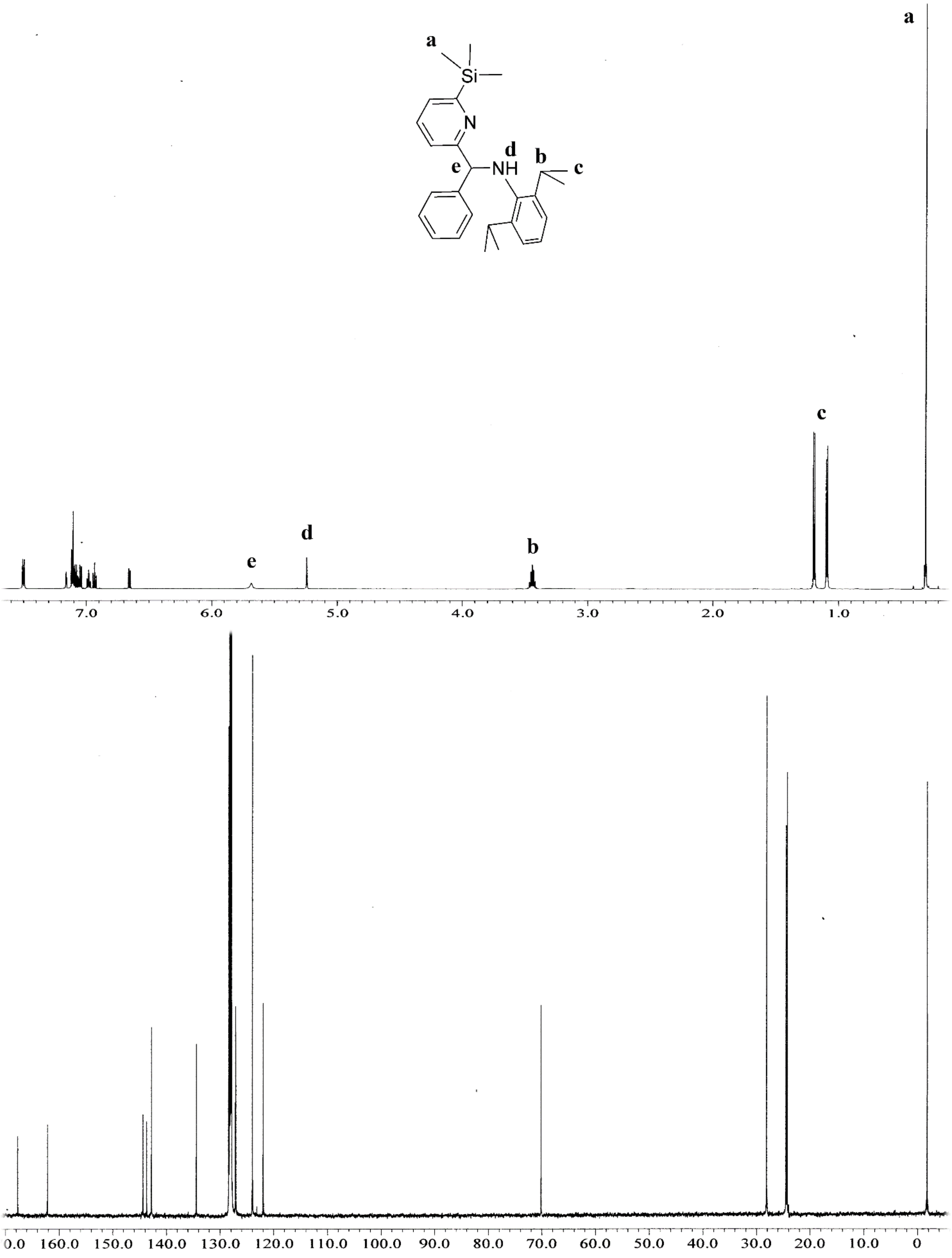

Figure S5. ^{1}H and ^{13}C NMR spectra of **9**

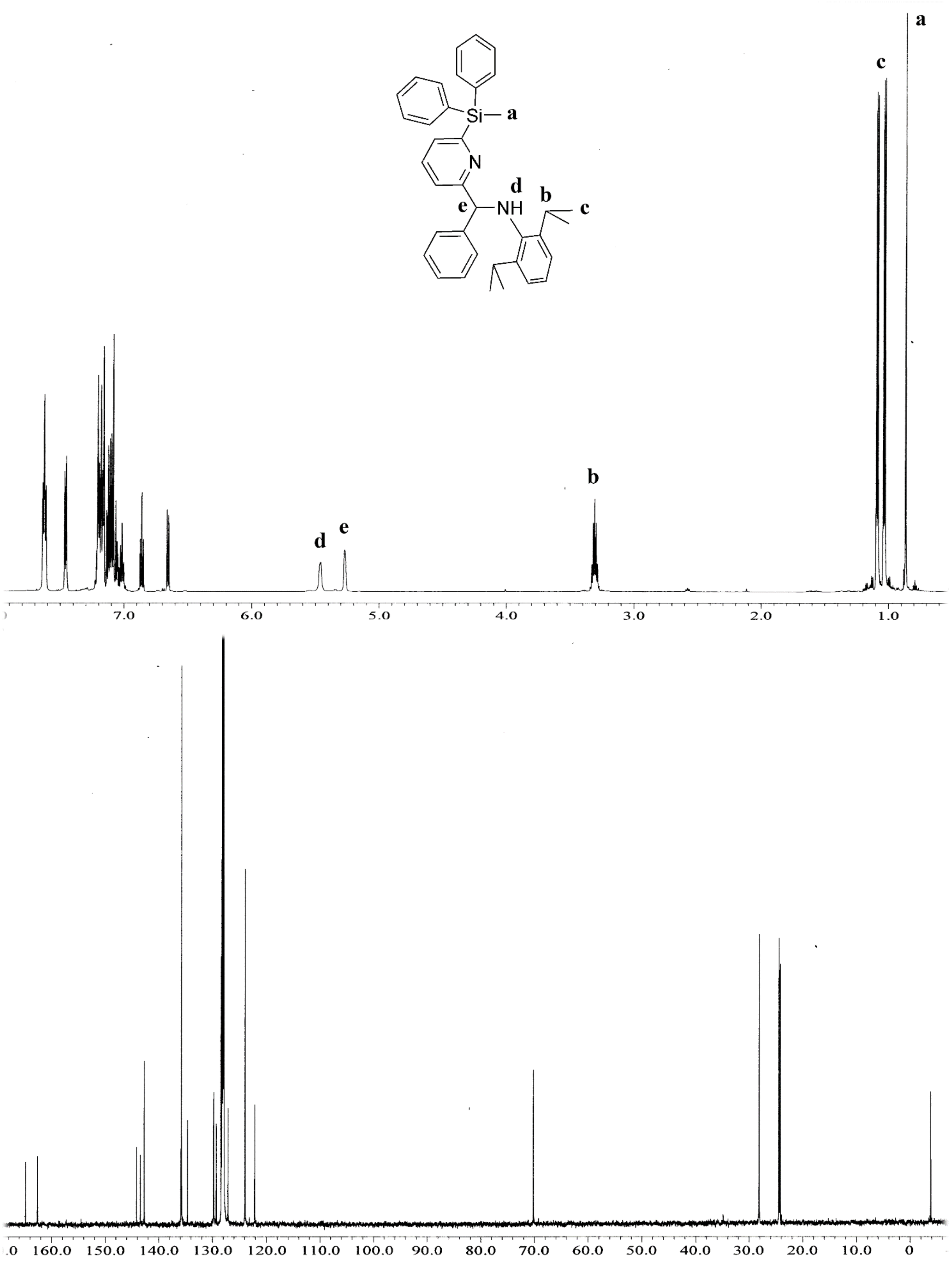

Figure S6. ^1H and ^{13}C NMR spectra of **10**

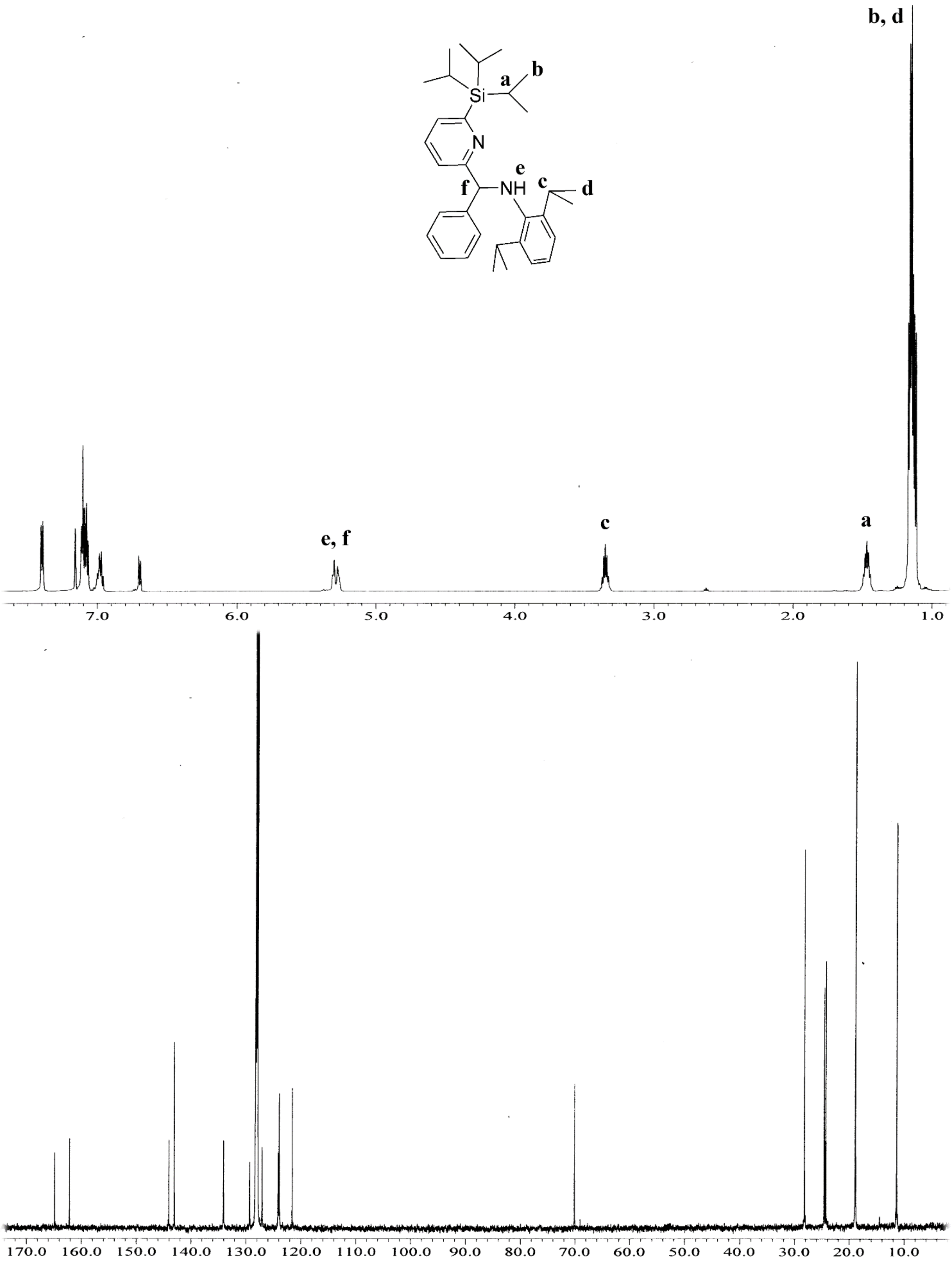

Figure S7. ^{1}H and ^{13}C NMR spectra of **11**

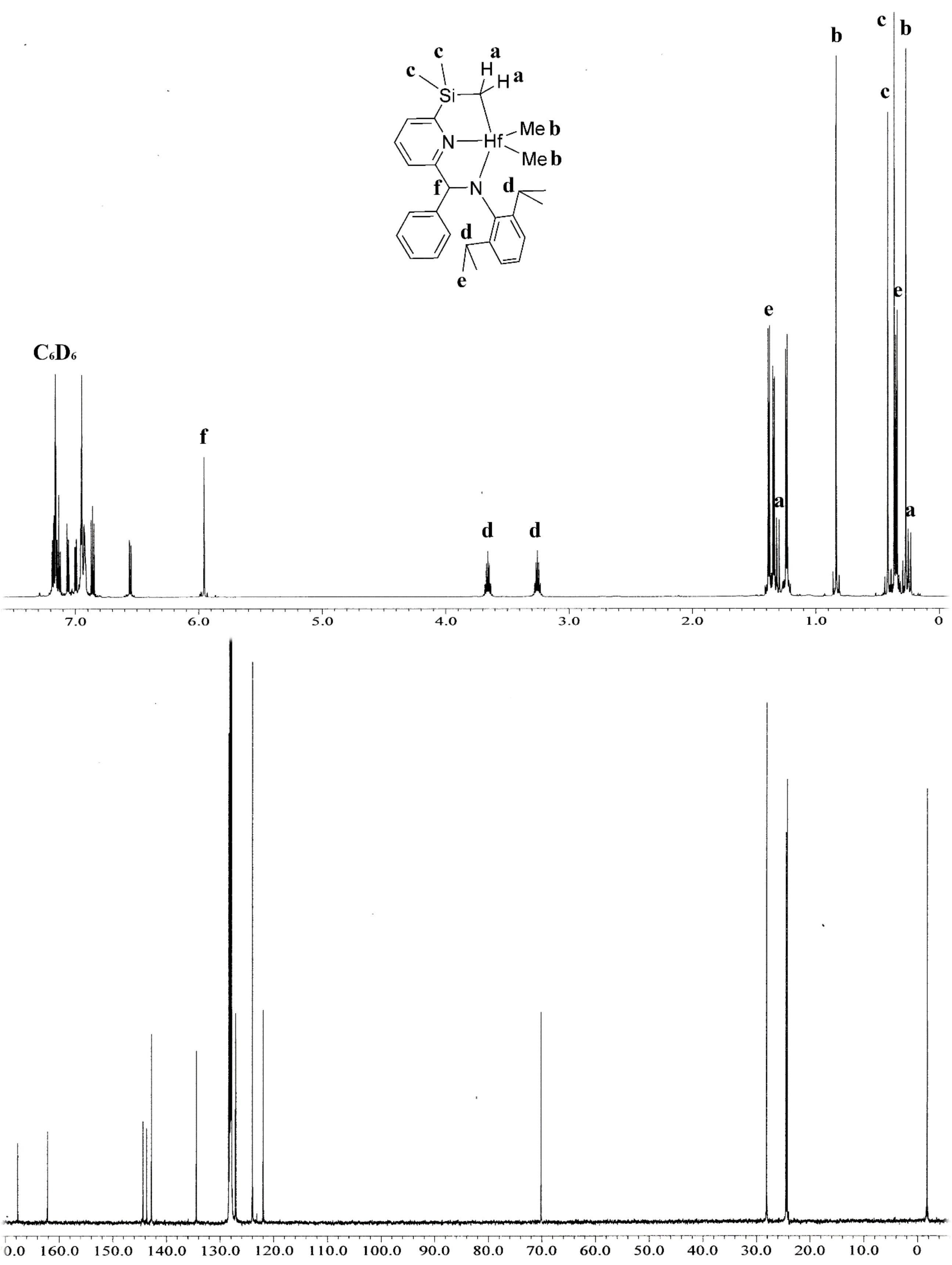

Figure S8. ^{1}H and ^{13}C NMR spectra of **12**

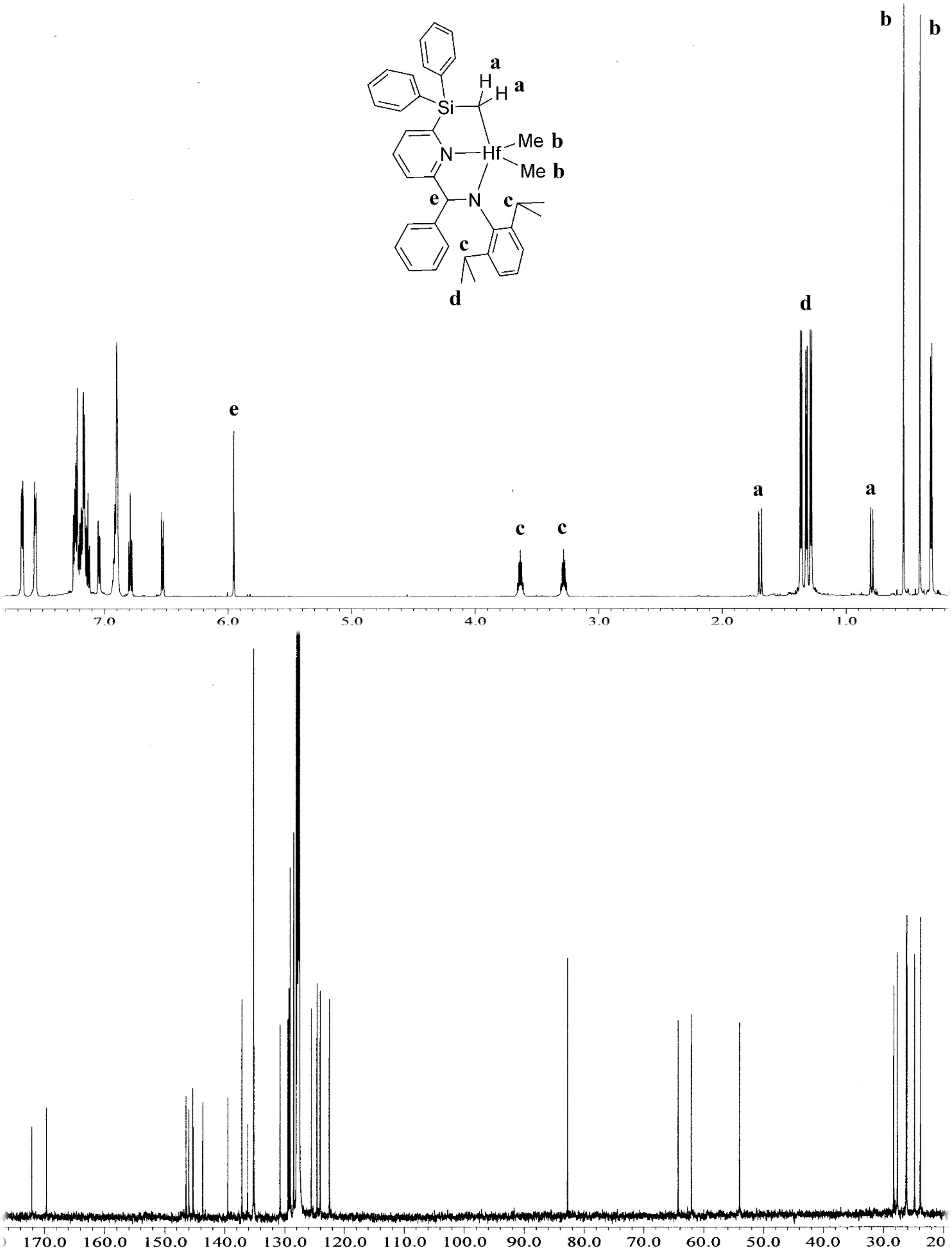

Figure S9. ^{1}H and ^{13}C NMR spectra of **13**

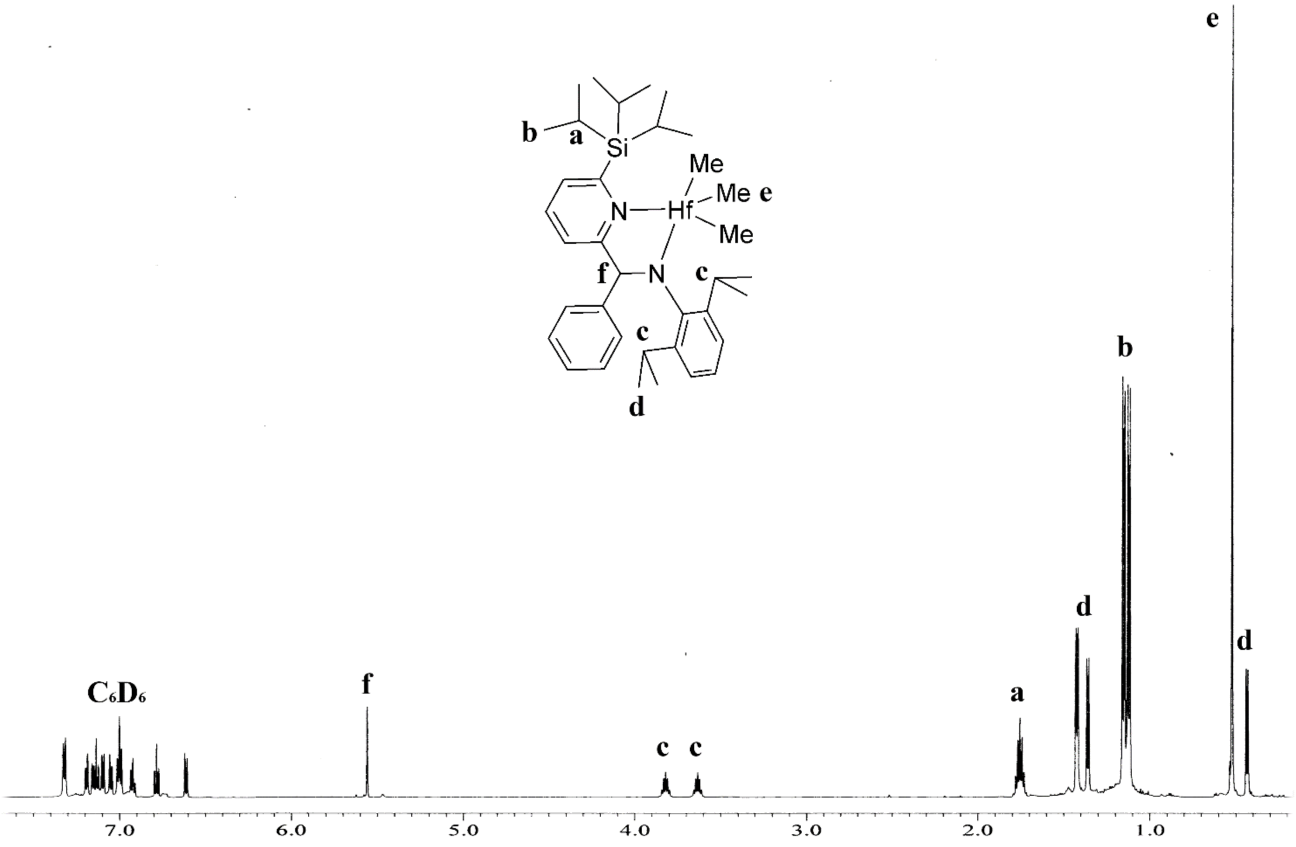

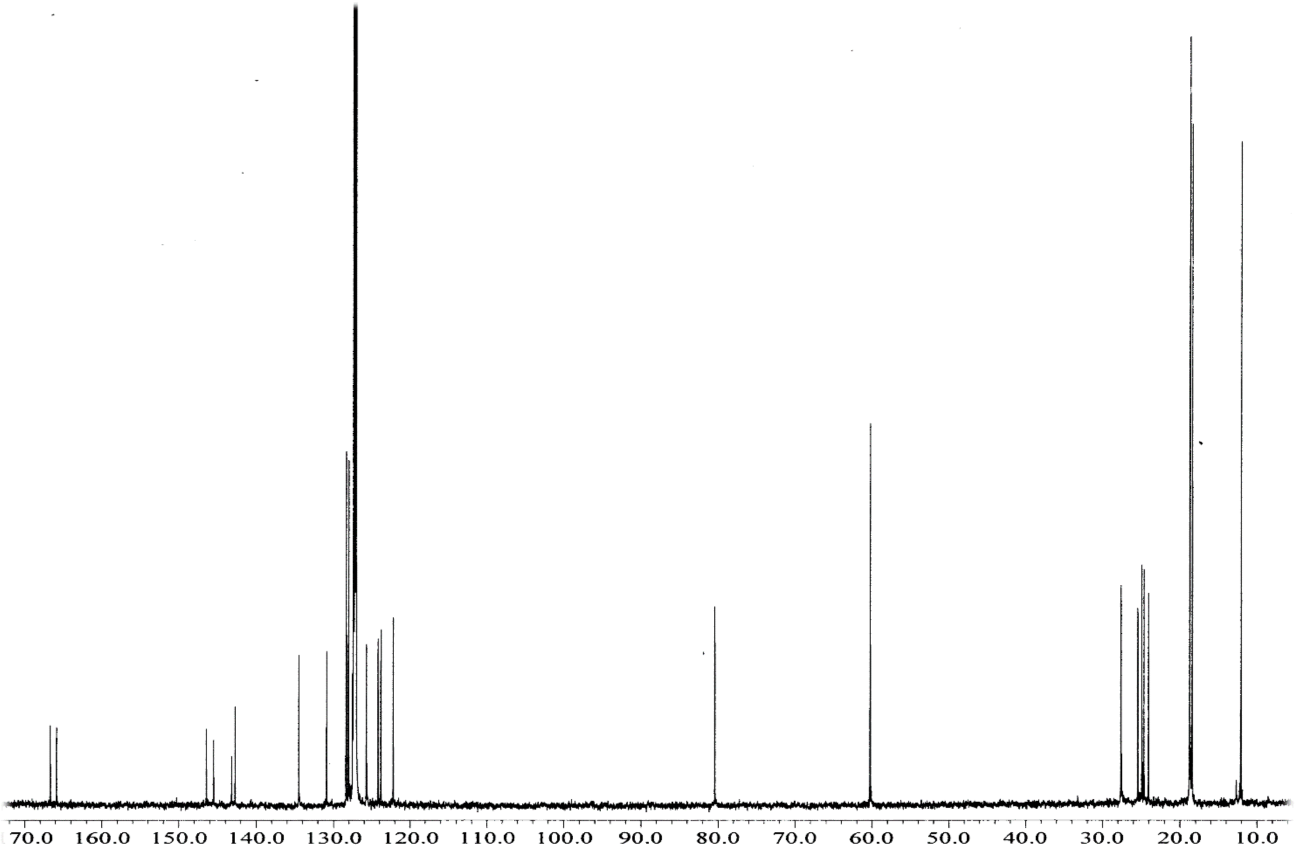

Figure S10. ^{1}H and ^{13}C NMR spectra of **14**

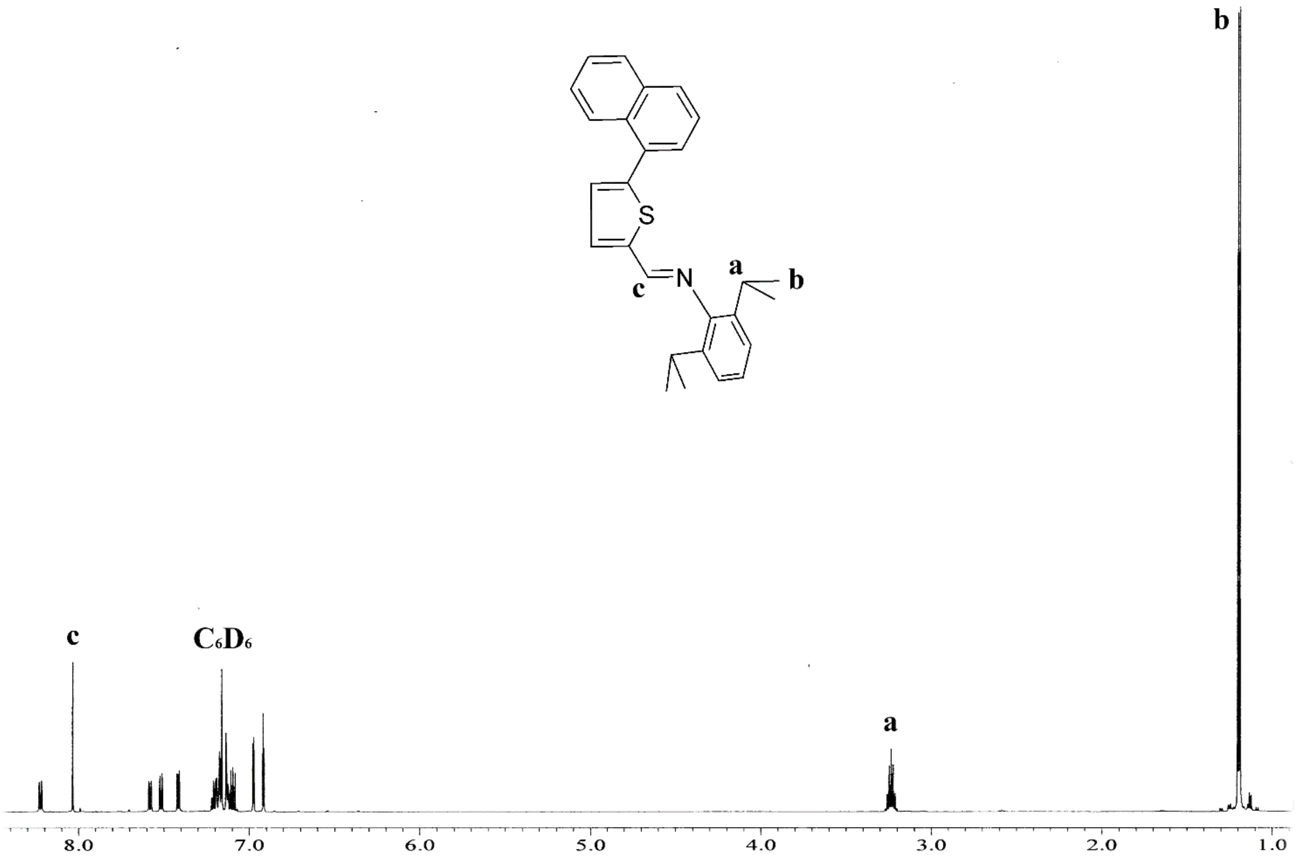

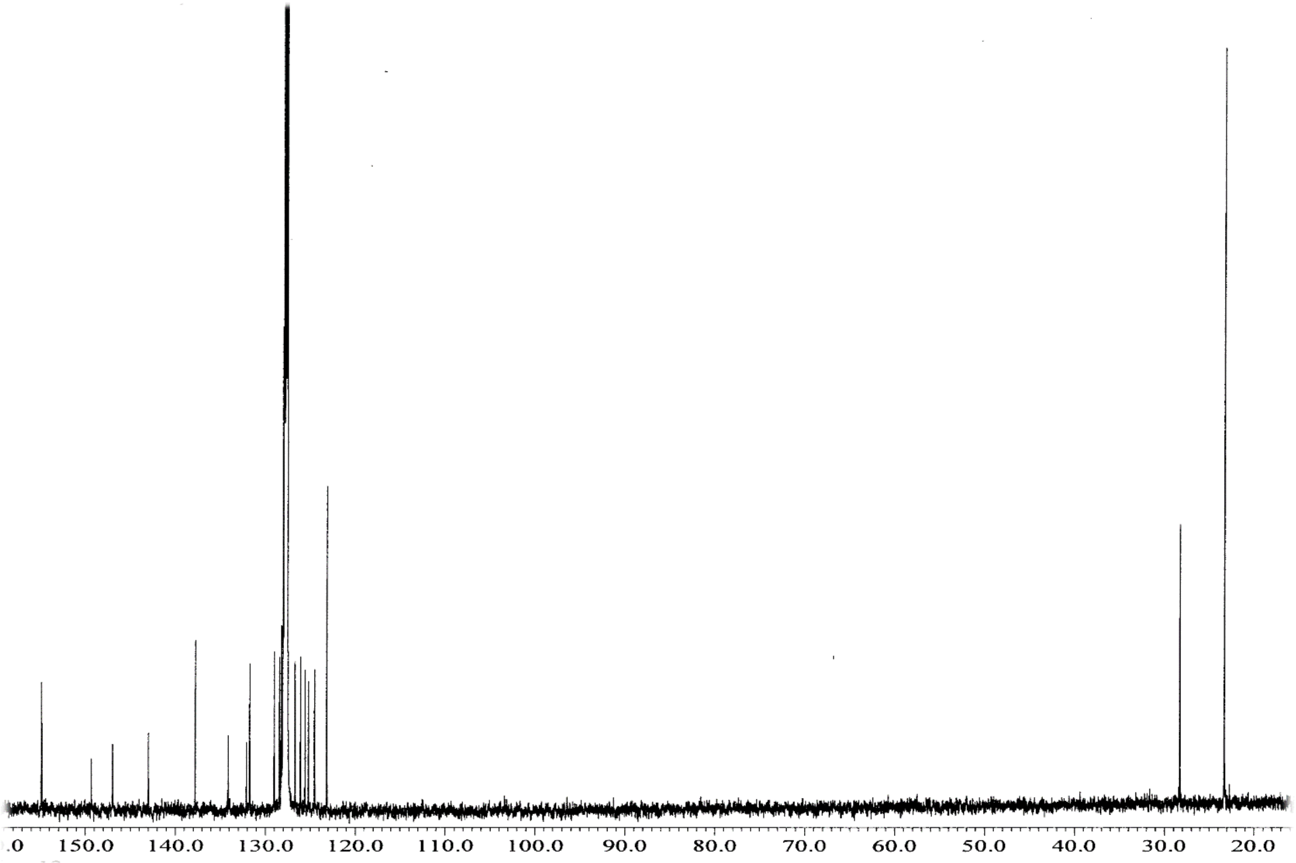

Figure S11. ^{1}H and ^{13}C NMR spectra of **15**

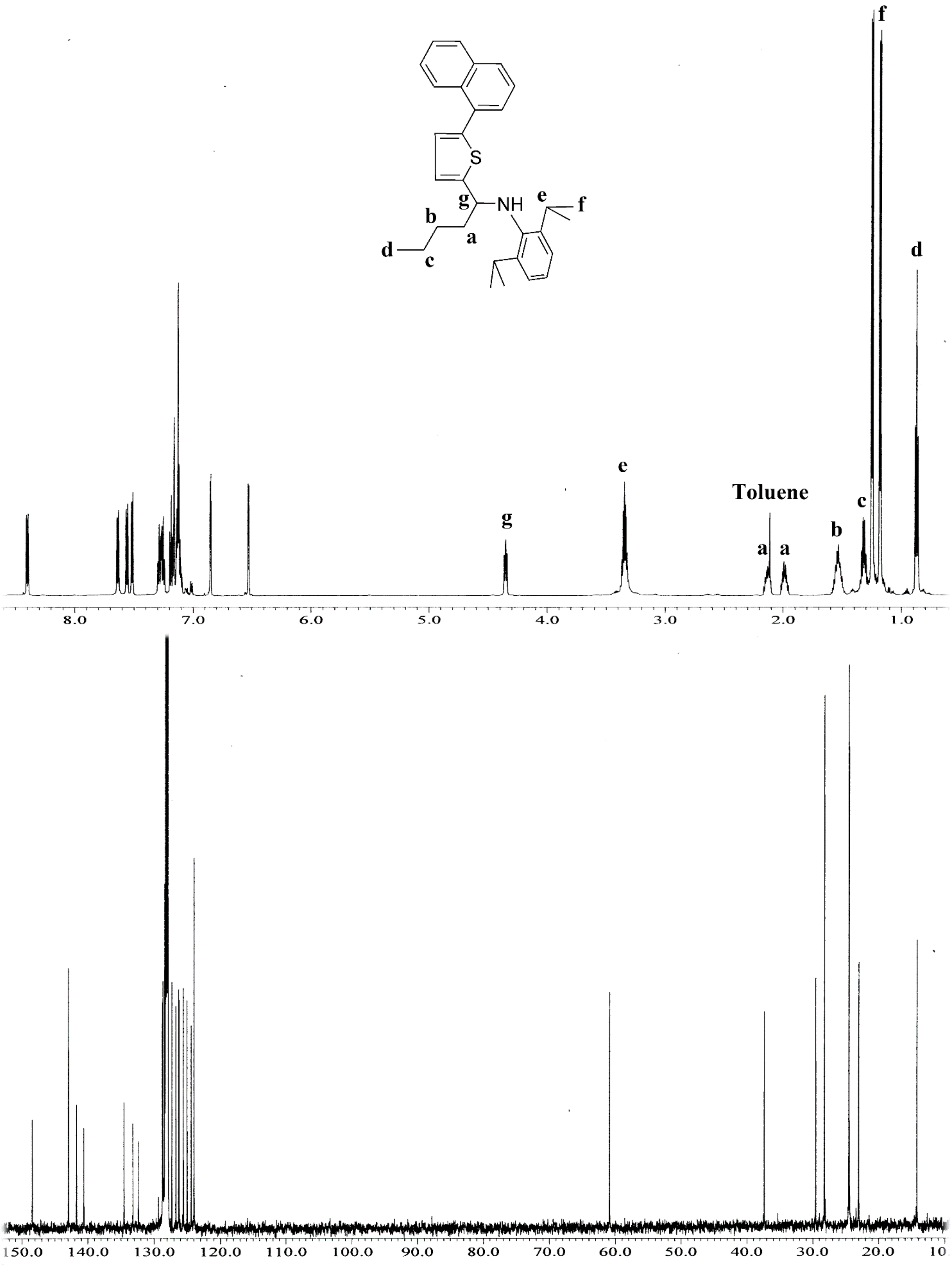

Figure S12. ^{1}H and ^{13}C NMR spectra of **16**

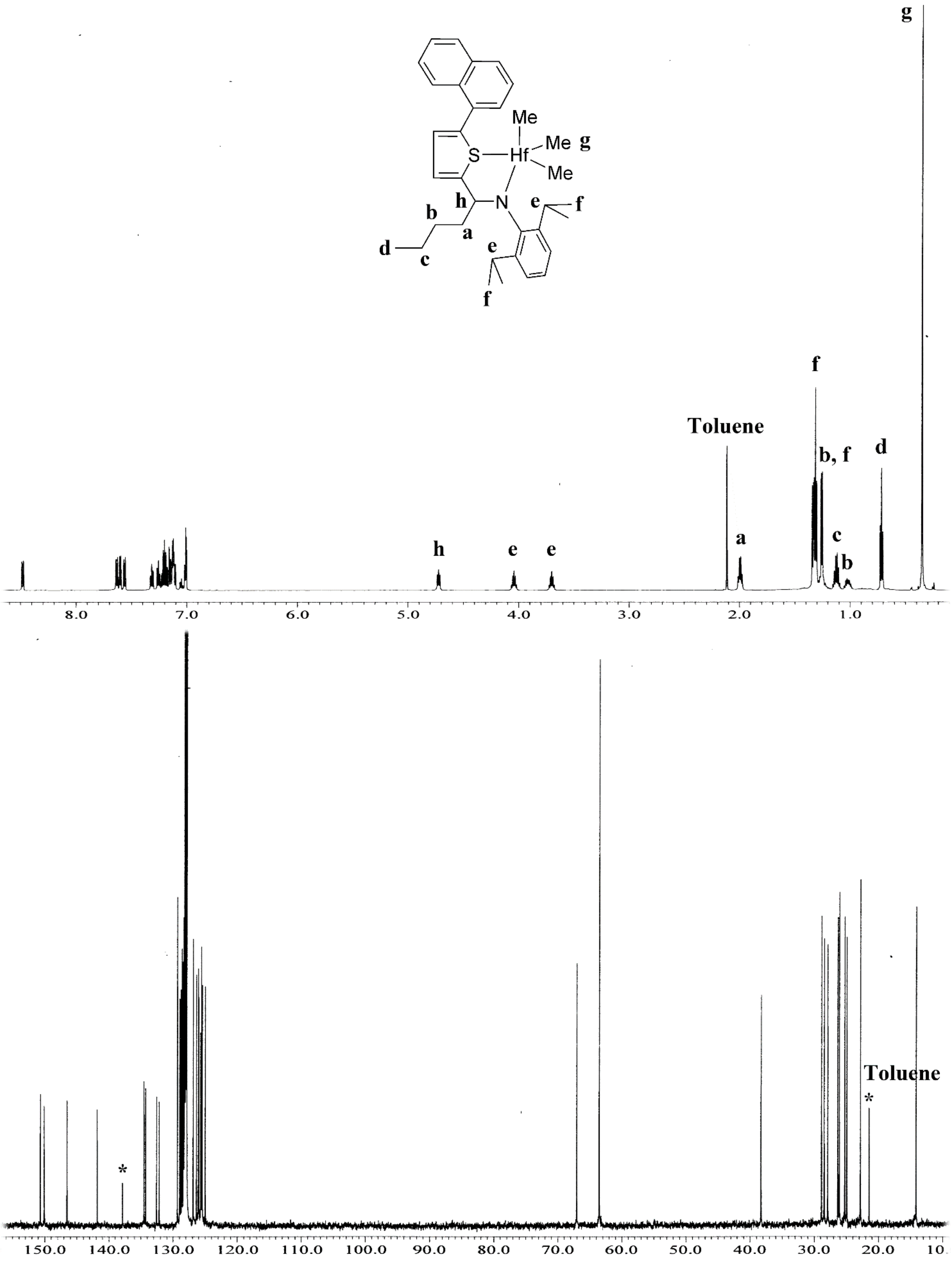

Figure S13. ^{1}H and ^{13}C NMR spectra of **18**

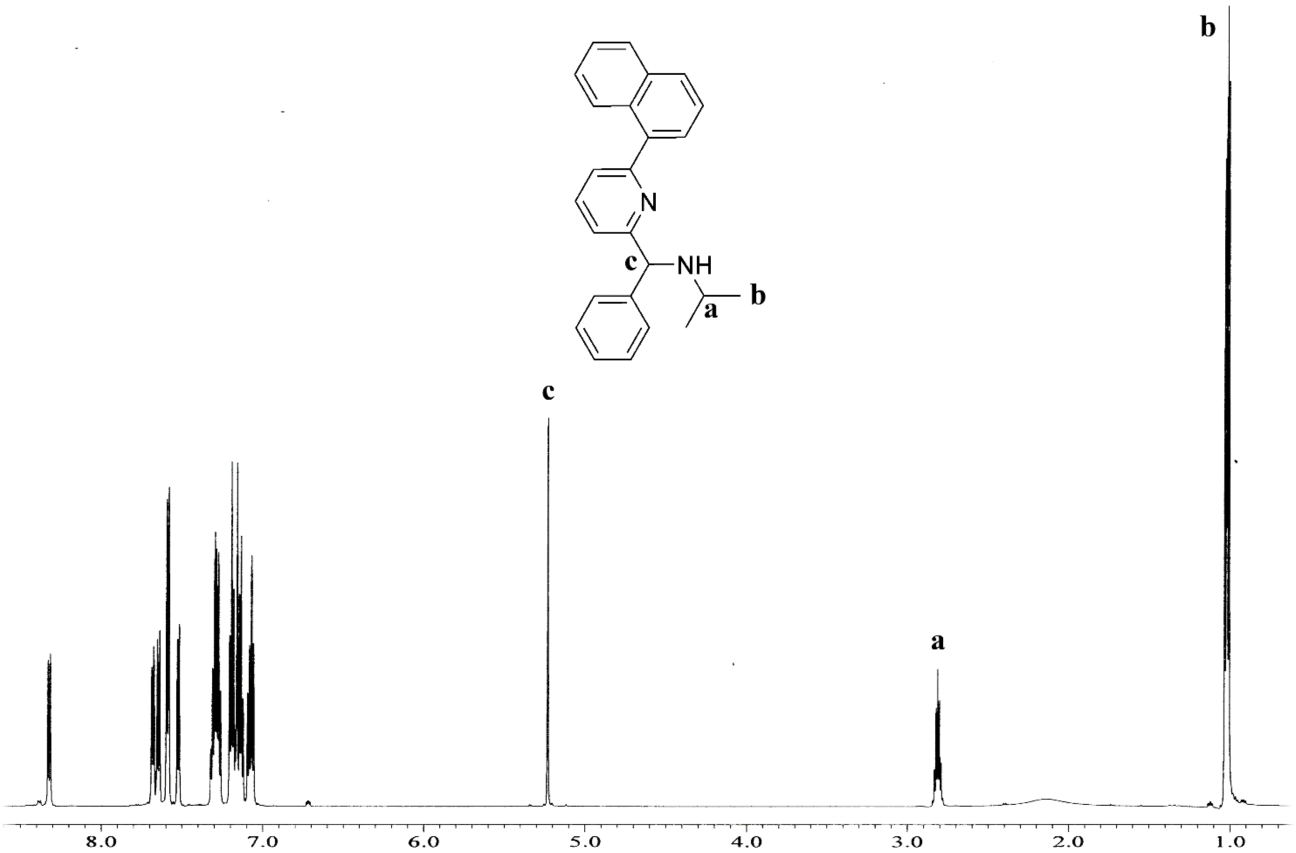

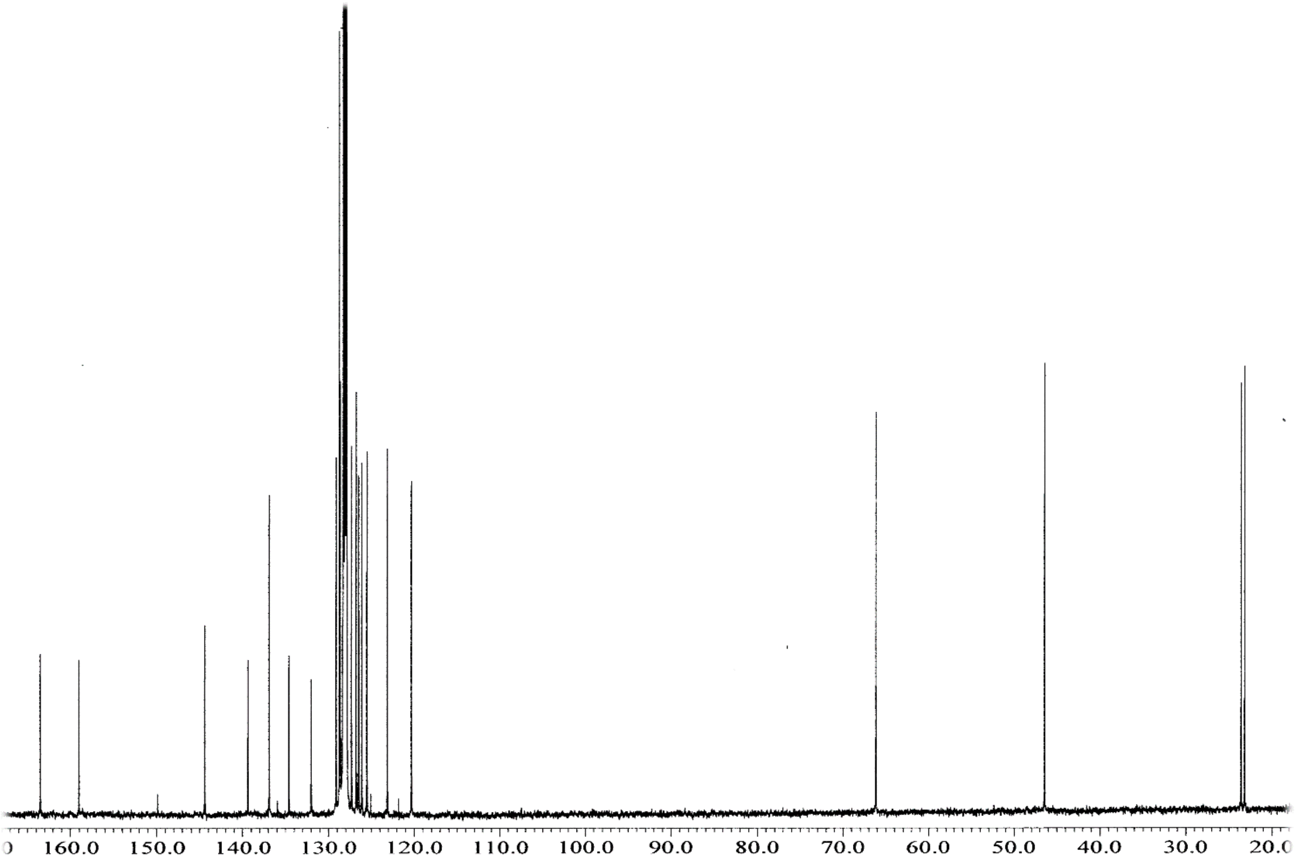

Figure S14. ^{1}H and ^{13}C NMR spectra of **19**

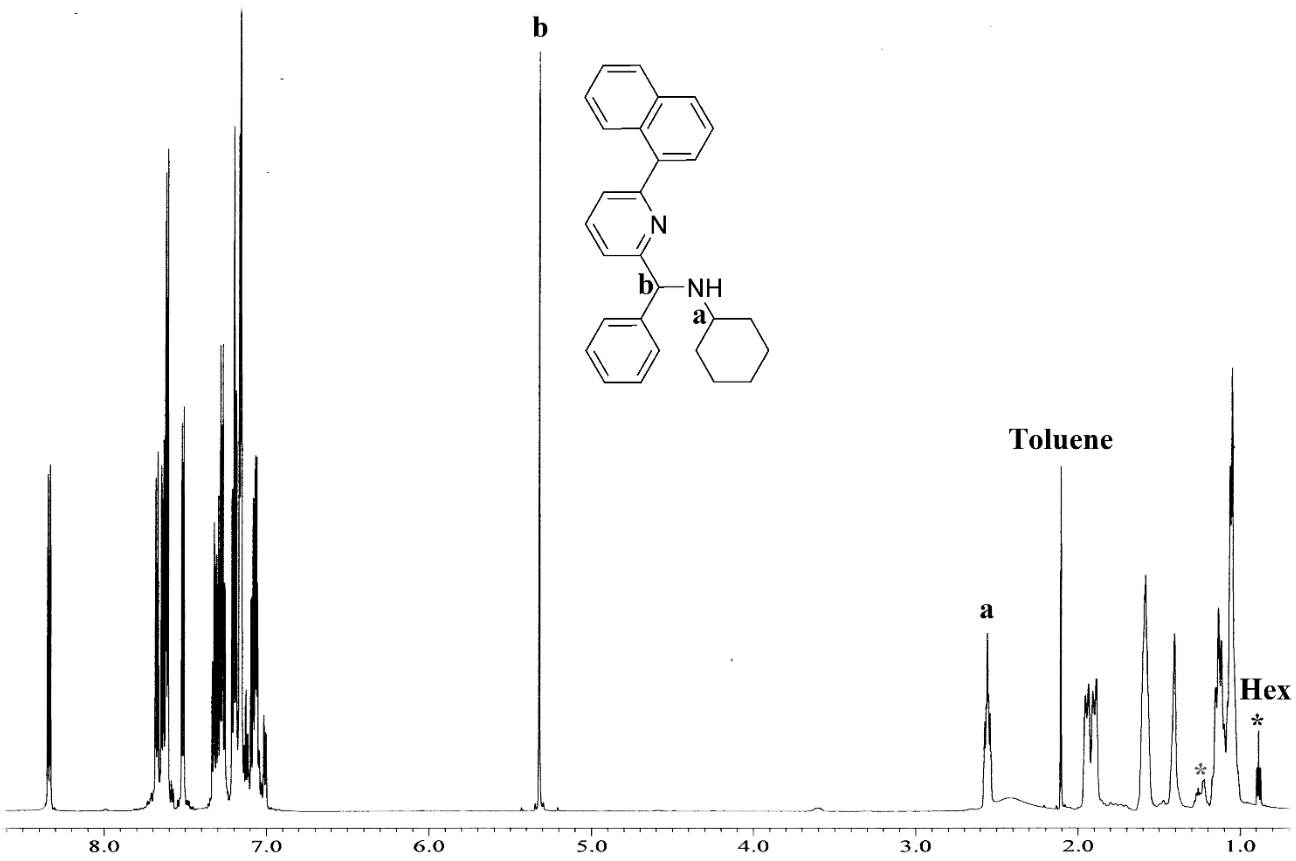

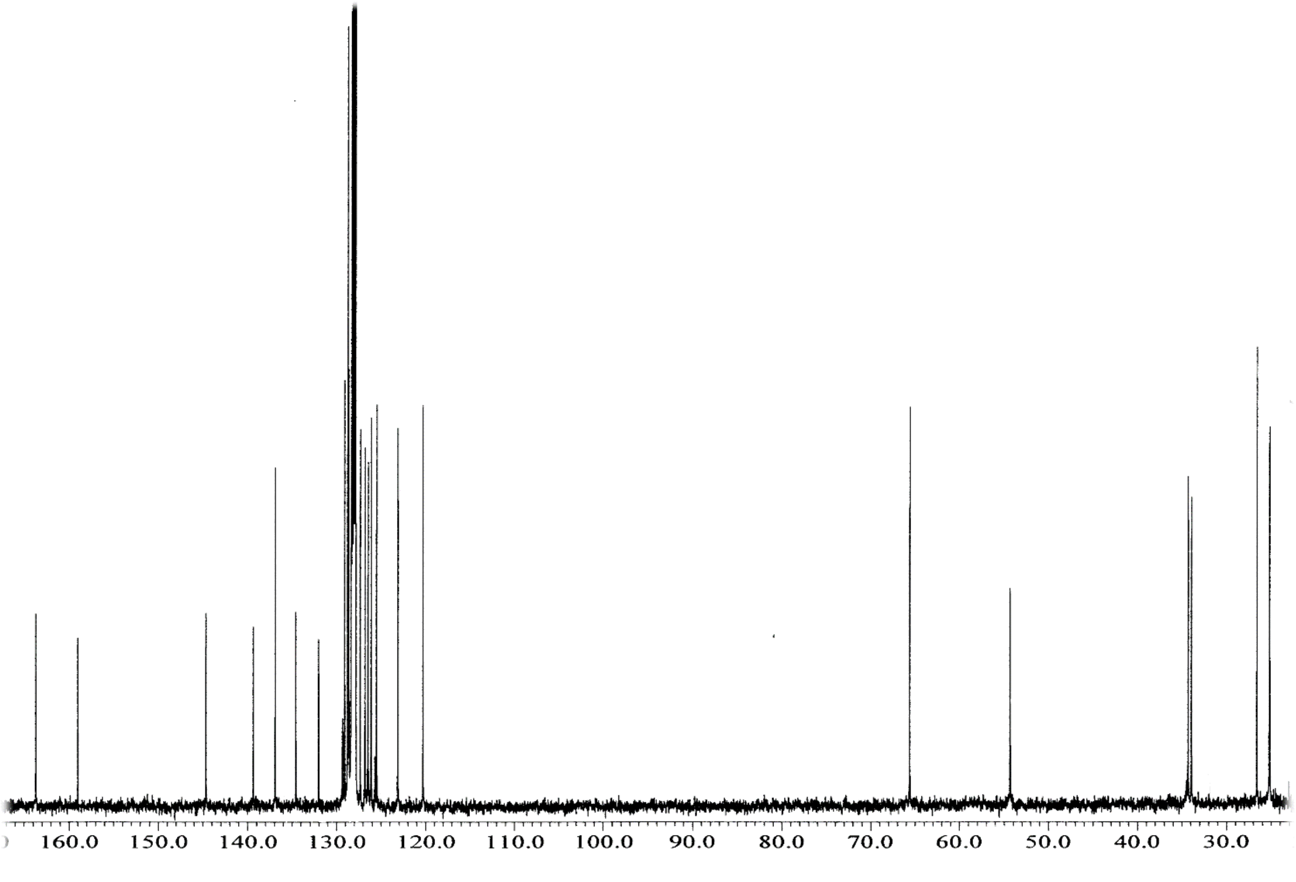

Figure S15. ^{1}H and ^{13}C NMR spectra of **20**

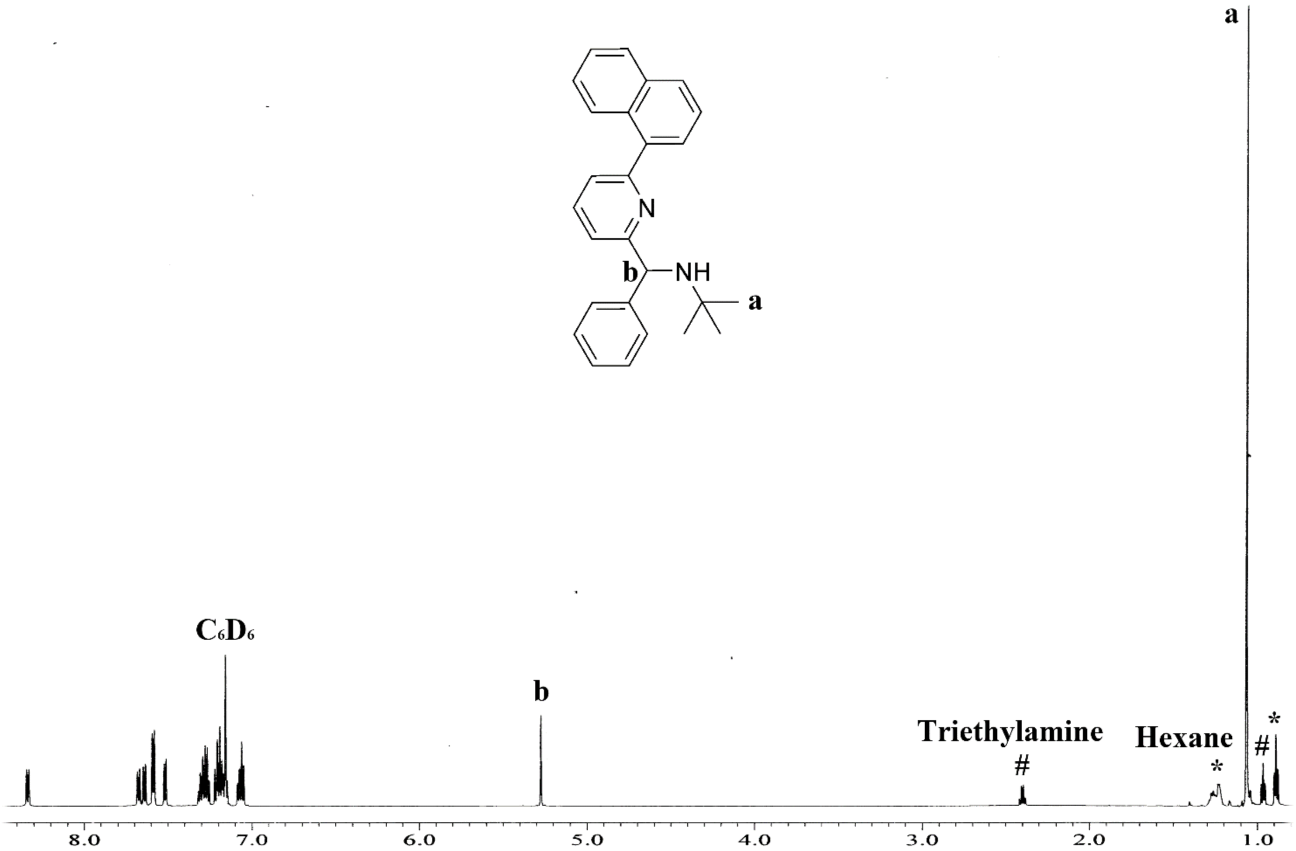

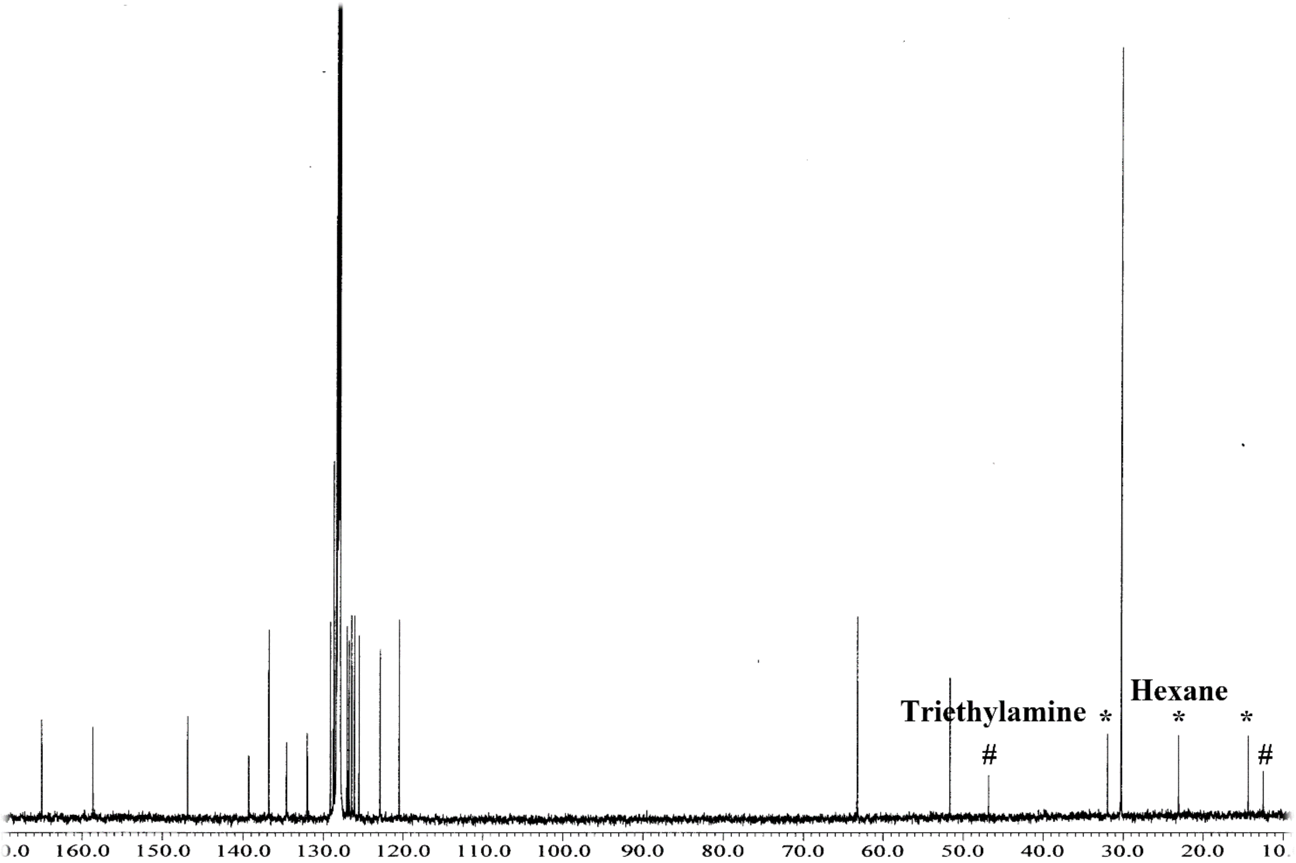

Figure S16. ^{1}H and ^{13}C NMR spectra of **21**

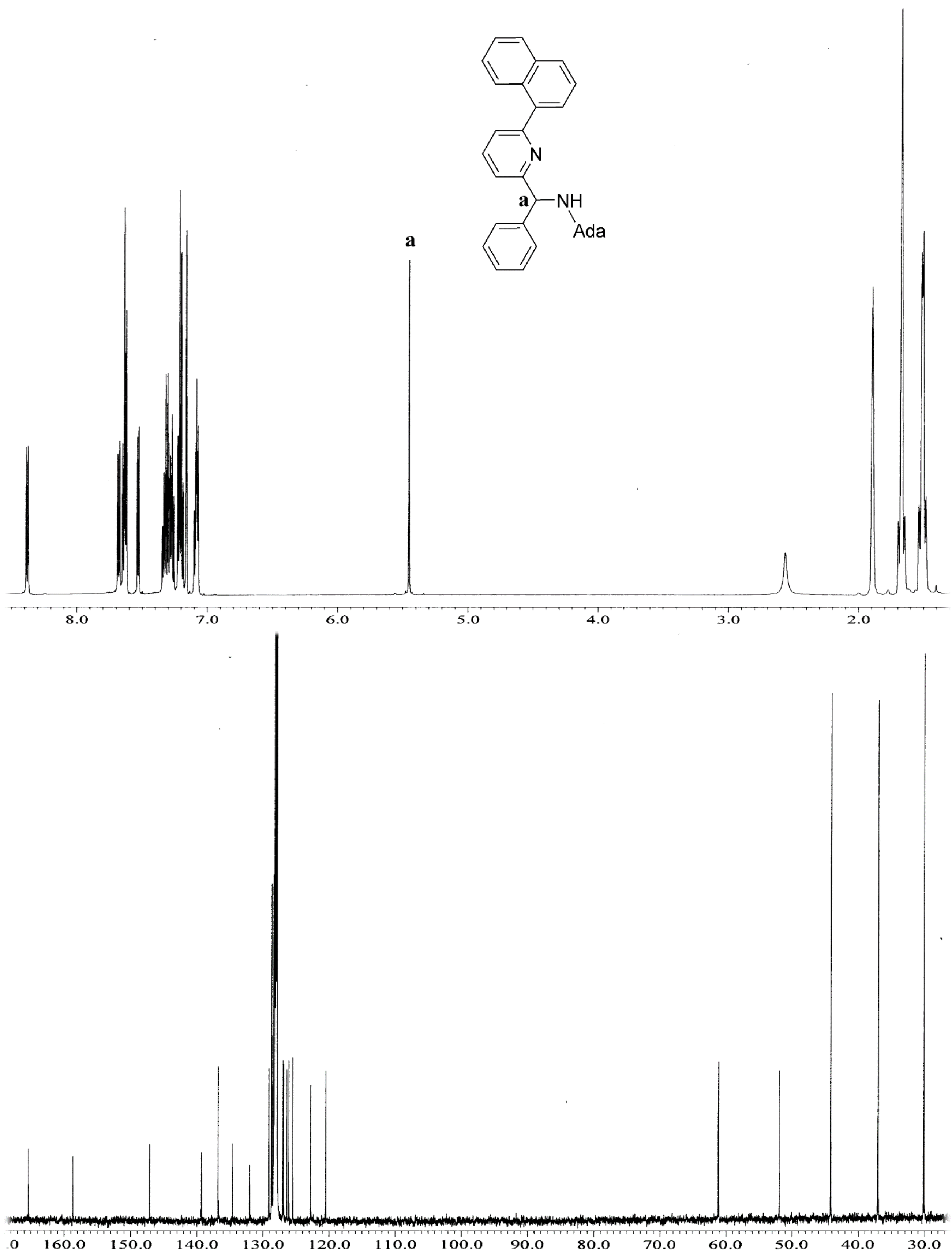

Figure S17. ^{1}H and ^{13}C NMR spectra of **22**

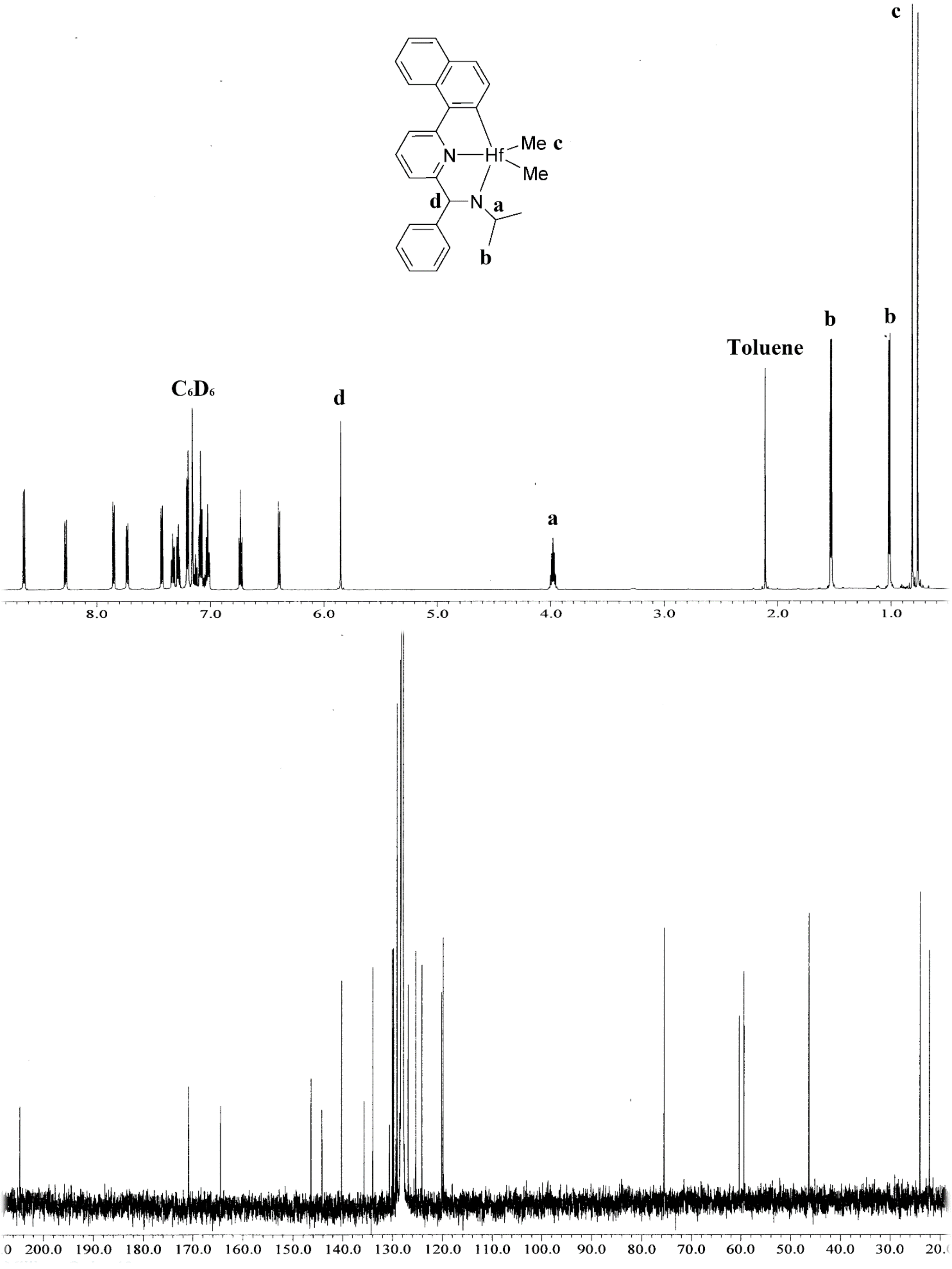

Figure S18. ^{1}H and ^{13}C NMR spectra of **23**

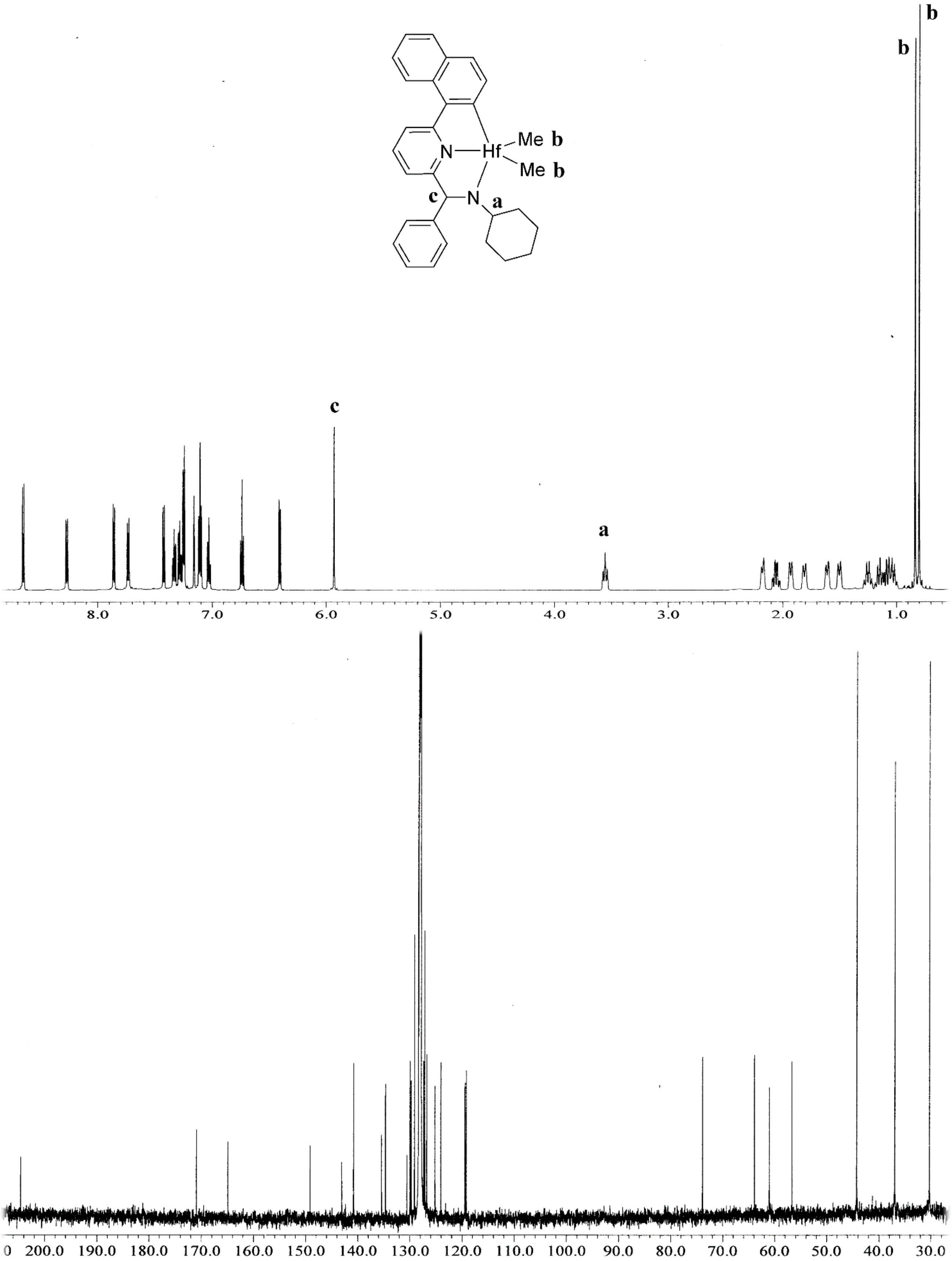

Figure S19. ^{1}H and ^{13}C NMR spectra of **24**

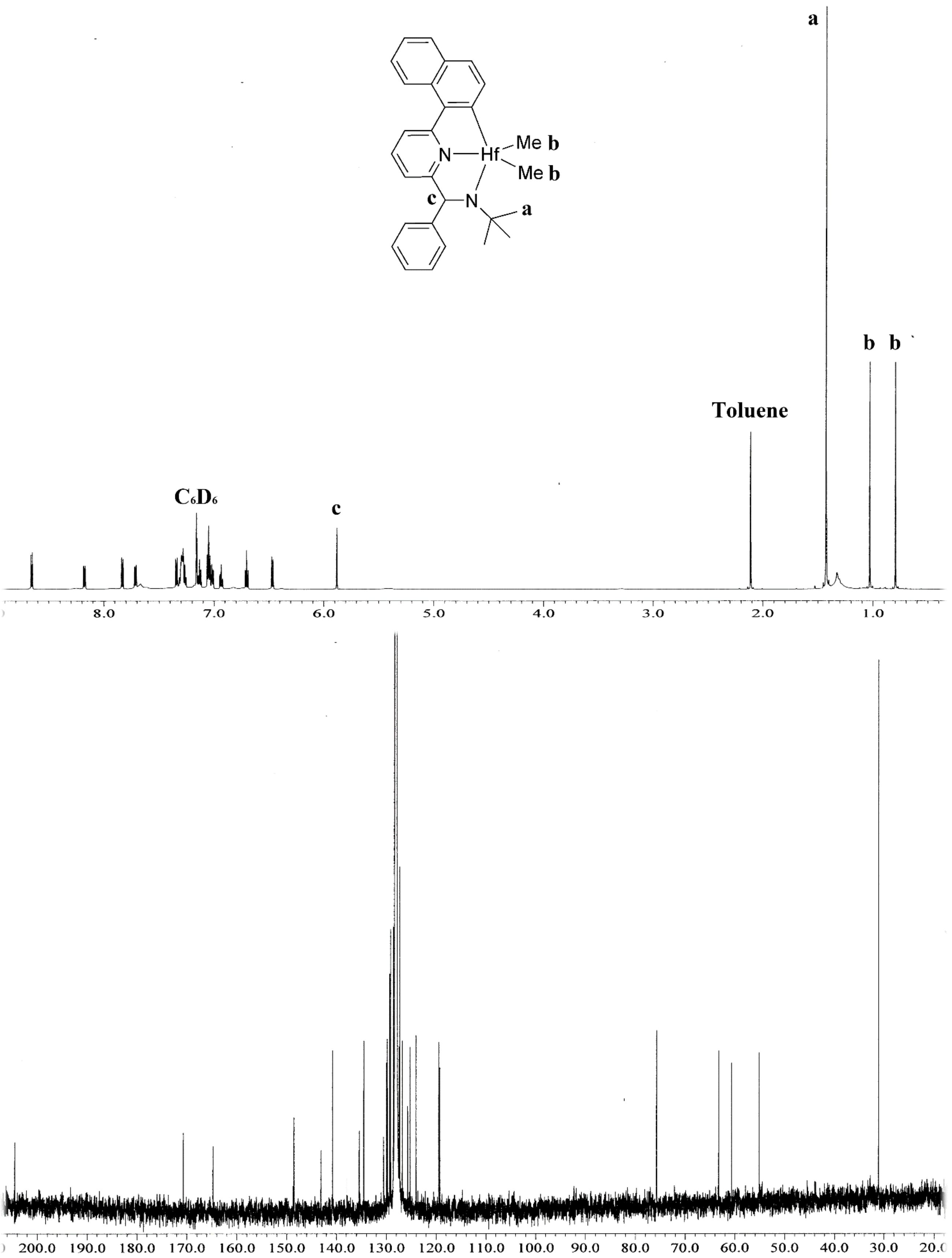

Figure S20. ^{1}H and ^{13}C NMR spectra of **25**

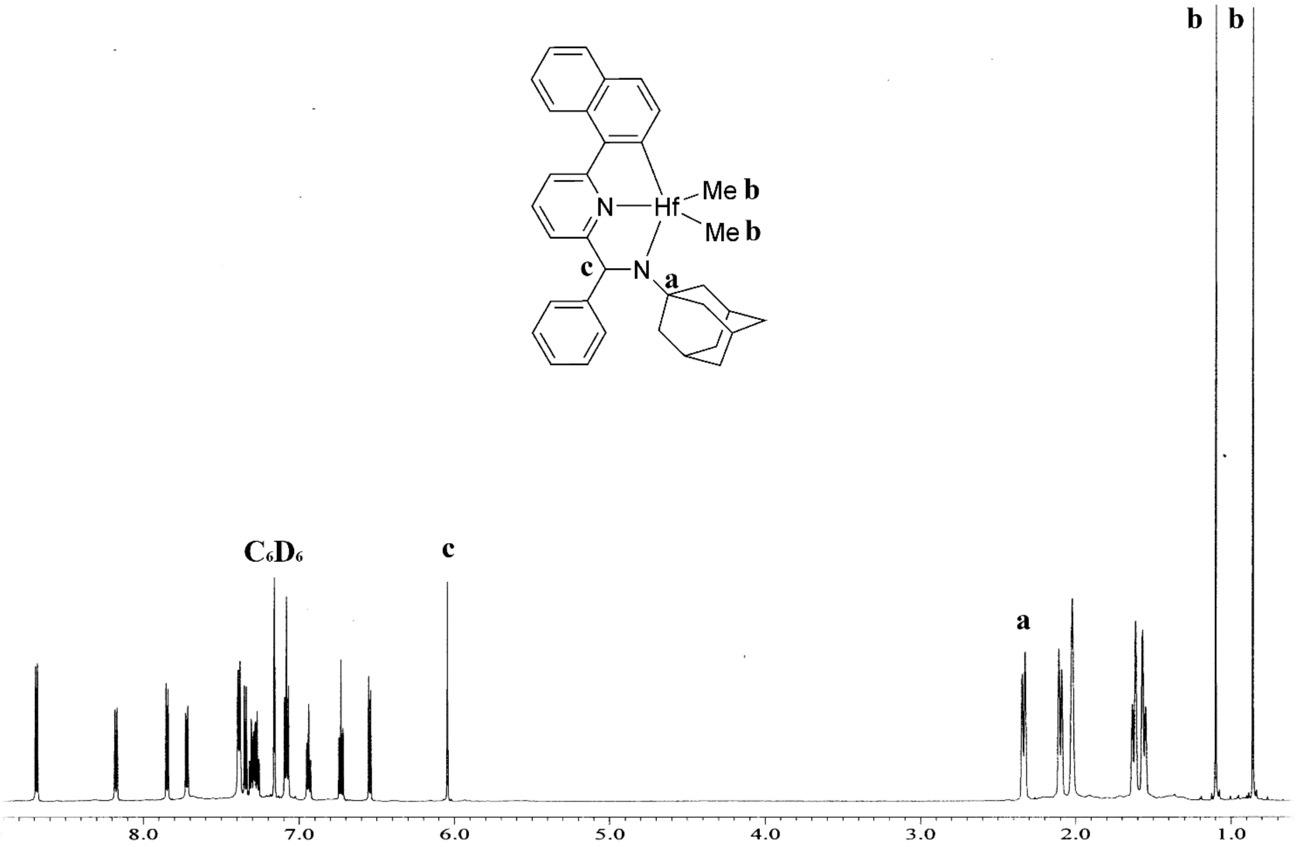

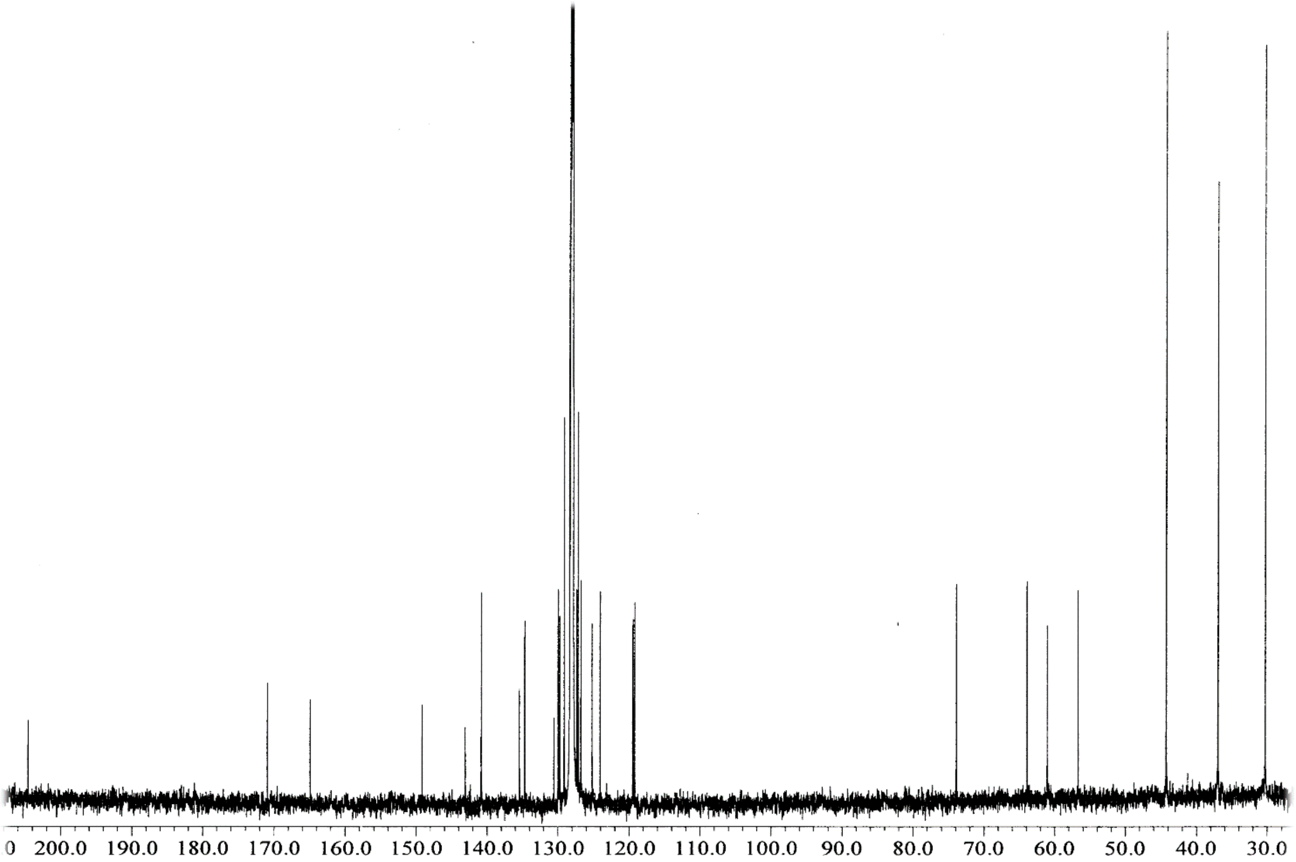

Figure S21. ^{1}H and ^{19}F NMR spectra of **27** measured in 3 h after mixing

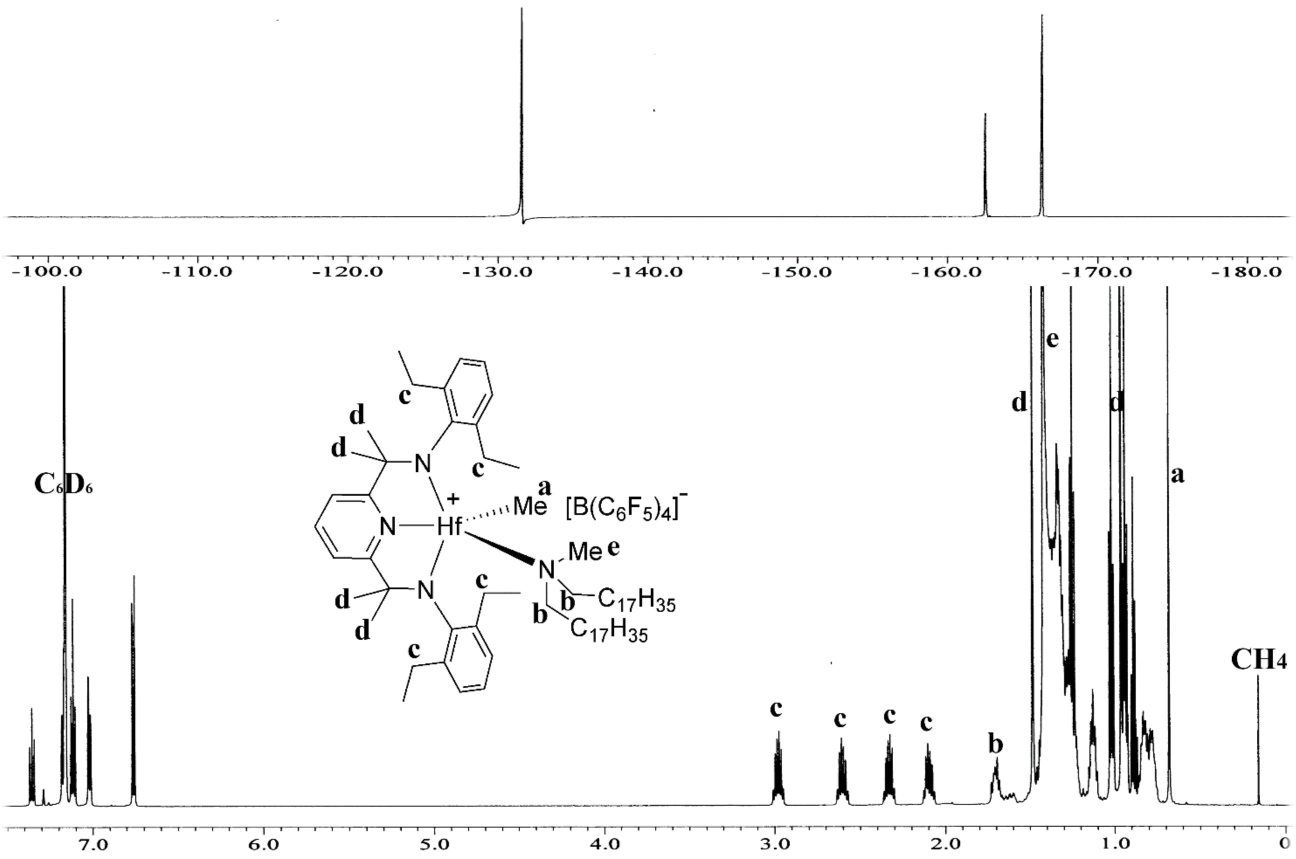

Figure S22. ^{1}H and ^{19}F NMR spectra of **29** measured in 24 h after mixing

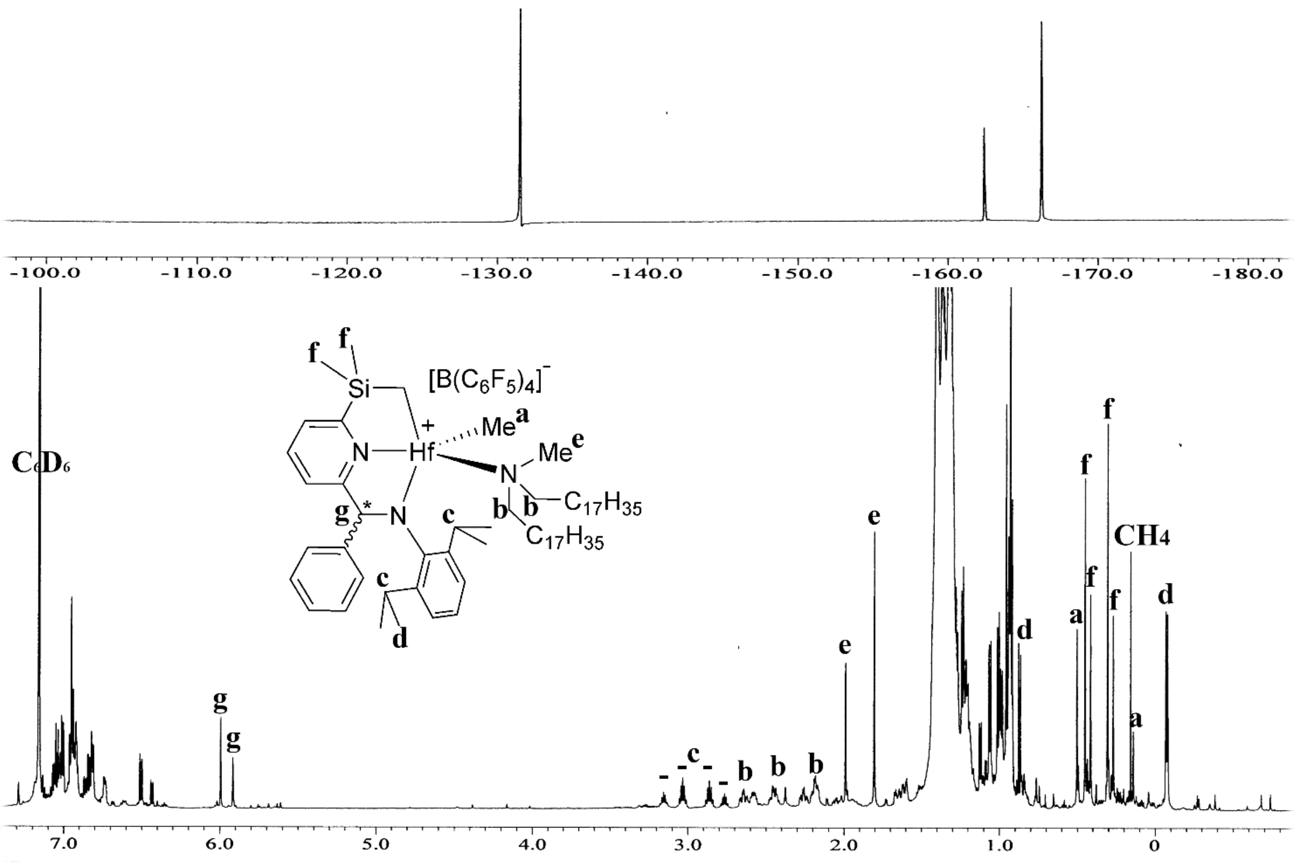

Figure S23. ^{1}H and ^{19}F NMR spectra of **30** measured in 24 h after mixing

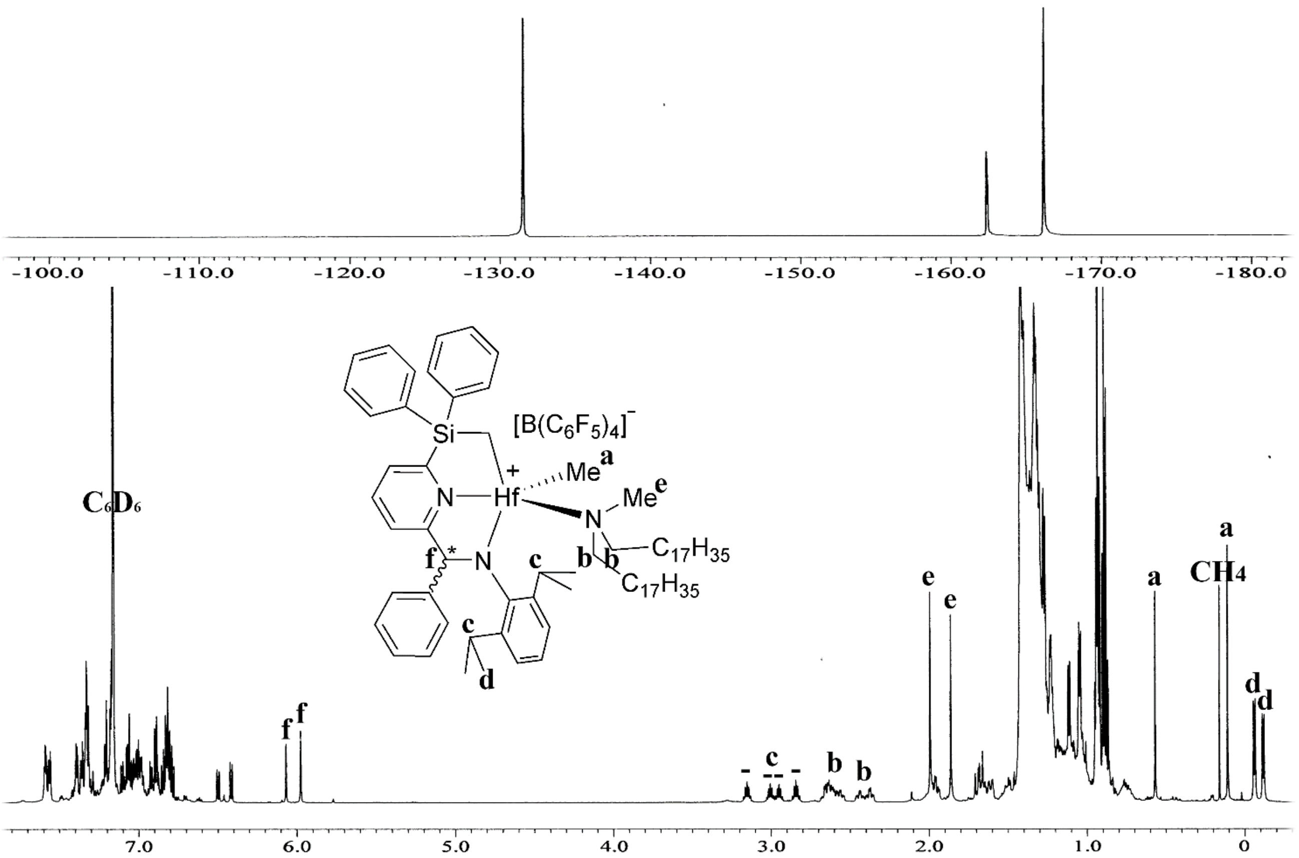

Figure S24. ^{1}H and ^{19}F NMR spectra measured in the reaction of **13** and $[(C_{18}H_{37})_2N(H)Me]^+[B(C_6F_5)_4]^-$ (24 h after mixing)

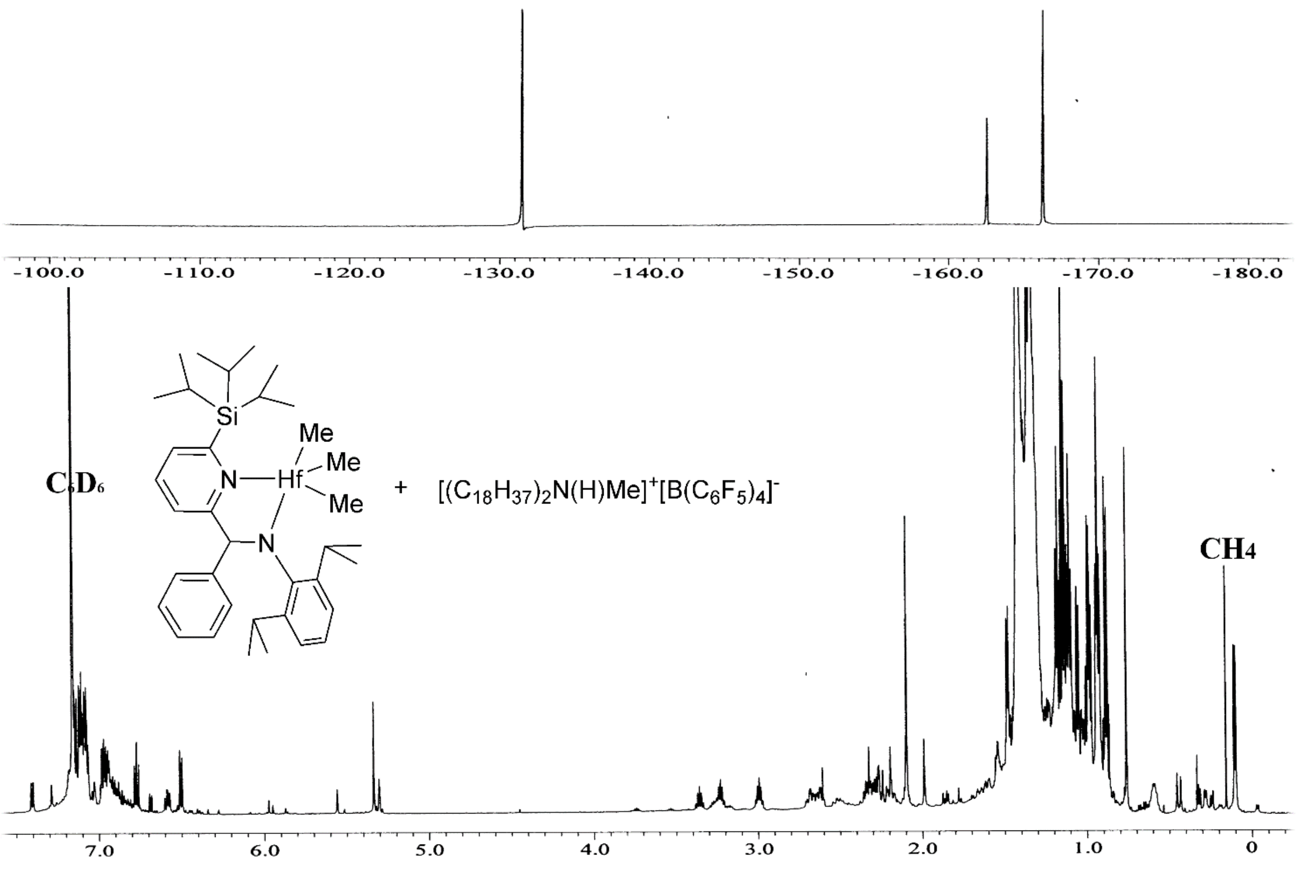

Figure S25. ^{1}H and ^{19}F NMR spectra of **31** measured in 72 h after mixing

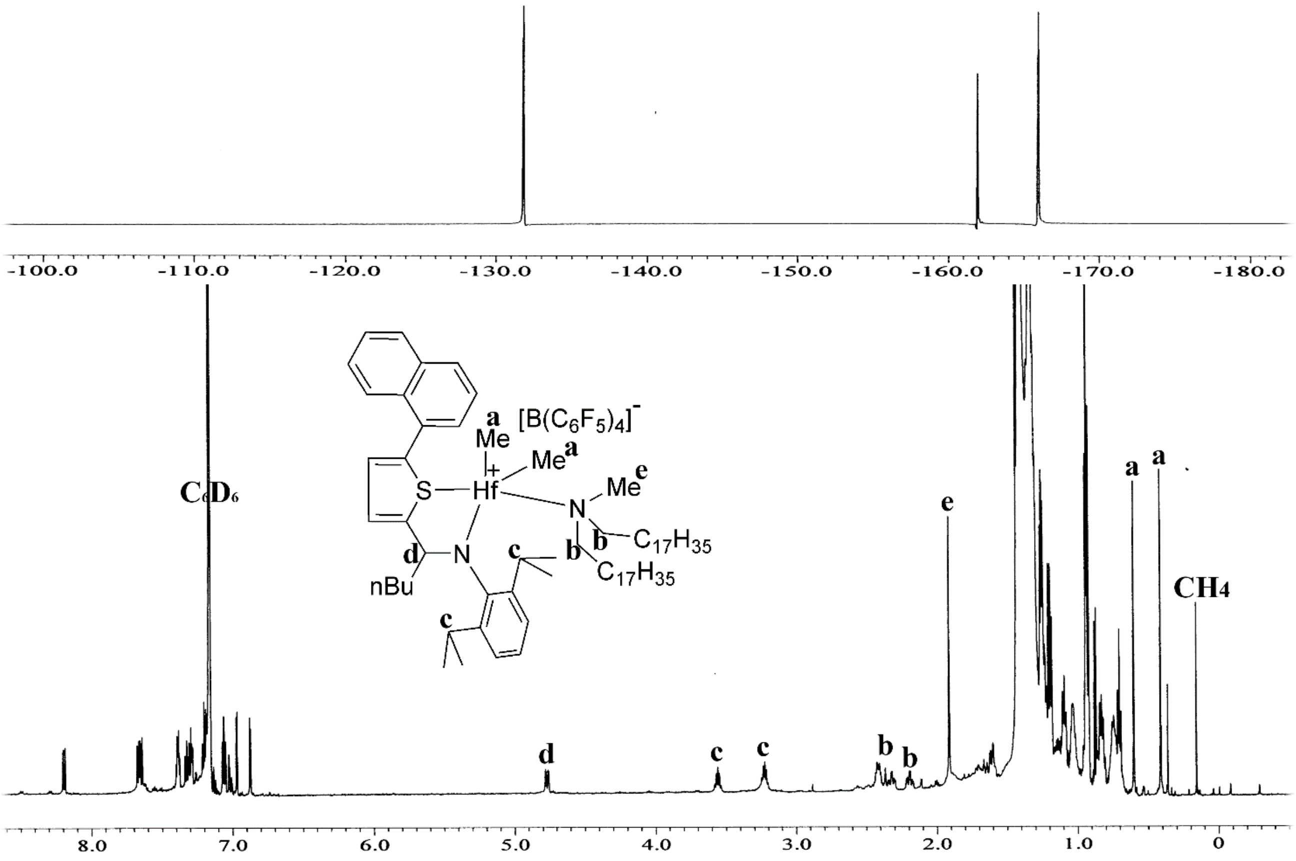

Figure S26. ^{1}H and ^{19}F NMR spectra of **32** measured in 6 h after mixing

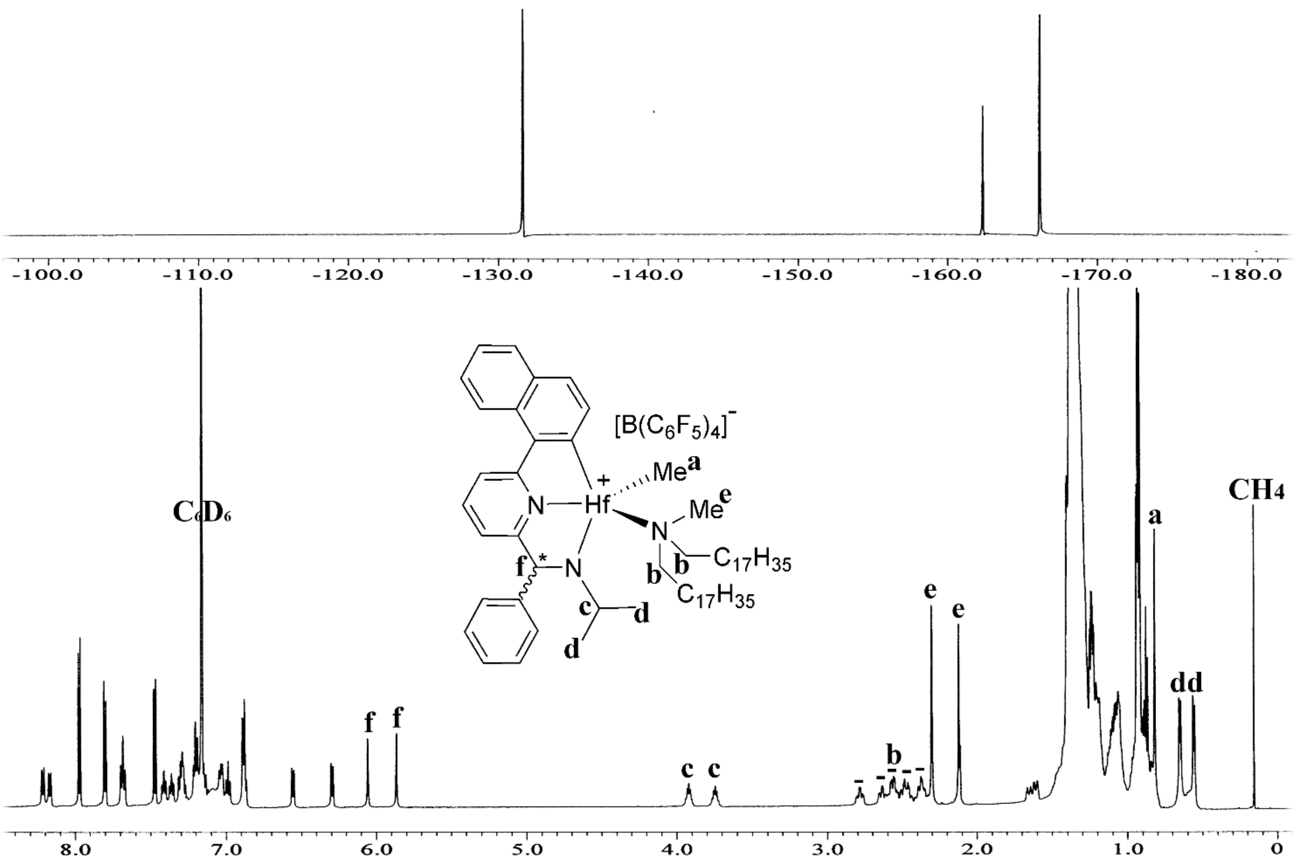

Figure S27. ^{1}H and ^{19}F NMR spectra of **33** measured in 24 h after mixing

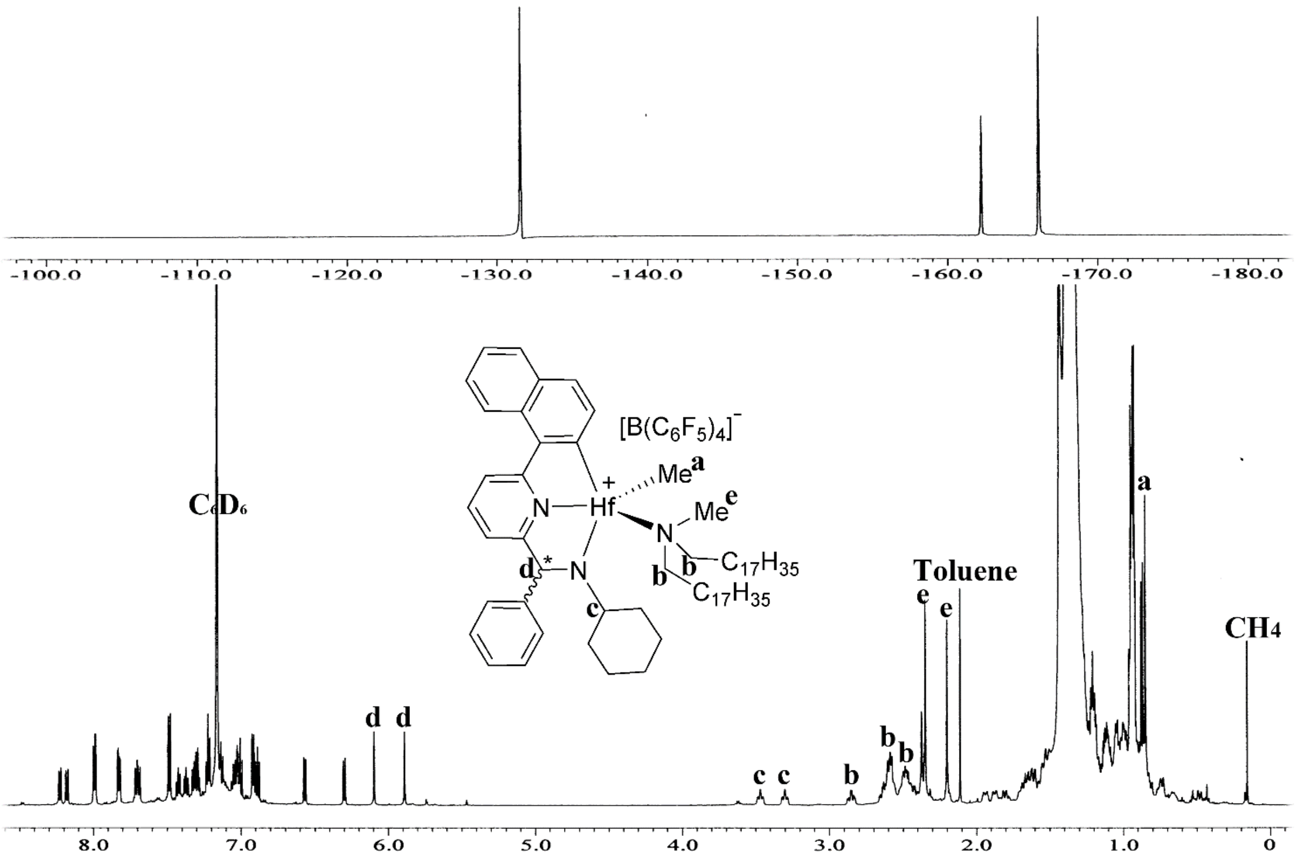

Figure S28. ^{1}H and ^{19}F NMR spectra measured in the reaction of **24** and $[(C_{18}H_{37})_2N(H)Me]^+[B(C_6F_5)_4]^-$ (3 h after mixing)

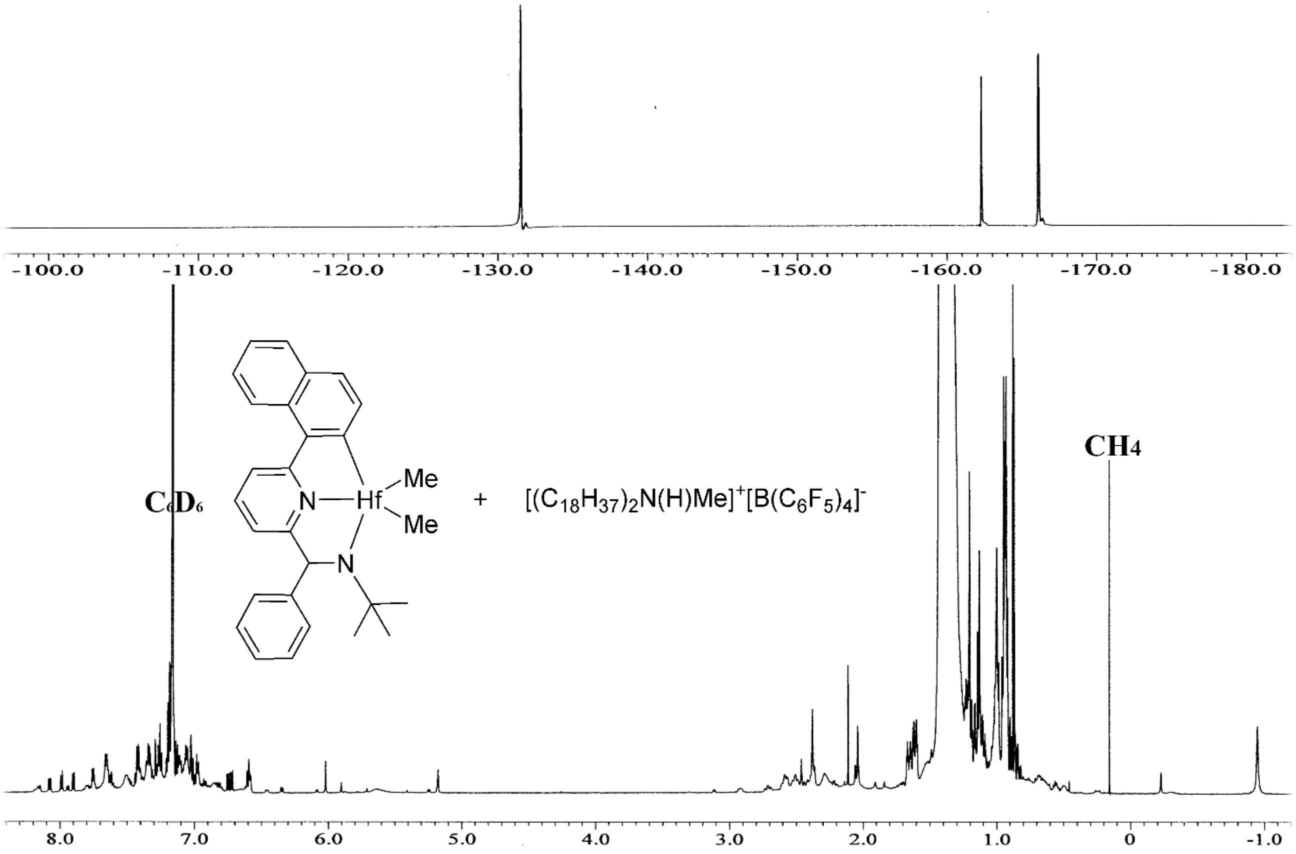

Figure S29. ^{1}H and ^{19}F NMR spectra measured in the reaction of **25** and $[(C_{18}H_{37})_2N(H)Me]^+[B(C_6F_5)_4]^-$ (24 h after mixing)

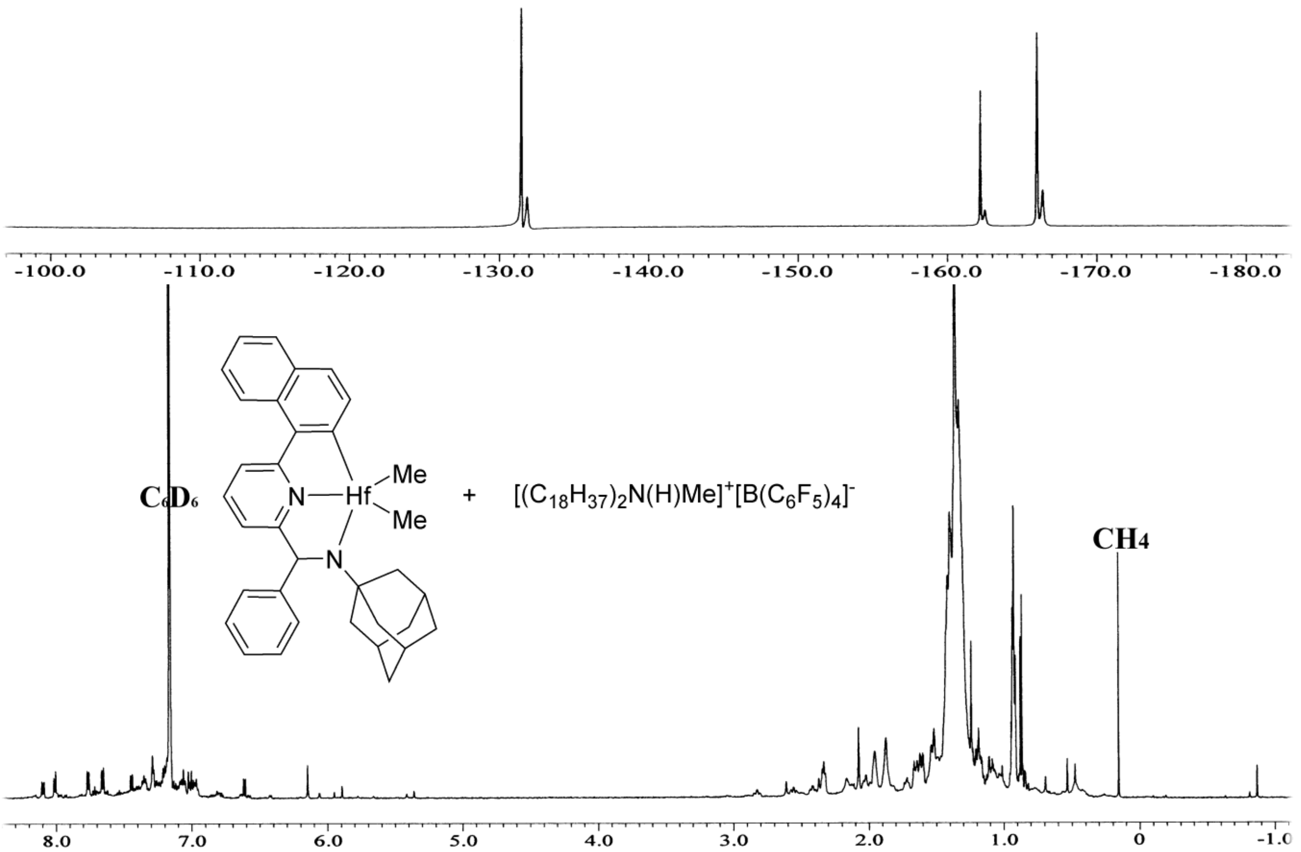

Supplement: Supplementary file 1 [file molecules-24-01676-s001.pdf]
